# Appositive possession in Ainu and around the Pacific

**DOI:** 10.1515/lingty-2021-2079

**Published:** 2021-06-09

**Authors:** Anna Bugaeva, Johanna Nichols, Balthasar Bickel

**Affiliations:** Tokyo University of Science, Tokyo, Japan; National Institute for Japanese Language and Linguistics, Tachikawa, Tokyo, Japan; University of California, Berkeley, USA; University of Helsinki, Helsinki, Finland; Higher School of Economics, Moscow, Russia; University of Zürich, Zürich, Switzerland

**Keywords:** Ainu, appositive, Circum-Pacific, classifier, Pacific Rim, possessive

## Abstract

Some languages around the Pacific have multiple possessive classes of alienable constructions using appositive nouns or classifiers. This pattern differs from the most common kind of alienable/inalienable distinction, which involves marking, usually affixal, on the possessum, and has only one class of alienables. The Japanese language isolate Ainu has possessive marking that is reminiscent of the Circum-Pacific pattern. It is distinctive, however, in that the possessor is coded not as a dependent in an NP but as an argument in a finite clause, and the appositive word is a verb. This paper gives a first comprehensive, typologically grounded description of Ainu possession and reconstructs the pattern that must have been standard when Ainu was still the daily language of a large speech community; Ainu then had multiple alienable class constructions. We report a cross-linguistic survey expanding previous coverage of the appositive type and show how Ainu fits in. We split alienable/inalienable into two different phenomena: argument structure (with types based on possessibility: optionally possessible, obligatorily possessed, and non-possessible) and valence (alienable, inalienable classes). Valence-changing operations are derived alienability and derived inalienability. Our survey classifies the possessive systems of languages in these terms.

## 1 Introduction

A number of languages around the Pacific and throughout the Americas use head-marking person or person-number affixes to index possessors on nouns of a class usually called *inalienable*, a restricted set of nouns that most often obligatorily take possessive morphology. The inalienables vary from language to language but usually include body parts, kin terms, and/or a few other nouns intimately connected to human owners (articles of clothing, essential implements and weapons, ‘house’, etc.). In most current thinking, inalienables have argument structure and the possessor noun is the argument. In most languages with an alienable/inalienable distinction, alienables differ from inalienables in not being obligatorily possessed, and/or in having a distinctive and often longer form of possessive morphology, and/or in using dependent-marked morphology, typically a genitive case on the possessor noun, while inalienables are head-marked or juxtaposed to (or compounded with) the possessor (Nichols 1988). But in a number of the languages at issue here the alienables – a large and open class of nouns – cannot take morphological indexation, even when the referent of the noun in question is owned or otherwise possessed. That is, these are *non-possessible* nouns. Instead they use an analytic construction in which a word in an appositive relation to the possessed noun bears morphological possessive marking, and often also has classificatory functions (Nichols and Bickel 2005a, 2005b). We call this the *appositive word*. Usually this appositive word is an inalienable noun taking the regular possessive inflection of inalienables. In (1) are examples from Fijian, where *no-* is a classifier noun taking a possessive suffix and in apposition to ‘house’.

(1)

**Table:** 

Fijian (Gottocode: fiji1243, Austronesian, Fiji; Lynch et al. 2002: 40)				
Inalienable		Alienable		
*na*	*mata-qu*	*na*	***no*** *-qu*	*vale*
ART	eye-1sg	ART	**CL**-1sg	house
‘my eye’		‘my house’		

In most languages the appositive word can also function as an independent noun. In some languages, however, the appositive classifiers are verbs, either finite verbs or relativized or nominalized to enable them to modify the possessed noun. Here we show that Ainu (ainu1240), an isolate of northern Japan and formerly also Sakhalin and the Kuril Islands, has an appositive classifier system that uses verbs. Ainu inalienables, chiefly body parts and kin terms, take direct affixation of possessive affixes, while alienables cannot take person markers but instead use a relative construction with ‘have’ in which the possessor of the head noun is marked as subject of ‘have’ and the possessed noun is the (gapped) object of ‘have’.

Ainu (Bugaeva 2004, 2012; Satō 2008: 156)

(2)

**Table:** 

*(káni)*	***ku-*** *sik* ***-i***
1SG	1SG.A-eye-PSD^1^
‘my eyes’	

1 This and other abbreviations and terms introduced here are defined in §2.2. Others are either familiar, transparent, or defined in place.

(3)

**Table:** 

[ __	*ku* ***-kor***]	*seta*
O	1SG.A-have	dog
‘my dog’, lit. ‘dog (that) I have’^2^		

2 Here and below, an underscore indicates the gap in gap-strategy relativization. It is interlinearized with the argument role of the gapped nominal.

As discussed below, Ainu ‘have’ in alienable possession appears to be the survivor of a formerly larger set of verbs with classificatory functions. The person markers used in this possessive construction are unambiguously *verbal* person markers. Does the Ainu construction fit in with the Oceanic and American languages as an appositive alienable possessive construction? Or is it simply a rare or unique treatment of alienable possession? This paper surveys appositive possessive constructions around the Pacific and inland to define the grammatical and geographical range, limits, and bounds especially of the verbal type, to show how Ainu does and does not fit the established typology, and to address the hypothesis of Nichols and Bickel (2005a, 2005b) that this is a Pacific Rim trait. In what follows we provide a method and theoretical framework (§2), describe Ainu in those terms (§3), account for some of the typological behavior (§4) and distribution of appositive possessive constructions (§5), and close with open questions (§6). There are three Online Supplementary Material: S1 lists the languages surveyed and their possession types; S2 gives figures and methods for the statistical analysis; and S3 gives analysis and examples of possessive constructions from all survey languages found to have appositives.

## 2 Method and survey design

### 2.1 Survey

We started with a close, typologically based description of the Ainu possessive system (§3) and extracted from it the set of features and concepts needed to determine, point by point, whether the Ainu system is or is not of the same type as known appositive systems. We then carried out an in-depth cross-linguistic survey of the target constructions in languages around the Pacific and nearby to refine the typology and map out the distribution, among other things surveying sisters of languages with appositive possession to see if they also have the constructions. We performed a less in-depth survey of other continents. The resultant survey is larger and better targeted than the WALS surveys of Nichols and Bickel (2005a, 2005b). Like those surveys, it covers only common nouns as heads of possessive constructions, leaving out proper names because their referents are not often possessed.3Names are occasionally mentioned as not possessible (an example is Dutton 1996: 40–41 for Koiari (gras1249); his term is “unpossessable”), but we have not counted these as non-possessibles. Dutton classifies terms for animals as proper names, together with personal names, place names, and ethnonyms.


The geographical macroareas are defined as follows (Bickel et al. 2017; Nichols et al. 2013). The *Pacific Rim* is the population of languages from northern Australia to (clockwise) southernmost South America, from the coast to the upper far side of the major coastal range. The *Circum-Pacific* population is the Pacific Rim population plus all of Australia, New Guinea, and the Americas.

### 2.2 Key grammatical concepts

Grammatical terms used here, and their definitions, are as follows.4Note that in this set of terms we are not defining the ordinary English verbs ‘own’ and ‘possess’ but technical terms in which one or the other of the roots *own* and *possess* figures as one etymological building block. Terms we use in abbreviated form in the text are listed by those abbreviations (which follow Hill 2014; as she does, we use the abbreviations as interlinear glosses).

Possession: The relation of an adnominal dependent (including ones with appositive structure) to a head noun which the dependent owns, possesses, or has, or which belongs to the dependent.5Here and below, wording like “the possessor owns the head noun” is shorthand for “the referent of the possessor owns the referent of the head noun”. Typically the semantics involves ownership, other entitlement, part-whole relations, kinship relations, etc.
Possessive: We use this adjective to describe an NP or DP with an adnominal noun (*possessive phrase*) and semantics of possession, and/or with head-marking morphology marking the possessive relation and indexing properties of the possessor (e.g. *possessive suffix*). Head-marked possessive indexation almost always involves person or person-number.
Possessor: The dependent element in a possessive phrase.
Possessum: The head noun in a possessive phrase.
Optionally possessible (often shortened to *possessible)*: A noun or noun class that may, but is not required to, have possessive morphology.
Obligatorily possessed: A noun requiring a possessor as argument, and obligatorily grammatically possessed; typically this means obligatorily taking a head-marked possessive affix or clitic. These are then bound nouns, grammatically unable to occur without possessive marking.
Non-possessible: A noun that grammatically cannot take possessive inflection (in general, as a matter of lexical morphological principle) and therefore cannot take head-marked person indexation in languages that mark possession that way.
Owned non-possessible: A grammatically non-possessible noun whose referent is owned by or belongs to someone (or something, but most examples involve human or anthropomorphized possessors).
Possessibility: General term for the macro-category of possessible, obligatorily possessed, non-possessible. Importantly, the terms for the possessive classes *possessible, obligatorily possessed,* and *non-possessible* are grammatical classificatory terms, not semantic ones. In using a possessive construction, a speaker does not decide to convey whether an item can be owned or not; rather, the choice of a noun determines the possessive construction that can be used with it. Some of the cross-linguistically most common non-possessibles are words whose referents can be and usually are owned, such as domestic animals, especially dogs. Cahuilla (cahu1264) (§4.2.1) is a good example: the non-possessible classes are defined by traditional categories of ownership and usufruct.
Alienability: A formal morphological opposition between nouns which can or must take possessive marking when possessed (=when head of a possessive construction) and those which do not or cannot or take a different formal class of possessive marking. It is a formal opposition in how heads of possessive NPs are marked, and not a semantic type of possession (though grammars often offer a rough semantic characterization of the distinction).
Inalienable: A noun or noun class belonging to one of two values of an alienability opposition. Most often *inalienable* is applied to obligatorily possessed nouns. Sometimes the distinction is based on types of marking: e.g. inalienables have head-marked possession while alienables variously use a different set of possessive allomorphs, or juxtaposition, or dependent-marked possession (e.g. a genitive case). (Nichols 1988 gives a full inventory of types). The definition of what counts as *inalienable* is specific to individual languages and we do not further typologize the lexical choices here.
Alienable: A noun or noun class belonging to the other member of the alienability opposition. Most often it refers to optionally possessible nouns, but sometimes also or rather to non-possessibles.
Appositive noun (here often shortened to *appositive)*: In a possessive construction, a noun or noun-like possessive morpheme that is in apposition to a non-possessible head noun and carries the morphological marking of possession. Usually it carries possessive morphology; often it is an obligatorily possessed noun; sometimes it is a dedicated classificatory morpheme but in some languages it can function as a head noun on its own. Examples are in (4) and (5). In (4a) and (5a), ‘animal’ functions as the (possessed) head noun; in (4b) and (5b) it is in apposition to another noun, which we take to be the head of the constituent (‘pet’ and ‘cat’, respectively).

(4)

**Table:** 

Macushi (macu1259, Cariban, Brazil; Carson 1982: 58)

a.

**Table:** 

*u-y* ***-ekïng***	*wuty*
1sg-POSS-**animal**	go
‘I will ride my animal’	(independent noun; inalienable)

b.

**Table:** 

*woroké* *u-y* ***-ekïng***
parrot 1sg-POSS-**CL:**pet^6^
‘my parrot’ (classifier; ‘parrot’ is non-possessible)

6 Here and below, we gloss the classifier for domesticated animals ‘pet’ where the sources do. The classifier usually applies to more nouns than would be called pets in English.

(5)

**Table:** 

Wichi Lhomtes (wich1263, Matacoan, Argentina; Nercesian 2014: 169, 168)

a.

**Table:** 

*la-* ***lo*** *=y*
3-**animal**=PL
‘the animals’ (independent noun *lo* ‘animal’)

b.

**Table:** 

*n’-* ***lo*** *=mitsi*
1-**CL**:animal=cat
‘my cat’ (classifier; ‘cat’ is non-possessible)

Appositive verb: An independent verb that functions as a classifier to modify a possessed noun and carry possessive marking. An example is (3) above, where the finite verb ‘have’ carries the marking of possession which is not available to non-possessible ‘dog’. The appositive verb is finite in Ainu and some other languages, but in others it is nominalized or a participle, enabling it to modify the possessed noun. We nonetheless call it a verb, assuming that the nominalizing or participial morphology is inflection dictated by the syntax and not derivation of a word with its own part of speech. In contrast, there are also cases where appositive nouns are etymologically related to verbs or synchronically derived from verbs by means of derivational morphology or conversion, and we regard these as separate words of the nominal part of speech rather than inflected forms of verbs. Grammar descriptions are usually adequate to let us make these decisions unproblematically.
Possessive classifier: Any morpheme that is an element of the possessive construction and whose form is dictated by the possessed noun, regardless of its lexical or grammatical type (purely classificatory clitic or affix, appositive lexical noun, etc.). For convenience we shorten the term to just *classifier,* unless other types of classifiers are also at issue. Interlinear: CL:, with the name or gloss of the class following the colon.
NPN (non-possessed noun): Morphology that derives an alienable from an inalienable or allows an otherwise obligatorily possessed noun to be used without a possessor. Known in most Uto-Aztecanist literature as *absolutive.* Examples are (6) and (8) below.
PSD (possessed): A morpheme on a head noun that registers a possessor (i.e. responds to its presence but does not index its grammatical categories; e.g. construct state in Semitic languages). Put differently, it marks a noun as having a dependent, i.e. as heading an NP (usually a possessive NP but occasionally other types). In some languages a PSD morpheme cooccurs with possessive indexation. Examples are in (6), (9) below. Some languages have pairs of possessed and non-possessed nouns formed with PSD and NPN from the same stem, as in several Uto-Aztecan languages:

(6)

**Table:** 

Pipil (pipi1250, Uto-Aztecan, Nicaragua; Campbell 1985: 38, 43)	
*siwa:-t*	*nu-siwa:-w*
woman-NPN	1sg-woman-PSD
‘woman’	‘my wife’

POSS: A non-classifying appositive word or morpheme. In some languages it may be a construct morpheme, registering a possessor on the head noun but not indexing its categories; in some it is in NP-second possession, associated with whichever noun is first in the phrase. In some languages it is a dedicated marker of possessive NPs, and in others it is more general. We gloss the possessive morphemes of single-classifier systems as POSS, as do some grammars. In grammars of some languages of Oceania (see Supplementary Material S3.5–3.6) it is called a linker, implying that it is juxtaposed between head and possessor and not syntactically or prosodically bound to either one (though not all sources discuss the constituency). Examples of POSS are (4) above, (51–52) below, or (glossed LINK in the source) (57) below.
Unspecified: A morpheme marking a noun as possessed by an unspecified or generic or unknown possessor. Usually it is part of a paradigm of person or person-number morphemes that index the possessor.
Owned non-possessible: a noun that is grammatically non-possessible but whose referent is owned. At issue in this paper is the various grammatical means languages have to allow a non-possessible noun to head a possessive phrase.7The fact that “owned non-possessible” is not an oxymoron shows that the conventional grammatical terms “possessable”, “non-possessible” (or its less frequent synonym “unpossessable”), “possessor”, “possessum”, etc. share the same stem as the non-technical verb possess but differ in meaning, denoting a grammatical (not semantic) relationship and not (only) ownership or the like.
A, O: Transitive subject and object prefixes in Ainu; the A series is also used for possessors.

A language with the full inventory of possessibility classes is Yanesha’ (yane1238, Arawak; Peru; Duff-Tripp 1997: 30–34):

Obligatorily possessed: Person-number prefix plus noun, no suffix:

(7)

**Table:** 

*po-ʔse* (3sg-brother) ‘his/her brother’

Obligatorily possessed nouns from which a non-possessed noun can be derived with the NPN suffix:

(8)

**Table:** 

*p-oñ*	*oñ-ets*
3sg-head	head-NPN
‘his/her head’	‘head, a head’

Optionally possessed: Animates (also some relational terms and terms for edibles), usually with PSD when possessed; inanimates without PSD.

(9)

**Table:** 

*ochec*	‘dog’
*p-ochc-ar* (3sg-dog-PSD)	‘his/her dog’
*cac*	‘fish’
*po-cac-ar* (3sg-fish-PSD)	‘his fish; his catch of fish’
*noñt´* 8	‘canoe’
*poʔ-noñt´* (3sg-canoe)	‘his/her canoe’

8 Duff-Tripp uses a tilde over all palatalized consonants. We write t´, etc. for letters we cannot place a tilde over.

Non-possessibles: chiefly *chesha’* ‘child’ and *huocchanesha’* ‘orphan’.

Yanesha’ also has the cross-linguistically infrequent type that can be called *alienably possessed inalienables*, where an inalienable noun is additionally owned. Yanesha’ uses two possessive prefixes in sequence, one referring to the inalienable possessor and the other to the possessor of the alienable (and its PSD suffix):

(10)

**Table:** 

*pa-ʔmeʔ*	*no-pa-ʔme-r*
3sg-egg	1sg-3sg-egg-PSD
‘its egg’ (i.e. the chicken’s)	‘my egg’ (chicken’s egg that I own)

Mora-Marín (2021) surveys possession in the Mayan family, where many languages have an array of types.

The syntactic structure of possessive NPs can be described in the same terms as that of clauses. Inalienable nouns have argument structure and valence: the possessor of such a noun is its argument. The possessor of an alienable noun is not an argument but an adjunct or modifier or similar non-argument. (For this point see Barker 2011; Koptjevskaja-Tamm 2001: 964; Lehmann 1985; Seiler 1983; Alexiadou 2003 gives an overview of the notion in X-bar syntax). The three types of possessibility are types of argument structure: the possessed noun can have, must have, or cannot have an argument (the possessor). The language-specific coding that possessed nouns take, and any syntactic constraints on it, are matters of valence. *Alienable* and *inalienable* are common terms for two main valence patterns, where *inalienable* usually labels obligatorily possessed or possessible nouns and *alienable* labels optionally possessibles or non-possessibles.

That the possessor of a non-possessible is not an argument of that noun is consistent with the morphosyntactic coding of many of them as adjuncts. It also explains why, in examples like (11), the same head noun can take a variety of appositive classifiers. The possessed noun does not require the possessor or dictate its form or the semantics of the relation (much as the verb does not dictate the form or semantic relation of an adjunct).

(11)

**Table:** 

Paamese (paam1238, Austronesian; Lynch et al. 2002: 42)	
*ani*	***ā*** *-k*
coconut	**CL**:FOOD-1sg
‘my coconut (which I intend to eat)’	
*ani*	***ema*** *-k*
coconut	**CL**:DRINK-1sg
‘my coconut (I intend to drink the juice)’	
*ani*	***esa*** *-k*
coconut	**CL**:PLANT-1sg
‘my coconut (growing on my land)’	
*ani*	***ona*** *-k*
coconut	**CL**:NEUTRAL-1sg
‘my coconut (e.g. to sit on)’	

## 3 Ainu possessive constructions

Ainu (moribund isolate, northern Japan) stands out among its Northeast Asian neighbors as atypical in being predominantly head-marking, exhibiting (morphologically non-complex but real) polysynthesis, and having a robust alienability opposition. Here we show that the formal implementation of alienability in Ainu is a type found among Circum-Pacific languages.

In Ainu, inalienables are obligatorily possessed. Only inalienables ‒ typically body parts (13a, 14a) and their semantic extensions, kinship terms, and relational nouns (13b, 14b) ‒ can be used in a proper possessive construction. (12) shows the template for the proper possessive NP construction. The possessum takes the possessed suffix (PSD) ***-*** *hV* or *-V(hV)* 9The value of the PSD vowel V partly depends on the last vowel of the root. that registers a possessor plus a person-marking prefix that indexes a possessor and is identical with the transitive subject marker or the object marker of relational nouns.

(12)

**Table:** 

[ [possessor: noun/(pronoun)]_N1_ [possessum: PERSON-noun-PSD]_N2_ ]_NP_

The possessor noun (N_1_) can easily be omitted without ambiguity as the possessed suffix signals its presence. It is most natural to omit the possessor as an N (or free pronoun) if it is a first or second person pronoun as in (14); in such cases it is overtly indexed on the possessum (PERSON). The third person is zero, so the default interpretation of a “noun-PSD” form standing alone would be ‘the …of him/her/it, his/her/its …’ as in (13a), which shows lexically possessed nouns in (13), and pronominally possessed nouns in (14). The possessed nouns in (13a) and (14a) are body parts; those in (13b) and (14b) are relational nouns.

(13a)

**Table:** 

*toan*	*nispa*	*Ø-sik-* ***i***
that	rich.man	3SG-eye-PSD
‘the eyes of that rich man’			(Satō 2008: 156)

(13b)

**Table:** 

*toan*	*cikuni*	*Ø* ***-*** *sam* ***-a***
that	tree	3SG-near-PSD
‘near that tree’			(Satō 1997: 152)

(14a)

**Table:** 

*(káni)*	***ku-*** *sik* ***-i***
1SG	1SG.A-eye-PSD
‘my eyes’		(Satō 2008: 156)

(14b)

**Table:** 

*(cókay)*	***un-*** *sam* 10
1PL.EXCL	1PL.EXCL.O-near
‘near us (him and me)’ (fieldwork (Bugaeva))	

10 PSD is not required on relational nouns with 1st/2nd person possessors.

Obligatorily possessed nouns almost never occur without possessive morphology. In response to elicitation of body part terms most speakers would provide the PSD forms of nouns, which are interpreted as having the 3rd person possessor, e.g. *sik* ***-ihi*** ‘the eye(s) of/his eyes’, *kisar* ***-a*** ‘the ear(s) of/his ear(s)’, *tek* ***-ehe*** ‘the hand(s) of/his hand(s)’ (fieldwork (Bugaeva); also Tamura 1993). Occasionally, some speakers choose the 1st person possessor encoding, i.e. ***ku-*** *yup* ***-i(hi)*** ‘my elder brother’ instead of just ‘brother’ (Krasheninnikov 1994 (1755): II-185, Tamura 1993: 9), but never just the bare forms (*yup* ‘elder brother’ or *sik* ‘eye’, cf. (15)).

Even in a few documented riddles, where there is no specified possessor, obligatorily possessed nouns do not occur in their bare forms, unlike other nouns in such riddles (cf. *mukur* ‘axe’, *tar* ‘rope’ in Tamura (1993: 14)). Instead, speakers mark the possessor with the fourth person (unspecified) transitive subject marker ***a-*** as in (15). (W and S are the two speakers in this recorded riddle). What is known as the fourth person in Ainu is basically an unspecified or indefinite possessor (‘someone/people in general’) but the form also has a range of other functions such as inclusive first person plural, second person honorific, and logophoric (including person of protagonist in folktales).

(15)

**Table:** 

W:	*sine*	*situ*	*u-ko-kor*	*wa*	*u-nukar*	*eramiskari*
	one	ridge	REC-with.APPL-have	and	REC-see	not.remember
	*p*	*hemanta*	*an?*			
	thing	what	exist.SG			
	‘What are the things that share one ridge and never see each other?’ (lit. ‘What are the things that have with each other one ridge (=nose) and don’t remember seeing each other?’)					

**Table:** 

S:	***a-*** *sik* ***-ihi***	*un*					
	4.A-eye-PSD	FIN					
	‘Eyes’ (lit. ‘people’s/ our eyes’)							(Tamura 1993: 16, 18)

In some cases, it is the derivational antipassive/absolutive marker *i-* that is used to encode an unspecified possessor on obligatorily possessed nouns when they are incorporated.11Obligatorily possessed nouns cannot be incorporated in their possessive forms (Satō 1992). When their possessors are coreferential with the subject they can be incorporated in just their non-possessive (bare) forms, but when non-coreferential they require the antipassive/absolutive prefix *i-* to be incorporated as in (16)–(18).

(16a)

**Table:** 

***i-*** *sapa-kar*	*usi*
ANTIP-head-make/do	place

‘a barber shop’, lit. ‘a place (where) (s)he does people’s heads’

(Tamura 1996: 247)

(16b)

**Table:** 

***i-*** *rekut-tuye*	
ANTIP-throat-cut	
‘(S)he cuts someone’s throat’	(Okuda 1999)

(17)

**Table:** 

***i-*** *y-ona-ne* 12
ANTIP-EP-father-COP
‘be a father’, lit. ‘be someone’s father’ (Nakagawa et al. 2020: K8303243UP.054)

12 The copula *ne* ‘be, become something/somebody’ is regarded as a special kind of transitive verb in Ainu grammar since it takes A-series transitive subject personal affixes but does not take O series affixes.

(18)

**Table:** 

***i-*** *sermak-us*
ANTIP-behind-attach.to
‘(for a god) to protect someone from behind’, lit. ‘…someone’s behind’
(Nakagawa et al. 2020: K8010291UP.521)

The antipassive, then, is functioning as an NPN marker, comparable to what are known as absolutives in Uto-Aztecanist analyses.

Some languages, for example Navajo (nava1243) (Young and Morgan (1987: 3)) and Slave (slav1253) (Rice 1989: 209) (both Athabaskan) or the Arawak (araw1281) (S3.4.2) and Tupian languages (S3.4.3),13Nanti (nant1250, Arawak) (S3.4.2) and Guarani (para1311, Tupian) (S3.4.3) mark the unspecified with the inclusive, which is reminiscent of the inclusive function of the fourth person in Ainu ((15) above). have an unspecified possessor as part of their person-number possessive paradigm. These end up doing semantic work similar to NPNs, but the NPN suffix lets the noun be used without possessive morphology while the unspecified possessor lets it be used with possessive morphology but without being specific about the possessor. As shown, Ainu has both the unspecified possessor in ***a-*** *sik-ihi* (4.A-eye-PSD) ‘someone’s eye(s), eye(s) of people in general’ in (15) and antipassive in ***i-*** *rekut-tuye* (ANTIP-throat-cut) ‘cut someone’s throat’ in (16b). The latter is reserved for O-incorporation of obligatorily possessed nouns with non-coreferential possessors.

The semantically optionally owned nouns of Ainu are usually termed *alienable*. Typical alienable nouns ‒ animals, fish, plants, utensils, and some kinship terms ‒ are non-possessible in Ainu: they cannot take the regular possessive morphology but instead use the verb ***kor*** ‘have’ which functions as the predicate of a (gapped) pre-head relative clause (19a) that has the possessor as the subject of the relativized verb and the possessum as the head noun (repeated from (3)).

(19a)

**Table:** 

[—	*ku* ***-kor***]	*seta*
O	1SG.A-have	dog
‘my dog’, lit. ‘the dog (that) I have/had’.		

The base construction is a finite clause as in (19b):

(19b)

**Table:** 

*poro*	*seta*	*ku-kor*
big	dog	1SG.A-have

‘I had a big dog.’ (Nakagawa et al. 2020: K7708242UP.163)

Though grammatically the *kor* construction is possible with all non-possessible nouns, e.g. *ku-* ***kor*** *cup* [1SG.A-have sun] ‘my sun’ (Tamura 2001[1964]: 130), only a few nouns are actually attested in the *kor* construction in the texts used for this study.14The corpus consulted includes Nakagawa et al. (2020), currently 59,002 Ainu words, and a huge bulk of folklore texts (Ainu Phonetic Material (1–6)) searchable through Tamura (1991). Tamura’s lexicon with examples (1991) contains 57 such nouns, which can be grouped as follows:

(a) People: 24 items (*aynu* ‘human, man’, *hapo* ‘mother’, *nispa* ‘rich man’ etc.);
(b) Place/environment: 11 items (*kotan* ‘village’, *mosir* ‘land, country’, *pet* ‘river’);
(c) Food/drink: 7 items (*aep* ‘food’, *cep* ‘fish’, *pirkep* ‘rice’ etc.);
(d) Tools/utensils/ritual object: 7 items (*emus* ‘sword’, *ikor* ‘treasures’ etc.);
(e) Domestic animals: 1 item (*coni* ‘small horse’); also *seta* ‘dog’ and *pewrep* ‘bear cub’ in other sources;
(f) Human attributes: 7 items (*isoytak* ‘story’, *itak* ‘language’, *rametok* ‘bravery’).

The high frequency of (a) human nouns might be due to the extended use of the *kor* construction to express a feeling of affection (Tamura 1988/2000: 88), e.g. ‘my dear (to a young girl)’, and the same might be true for (f) ‘human attributes’.

(20)

**Table:** 

*a-* ***kor***	*pon*	*menoko*	*a-e-yaykatekar*
4.A-have	young	woman	4.A-APPL-love
‘I loved my young girl.’ (Tamura 1988/2000: 60)			

Food, house, village, household-related items, and domesticated animals are most typically attested with *kor*, while wild animals and plants, which do not belong to anyone, are absent from the list. Exceptions are ‘fish’, because villages owned sections of rivers where they could harvest fish, and ‘bear cub’, because bear cubs were ritually adopted and raised in the village.

There is good evidence that Ainu formerly had a larger set of verbs with classificatory functions. In oral literature, which is fairly old and of high-register style, a number of other transitive verbs have been attested to perform the same possessive/ownership function specifying and classifying the type of possessive relation. Some of these verbs are fixed high-frequency expressions as in (21), (25), and (26).

(21)

**Table:** 

*a-* ***un***	*cise*	*ta*	*hosipi-an*	*na*
4.A-attach.to/belong	house	LOC	return-4.S	FIN
‘I returned to my house’, lit. ‘the house (where) I belong’				
(Nakagawa 1995: 71)

(22)

**Table:** 

*a-* ***mut***	*emusi*	*notak*	*kas-i-ke*	*e-ray*	
4.A-wear	sword	edge.of. sword	top-PSD-PSD	at.APPL-die	

**Table:** 

*kuni*	*p*	*e-ne*	*ruwe*	*ne*	*na.*
should	person	2SG.A-COP	INF.EV	COP	FIN
‘You are a person (who) should die on top of the edge of my sword.’					
lit. ‘the sword (which) I wear’ (Nakagawa 2001: 99)

(23)

**Table:** 

*an-* ***reska***	*pewre-p*
4.A**-**raise	young-thing (=a young bear cub)
‘my raised bear-cub’,^15^ lit. ‘the bear cub (which) I raised’	
(Sunazawa 1983: 24)	

15 The bear cub was ritually adopted, raised in a cage, and killed at a bear festival, a ceremony sending off the spirit of the bear to the land of gods.

(24)

**Table:** 

*a-* ***resu*** 16	*cape*
4.A**-**raise	cat
‘my cat’, (lit. ‘my raised cat’)		(Nakagawa et al. 2020: K8010311UP.026)

16 *resu* ‘raise’ (Southwestern Ainu) and *reska* ‘raise’ (Northeastern Ainu) in (23) are dialectal variants of the same word.

(25)

**Table:** 

*e-* ***an-te***	*hoku*
2SG.A-exist.SG-CAUS	husband
‘your husband’, lit. ‘husband (who) you made stay (with yourself)’	
(Nakagawa et al. 2020: K7807152KY.030)

(26)

**Table:** 

*a-* ***hekote***	*nispa*/ *katkemat*/ *ekasi*
4.A-be.married.to	rich.man/ married.woman/ grandfather
‘my husband/wife/grandfather’, lit. ‘husband/wife/grandfather (who) I am married to’ (Nakagawa et al. 2020: K7708242UP.303, K7803232UP.075, K7803233UP.096)

(27)

**Table:** 

*a* ***-mi***	*kosonde*	*a* ***-*** *mi*	*ruwe*	*ne*
4.A-wear	Japanese.kimono	4.A-wear	INF.EV	COP
‘I put my Japanese kimono on.’					(Kubodera 1977: 208)

Example (27) shows the difference between ordinary verb (the second *mi*) and classifier (the first *mi*). Also, it suggests that the starting point of non-possessibility might be unfamiliarity: *kosonde* ‘short-sleeved kimono’ is a Japanese loanword (and cf. the Tzotzil (tzot1259) example with ‘bus’ in §5.2).

Further evidence for the possible existence of a larger set of classifier verbs is compound nouns that originate in verbs with their phraseologized objects (unspecified or generic nouns) as in (28).

(28)

**Table:** 

*c-* ***e-*** *p* (1PL.EXCL.A-eat-thing) ‘fish’, lit. ‘the thing we (EXCL) eat’,
*ci-* ***ronnu*** *-p* (1PL.EXCL.A-kill.PL-thing) ‘fox’, lit. ‘the thing (that) we (EXCL) kill’
*ci-* ***koyki-*** *p* (1PL.EXCL.A-catch-thing) ‘game’, lit. ‘the thing (that) we (EXCL) catch’
*ci-* ***ramante-*** *p* (1PL.EXCL.A-hunt-thing) ‘bear’, lit. ‘the thing (that) we (EXCL) hunt’
*a-* ***e-*** *p* (4.A-eat-thing) ‘food’, lit. ‘the thing (that) we (INCL) eat’,
*a-* ***kor-pa-*** *p* (4.A-have-PL-thing) ‘belongings’, lit. ‘the thing (that) we (INCL) have’
*a-* ***mi-*** *p* (4.A-wear-thing) ‘clothing’, lit. ‘the thing (that) we (INCL) wear’
*c-* ***ani*** *-ku* (1PL.EXCL.A-hold-bow) ‘bow, hunting bow’, lit. ‘bow (that) we hold (when hunting)’
*c-* ***ama*** *-ku* (1PL.EXCL.A-put/set-bow ‘bow trap’, lit. ‘bow (that) we set up (as a trap)’

We hypothesize that an older set of classificatory verbs in Ainu included items specifying how animals were hunted and raised, food preparation types, clothing, houses and objects by function. Historically, former more specific verbs must have been replaced with *kor* ‘have’ and erstwhile multiple alienable classes (with different verbs) reduced to just to one as in (29b).

(29a)

**Table:** 

*a-* ***ma***	*a-* ***e-*** *p*
4.A-grill	4.A-eat-thing
‘my grilled food’ lit. ‘the thing(s) (that) I grill’	
(Nakagawa et al. 2020: K7803231UP.033)

(29b)

**Table:** 

*a-* ***kor***	*a-* ***e-*** *p*
4.A-have	4.A-eat-thing
‘my food’ lit. ‘the thing (that) I have (that) I eat’, ‘the edible(s) that I have’	
(Tamura 1985: 6)	

We reconstruct the following diachronic progression for the development of verbal possessive classifiers in Ainu:

- Old set of verbal possessive classifiers (as in sporadically retained verbs (21)–(27), (29a) and numerous frozen nouns (28)) >
- General possessive verbal classifier ***kor*** ‘have’ (19a), (29b)

Although the possessive ***kor*** construction is fully conventionalized in Ainu, syntactically it still retains most of the features of a normal relative clause. For example, the relative clause predicate ***kor*** ‘have’ can optionally be marked for plurality (30), perfectivity (31), or mirativity and can take adverbial modifiers.

(30)

**Table:** 

[*ona-ha*	__*kor*	***pa***]	*p*	*opitta*
father-PSD	have	PL	thing	all
‘All of the thing**s** (that) his father owned’					(Tamura 1985: 42)

(31)

**Table:** 

[—*a-kor*	***a***]	*kotan*	*a-ko-hosipi*	*ruwe* *ne.*	
4.A-have	PRF.SG	village	4.A-to.APPL-return	INF.EV	COP
‘I returned to my **former** village.’ (Tamura 1989: 64)					

Relative clause stacking is easily allowed, i.e. we can have two possessive *kor* constructions (32) or the possessive *kor* and other relative clause in any order (33) (note that there is no separate class of adjectives in Ainu, so *pirka* ‘good’ and *pon* ‘small’ below are relative clause intransitive predicates).

(32)

**Table:** 

[[— ***kor***]	*sinrit*	__ ***kor***]	*pe*
have	ancestors	have	thing/person
‘Their ancestors’ things’ (Nakagawa et al. 2020: K7803233UP.101)

(33a)

**Table:** 

[*pirka*] [__ ***kor***]	*pe*
good have	thing
‘His good things’ (Nakagawa et al. 2020: K7803233UP.379)

(33b)

**Table:** 

[— *a-* ***kor***]	[*pon*]	*oni*
4.A-have	small	demon
‘My little demon’ (Nakagawa et al. 2020: K7708241UP.018)		

Unsurprisingly, inalienable and alienable possessive constructions can combine into larger syntactic structures.

(34)

**Table:** 

[— *a-* ***kor***]	*pet*	*hontom* ***-o***	*ta*
4.A-have	river	half.way-PSD	LOC
‘At the middle (reaches) of our river’ lit. ‘halfway of the river (that) we have’ (Nakagawa et al. 2020: K7803233UP.172)			

Yet, there are some signs of grammaticalization of the *kor* possessive construction with regard to phonology. If the possessor (the relative clause subject) is third person, *kor* ‘have’ lacks overt person marking and (unlike other relatives or other phrases in general) tends to constitute a single phonological unit with the following possessum (the head noun) with a pause (//) after the subject of the relative clause.

(35)

**Table:** 

*Anna*	*katkemat*	//	***kor*** *-ninkari,*	*Ito*	//	***kor*** *-ninkari*
Anna	married.woman		have-earrings	Ito		have-earrings
‘Mrs. Anna’s earrings and Ito’s earrings’^17^						

17 From AB’s elicitation.

This also shows that an erstwhile dependent-marking *kor* construction is in the process of turning into a head-marking construction: the *kor* verb is cliticizing to the head noun, consistent with the scenario adduced in Nichols (1986: 84–85).

Using relative clause structures for complex NPs here is a natural choice for Ainu since the language lacks resources like adjectives, adpositions, or genitive case marking that are recruited in various other languages for expressing possession (Heine 1997). Also, Ainu does not easily form non-minimal NPs, and most of its attributive modifying constructions (locative, propriative, privative, etc.) also obligatorily use verb-based relative clauses similar to alienable possession.

(36)

**Table:** 

[—*sat-cep*	***o***]	*pu*
dry-fish	enter/have	storehouse
‘a storehouse with dry fish’, lit. ‘dry fish-entered storehouse’		
(Tamura 1996: 547)

(37)

**Table:** 

[—*Iskar* ***-un***]	*kur*
Ishikari-live.at	man
‘a man from Ishikari’, lit. ‘an [Ishikari-living] man’ (Nakagawa 1995: 33)	

## 4 Typological properties of appositive possessive systems

The analytic framework for our typology of appositive possessive constructions is contained in the definitions in §2.2 above. Here we discuss some findings from our survey which inform our comparison of Ainu to the other languages with appositive possessives.

### 4.1 Basics

In languages with noun appositives, the appositive is generally obligatorily possessed and often described as an inalienable noun in the language. Verbal appositives generally have person-number indexation referring to the possessor; often they are finite, and where nonfinite they are usually inflectional forms of the verbs rather than derivatives. Thus they are lexically and grammatically verbs.

### 4.2 Open and closed classifier systems

#### 4.2.1 Number of classifiers

Appositive possessive subsystems can be characterized for convenience as small or large. This section discusses first the number of classifiers, then the number of non-possessible nouns. Details are in Supplementary Material 3.

For appositives, examples of small systems are Paamese, illustrated in (11) above, with four classifiers; Washo (wash1253, isolate, Nevada-California, US; Jacobsen 1964), with only two appositives ‘pet’ and ‘plant’; or Wichi Lhomtes (Nercesian 2014), with two classifiers, one for animals illustrated in (5) above and one for inanimates:

(38)

**Table:** 

*n’ -ka-husan*
1sg-CL-axe
‘my axe’ (Nercesian 2014: 168)

The ultimate small system is a one-appositive one, found in contemporary Macushi (§2.2 (4) above). That single appositive is the noun ‘animal’ and used only with animal names, so it can be called a classifier; this is then a one-classifier system. (Carlson and Payne 1989: 103 note that an older source on Macushi (Williams 1932) also has a classifier for food). Another one-classifier system is Yurakare (§S3.4.6.3). Others are found in languages of the Timor-Alor-Pantar family (Papuan) of eastern Indonesia:

(39)

**Table:** 

Bunaq (east2519, Timor-Alor-Pantar, Indonesia; Donohue and Schapper 2008: 322, citing Steinhauer 1977, 1991)		
*n-up*	*ni-e*	*zap*
1-tongue	1-POSS	dog
‘my tongue’ (inalienable)	‘my dog’ (alienable)	

These can be said to classify for alienability, where kin terms and/or body parts, and usually a few other nouns, are inalienable. Small systems are usually closed systems.

Large systems include those of Iaai (New Caledonia; Austronesian: Oceanic), for which Lynch et al. (2002: 782–783) give 21 classifiers selected from the larger list of Ozanne-Rivierre (1976); or Panare (enap1235, Pemong-Panare subbranch of Venezuelan Cariban), for which Payne and Payne (2013: 81–82) list 20. Large systems are usually open, and they often contain items with varied derivational sources: typically, some are derivationally or etymologically related to nouns, some to verbs, and some have no known source.

Verbal appositive classifier systems are often small: examples are the one-classifier systems of Ainu (above), Tutelo (tute1247, Siouan; Virginia, US; Oliverio 1996), where the non-possessibles use either a PSD prefix or a nominalized form of the verb ‘belong to’; and Seri (seri1257, isolate; Mexico), which uses the verb ‘own’:

(40)

**Table:** 

Seri (isolate, New Mexico; Marlett 1981)			
*simalón kiʔ*	*tro:ki*	*ya:*	*kiʔ*
Cimalon the	car	3.NMZ.own	the
‘Cimalon’s car’ (p. 69; extracted from clause)

(41)

**Table:** 

*kanóatax*	*ʔi-o-ya:t*	*koi*
boats	1-NMZ-own	the
‘our boats’ (p. 70; extracted from clause)		

A large verbal system is that of Cahuilla (Uto-Aztecan, Arizona; Seiler 1977, 1995), which has two animal classifiers, one for ordinary domestic animals and pets and one for totem animals; a general inanimate classifier; four for food plants, the choice depending on traditional relations of ownership and usufruct; and several for prepared foods, based on how they are prepared. All are nominalized forms of verbs or flexible noun-verbs, except for the general animal classifier, which is a bare form of the verb:

(42)

a.

**Table:** 

*né-ʔaš*	*ʔáwal*
1sg-own	dog
‘my dog’ (Seiler 1995: 219)

b.

**Table:** 

*pe-n-áš-qal*	
3sg.O-1sg-own-DUR	
‘I own it (as a pet)’	(Seiler 1977: 305, 1995: 219)

An example of a Cahuilla classifier based on traditional ownership is the one for stands of naturally growing plants or the places where they grow: mesquite, oak/acorn, pinyon, chia. Such places were assigned to clan lineages; “[t]he members of the lineage had a legal claim and were allowed to harvest these places when the appropriate time came” (Seiler 1995: 301).

(43)

a.

**Table:** 

*ne-kiʔiw-ʔa*	*méñikiš*
1sg-wait-NZ	mesquite.beans
‘my mesquite beans’ (1995: 221; see also 1977: 301)

b.

**Table:** 

*pe-n-kíʔiw-qal*
3sg-1sg-wait-DUR
‘I am waiting for it’

For the other classifiers of Cahuilla see Supplementary Material S3.2.2. The Cahuilla system is presumably open, but closely related and nearby Cupeño (cupe1243) (Hill 2014) has only the animate and inanimate classifiers, both cognate to the Cahuilla analogs. Given the transparency of the Cahuilla system, its uniqueness within Uto-Aztecan, and the apparent absence of any fossilized former classifiers in Cupeño, the Cahuilla system is probably a recent expansion.

#### 4.2.2 Number of non-possessibles

Turning now to the set of non-possessible nouns, it is often open, though it may be a restricted open set such as all animal names. The obligatorily possessed nouns are a smaller, usually enumerable system, though they too could be said to be open but restricted in languages where all kin terms, or all body parts, are obligatorily possessed. In languages like Yanesha’ (Duff-Tripp 1997) or Washo (Jacobsen 1964) the non-possessibles are a closed set and the optionally possessibles presumably open. In Biloxi (bilo1248, Siouan; Mississippi, US; Einaudi 1976) there are three classes: obligatorily possessed (kin terms, body parts), optionally possessed (a few nouns denoting intimate possessions such as ‘house’ and items of clothing, which are marked the same way as inalienables when owned), and all other nouns, which cannot be inflected for person. How ownership of these non-possessibles was expressed is unknown. In languages with a small set of non-possessible classifiers consisting primarily or exclusively of ‘animal’ and ‘inanimate’, the nouns used with ‘inanimate’ are presumably open and ‘animal’ open but restricted.

The ultimate degree of openness and the ultimate largest set is languages where all nouns are non-possessible. This description fits languages of the Caddoan family (Wichita [wich1260] in our sample: Kansas to northern Texas; Rood 1976: 143–150)), where apparently no nouns can take person indexation (or any other inflection; nouns are a non-inflecting part of speech). The only possessive construction in Wichita is relativization. (POSS here is a verbal morpheme, possibly a construct marker, indicating possession: see Supplementary Material S3.2.6).

(44)

a.

**Table:** 

*niye:s* *niya:wéʔekih*
{niy-uR-waʔ-iki}
child PCP.indef:S-POSS-DISTRIBUTIVE-be.PLURAL
‘their children’ (lit. ‘child they-are-theirs’)^18^ (Rood 1976: 144; extracted from clause)

18 Here and below, translations marked "lit." are our suggested literal (or more nearly literal) renditions.

b.

**Table:** 

*natí:weʔesikih*
{nat-u’r-weʔes-iki}
PCP.1S-POSS-dog-be.PLURAL
‘my dogs’ (145; extracted from clause)

Kwaza (kwaz1243, isolate, northwestern Brazil; van der Voort 2004) makes no alienability distinctions but does use classifiers (numbering about 150) in possession: the possessor is in the genitive case, followed by either a nominalizer or a classifier. Van der Voort analyzes the nominalizer as a neutral classifier. In the following examples *-hỹ* is the nominalizer/neutral classifier and *-xy* is the classifier for such things as houses.

(45a)

**Table:** 

*‘si-dy-hỹ*	*a’xy*
1sg-GEN-NMZ	house
‘my house’ (nominalized)

(45b)

**Table:** 

*Tawi’wi-dy-hỹ*	*a’xy*
T.-GEN-NMZ	house
‘Tavivi’s house’ (nominalized)

(46a)

**Table:** 

*(a’xy)*	*si-dy-xy*
house	1sg-GEN-CL:house
‘my house’ (classifier)

(46b)

**Table:** 

*Tawi’wi-dy-xy*
T.-GEN-CL:house
‘Tavivi’s house’ (classifier) (van der Voort 2004: 130–131)

Since there is no alienability distinction, and since the possessor word is nominalized, Kwaza can be described as a language with exclusively appositive possession using classifiers.

For some languages there is a class of non-possessibles but their treatment when owned is not appositive. In most Algonquian languages19We thank Richard A. Rhodes for consultation on Algonquian. there is a small class of non-possessibles, which have possessible synonyms, e.g. Menomini (meno1252, Bloomfield 1962: 42):

(47)

**Table:** 

*anè:m*	‘dog’	(non-possessible)
*ne-t-i:hsèh*	‘my dog’	(possessible)
*1sg-EP-dog*	*(EP = epenthetic)*	

Uniquely in Algonquian, Arapaho (arap1274) uses the possessible word for ‘horse’ to mean ‘pet’, which can be used appositively (this is much like the Numic (numi1242, Uto-Aztecan) languages discussed in S3.2.2 and is an evident contact feature as Arapaho borders on the Numic language Shoshone (shos1248)):

**Table:** 

*wóxhoox*	‘horse’ (non-possessible)
*nó-toníhi’*	‘my horse’ (possessible)
*nó-toníhi’*	*beníixóxko’ó’*
1sg-pet	goat
‘my goat, my pet goat’ (Cowell and Moss 2008: 67, 68)	

In most Algonquian languages, inanimate nouns (a formal gender and verb classification class) cannot be possessors. A relative construction is used instead, e.g. Arapaho in (48). The relative construction does not define a class of non-possible nouns. Rather, here it is the possessor that determines the construction type: inanimates cannot control possessor indexation, and relativization is used instead. The construction shows that, in general, verbal possessive inflection is an alternative available for when nominal possessive inflection is precluded.

(48)

**Table:** 

Animate possessor:	*hi’óó3*
	3sg-leg
	‘his/her leg’
Inanimate possessor:	*hí-íooe-éihi-:noo-’*
	3.leg-AI.PASS-II-ØS
	‘its leg’, lit. ‘it has a leg, the leg it has’^20^
	(Cowell and Moss 2008: 64)

20 AI = animate intransitive, the Algonquianist term for a formal class of verbs; II = intransitive inanimate, another class. In this class, relative and finite verbs are identical.

### 4.3 Semantics of appositive words

Languages with appositive classifiers can have numeral or other classifiers as well. Most languages of the Micronesian branch of Oceanic (Austronesian) have numeral classifiers as well as appositive possessive classifiers, and some Amazonian languages also. The systems of classifiers and their contexts of use are discrete. Only in Motuna (siwa1245, Bougainville, Papua New Guinea, Oceania; South Bougainville family; Onishi 2012: 244–245) have we observed a single classifier system that serves as both appositive possessive classifiers and numeral classifiers.

Where they coexist, numeral and possessive classifiers seem to have recurrent semantic differences. For the Oceanic languages, Lynch et al. (2002: 42) observe that “the numeral classifiers are generally based on physical form, while the possessive classifiers are generally based on function”. Our impression is that possessive classifiers often have to do with function, relation, activity, or how possession was acquired. (See Supplementary Material S3.2.2 for this motivation of classification in Cahuilla.) This is consistent with the fact that, where derivational or etymological information is available, a number of the possessive classifiers appear to be derived from or etymologically related to verbs; e.g. in Iaai (iaai1238) (Supplementary Material S3.5.1) roughly equal numbers of appositives are related to nouns, related to verbs, and of unknown origin. Verbal origins are especially clear in the larger systems, but in the two-classifier system of Cupeño and the cognate items in Cahuilla (§4.2.1) both classifiers are verbal (overtly nominalized in Cahuilla but not in Cupeño). The sole classifier of Ainu is a verb.

There appear to be few clear semantic generalizations to be made about what words are likely to become appositive classifiers and what kinds of semantics will be salient in the classifier system. Food and drink are ubiquitous among the possessive classifiers in the Pacific; in the Americas, animals and food plants. In larger systems, clothing, implements, and fire (including materials, tools, and other things connected with making and using fire) seem to be frequent.

The lack of semantic generalizations in appositives contrasts with the consistent generalizations that characterize non-possessibles. These almost always include natural kinds (especially animals and plants) and natural phenomena.

### 4.4 Lexical specification of appositives and valence

In languages with appositive possessives, are the appositives lexically specified as classifiers? as classifiers used with or required by particular nouns? are nouns lexically specified as requiring, allowing, or not allowing appositives? as requiring particular appositives? The answer to most of these is *No*. Possessive classification is the NP analog to valence, and just as it is predicates that require particular arguments and assign particular cases to those arguments, and not the nouns and pronouns that demand to be in argument roles or bear particular cases, so it is with possession. The one *Yes* answer is that the appositive words appear to be lexically specified as classifiers. In large systems, the classifiers that have no known derivational or etymological connections to other words are clearly only classifiers now. In many systems, some or all of the classifiers can also function as independent nouns (or verbs); this means that their lexical class, and possibly even their part of speech, is flexible or underspecified.

Otherwise, the appositives have minimal lexical specification. They do not appear to be specified as used with particular nouns; in larger systems there is a good deal of flexibility. Limits are set by semantics: in a minimal system with two classifiers, for animates and inanimates (like that of Cupeño, Supplementary Material S3.2.2), there will of course be no overlap, but this is not a matter of strict government by either the appositive or the head noun.

It is the head nouns that are lexically specified as having argument structure: as requiring, permitting, or not allowing possessors. It may be that they are also specified as allowing or not allowing the valence-changing NPN and PSD derivations, but we have not seen this covered in descriptions. (It is suggested in Duff-Tripp’s description of Yanesha’, (9) above: most of the nouns called “optionally possessed” are obligatorily possessed inalienables that can take an NPN suffix to derive an alienable noun, i.e. one that can be used without possessive morphology).

Dictionaries, then, need to indicate whether a noun is possessible, obligatorily possessed, or non-possessible, and classifiers need to be identified as such. Classifiers are generally identified in dictionaries, but in our experience the full set of optional/obligatory/prohibited possession is not always identified.

### 4.5 Tight vs. loose bond

Within an appositive possessive NP, it appears that the appositive word is more tightly or more loosely connected to the head noun, depending on the language. Several descriptions note that the possessive constructions can be discontinuous, with the classifier detached from the possessed noun, while in some languages they form a tighter phrase with strict order and no detachability (e.g. Meira 1999 for Tiriyó [Cariban]). Those describing the looser appositive syntax generally describe the construction as something other than an NP and prefer the term “generic noun” to “classifier”. The tighter phrase appears to be what is called *close apposition* in English descriptive grammar (e.g. Acuña-Fariña 2016; Heringa 2012; Huddleston and Pullum 2002: Ch. 15, with different terms; a frequently used example is *the poet Burns*). Close possessive apposition is generally taken to be a two-nominal NP where the relation between the nominals is neither coordination nor modification.

### 4.6 Indexation in possessive classification

In the languages we have surveyed, the obligatorily possessed nouns and the possessive classifiers index the possessor in person (or, more commonly, person-number). Head-marked possessive morphology appears never to index gender alone. This generalization holds for all kinds of possessive constructions and not just for appositive ones. When there is gender indexation in possessive constructions it is dependent-marked, the possessor indexing the head, e.g. Bantu languages (Maho 1999: 102–103), where the possessor is preceded by a possessive word which indexes the class (gender) of the head noun.

(49)

**Table:** 

Kiswahili (swah1253, Bantu; Maho 1999: 102 citing Perrott 1957: 27)			
*kitabu changu*			
{*ki*	*tabu*	*ch+a*	*angu}*
NP.7	book	PC.7	my
‘my book’ (NP = noun prefix, PC = possessive concord [i.e. index])			

The generalization about head-marked indexation appears to be categorical, but it could be that head-marked possessive gender indexation is just very rare. Gender indexation is rare in Circum-Pacific languages, and the probability is very small that the rare indexation type will occur in the area where the category involved is rare.

These observations about gender and head/dependent marking apply only to possessive phrases. Verbs can index gender, either as a third person category (as in Bantu languages) or with any person reference (as in a number of Nakh-Daghestanian languages).

### 4.7 Verbal appositives

The verbal appositives described above for Ainu (§3), Tutelo (§4.2.1), and Seri (§4.2.1) are small systems of one or two verbs, usually meaning ‘own’ or ‘have’. The large system of Cahuilla consists of verbs describing how one acquires or prepares things, chiefly foods, but there are also two fairly general verbs for animates (the verb means ‘own’) and inanimates (the verb means ‘do’). The cognates to these two make up the entire system in closely related Cupeño, and Luiseño from the same subbranch has the animate one as its only classifier.

Itonama (iton1250, isolate, Bolivia; Crevels 2006, 2009) uses verbal appositives in the form of relativizing morphology on alienably possessed nouns. Alienables take the regular person-number prefixes followed by the relativizer/nominalizer *mi- / ni-* . Forms of *uku* ‘house’ (2009: 250):

(50)

**Table:** 

*as - mi - ku*	*dih - ni - ku*	*sih - ni - ku*
1sg-REL-house	INCL-REL-house	1pl-REL-house
‘my house’	‘our-INCL house’	‘our-EXCL house’

The pattern is reminiscent of Ainu except that there is no verb root involved in the possession. These examples might be paraphrased ‘house which (is) mine’, etc. and regarded as relativized counterparts of the verbless predicate nominals discussed in §4.8 below.

Modern Urarina (urar1246, isolate, northern Peru lowlands; Olawsky 2006), under Spanish influence, has lost aspects of its former possessibility opposition, but an originally verbal appositive system is still evident in traditional language. Inalienables were obligatorily possessed, with person prefixes. Alienables were non-possessible and, when owned, used an appositive construction using a nominalized form *raj* of the verb ‘receive’ preceded by a juxtaposed noun or independent pronoun.

(51)

**Table:** 

*akaïrï*	*raj*	*batia*
3pl	POSS	pot
‘their pots’		(336)

(52)

**Table:** 

*kača*	*raj*	*lureri*
man	POSS	house
‘the man’s house’		(335)

The appositive word is now optional, or can take the possessive clitic of inalienables.

Two unrelated, geographically distant non-Austronesian (Papuan) languages of the Pacific have verbal appositives. In both languages inalienables use head-marked possession and alienables use a verb ‘possess’ or ‘belong to’ as appositive. (For fuller descriptions and examples see Supplementary Material S3.6).

(53)

**Table:** 

Mpur (mpur1239, isolate, Bird’s Head, New Guinea; Odé 2002: 62)	
*n-tar*	*jan*
1sg-possess	house
‘my house’

(54)

**Table:** 

Sulka (sulk1246, isolate, New Britain, PNG; Tharp 1996: 80–81)			
*a-kom*	*to*	*mkor*	*e-Pruo*
SG-knife	DEM	belong:to	Pruo
‘Pruo’s knife’	(117)		

‘Have/own/possess’ or ‘belong to’ figure cross-linguistically in adnominal possessive constructions of all kinds but normally it has nothing to do with non-possessibility. In Maltese and some Arabic dialects, the use of the genitive marker *ta* originating in the verb ‘to belong’ is limited to the alienable construction, e. g. ‘my chair’ comes from ‘chair belonging-my’ (Koptjevskaja-Tamm 1996: 247). However, in many Creole languages, there is no such distinction so ‘belong’ can occur in nominal possessive constructions with any semantics such as *buk* ***bilong*** *mi* ‘my book’ or *brata* ***blong*** *yu* ‘your brother’ in Tok Pisin (tokp1240) and other English-based Creoles (Bislama bisl1239), and the same for *punya*, *pung* ‘belong’ in Malay-based Creoles21We thank Yury Lander for noting that similar constructions occur in some non-creole Malay varieties as well. (Singapore Bazaar Malay (baba1267), Ambon Malay (ambo1250)) and *tá* ‘belong’ in Arabic-based Creoles (Kinubi (nubi1253), Juba Arabic (suda1237)) (Michaelis et al. 2013). We suppose that if a language uses a verb for adnominal possession and has only one such verb it is likely that that verb will be one meaning ‘have/own/possess’ or ‘belong to’.

### 4.8 Appositive and inalienable NPs are predicative

Though we have not done a formal survey, we have noted that languages capable of inflecting nouns for person often also use those person-inflected nouns as predicate nominals in verbless constructions. Examples include (55) as well as (S19–20) in Supplementary Material S3.2.2 from Tümpisa Shoshone (pana1305) or (S124–126) in Supplementary Material S3.4.3 from Kamaiurá (kama1373). This applies to inalienably possessed nouns and to the appositive noun in an appositive possessive construction (recall that appositive nouns are inalienables). Velasquez-Castillo (1996: 69–85) gives a native speaker’s perspective on the construction in Guaraní (see examples in Supplementary Material S3.4.3).

(55)

**Table:** 

*wararwijaw-a*	*je =r-eymap*
puppy-N	1sg=LINK-CL:pet
‘the puppy is my pet’	

It is unsurprising that, in languages with neutralization of the noun-verb distinction in predicate nominals (for this analysis see Beck 2013), the flexible noun-verb indexes the subject and functions as predicate. What is noteworthy in our examples is that the person inflection indexes the possessor, not the subject (see again (55) above); and the noun-verb neutralization applies only to inalienables and not to predicate nominals in general. Inalienables have an argument slot, as discussed above, but that argument is the possessor and not the subject. Cross-linguistic work, probably corpus-based, will be needed to determine whether this is the effect of the person inflection or of the inalienability. We propose this hypothesis to experts on relevant languages: Person inflection is prone to carry, or even impose, predicative semantics.

## 5 Distribution

### 5.1 Geography

**Figure 1:**
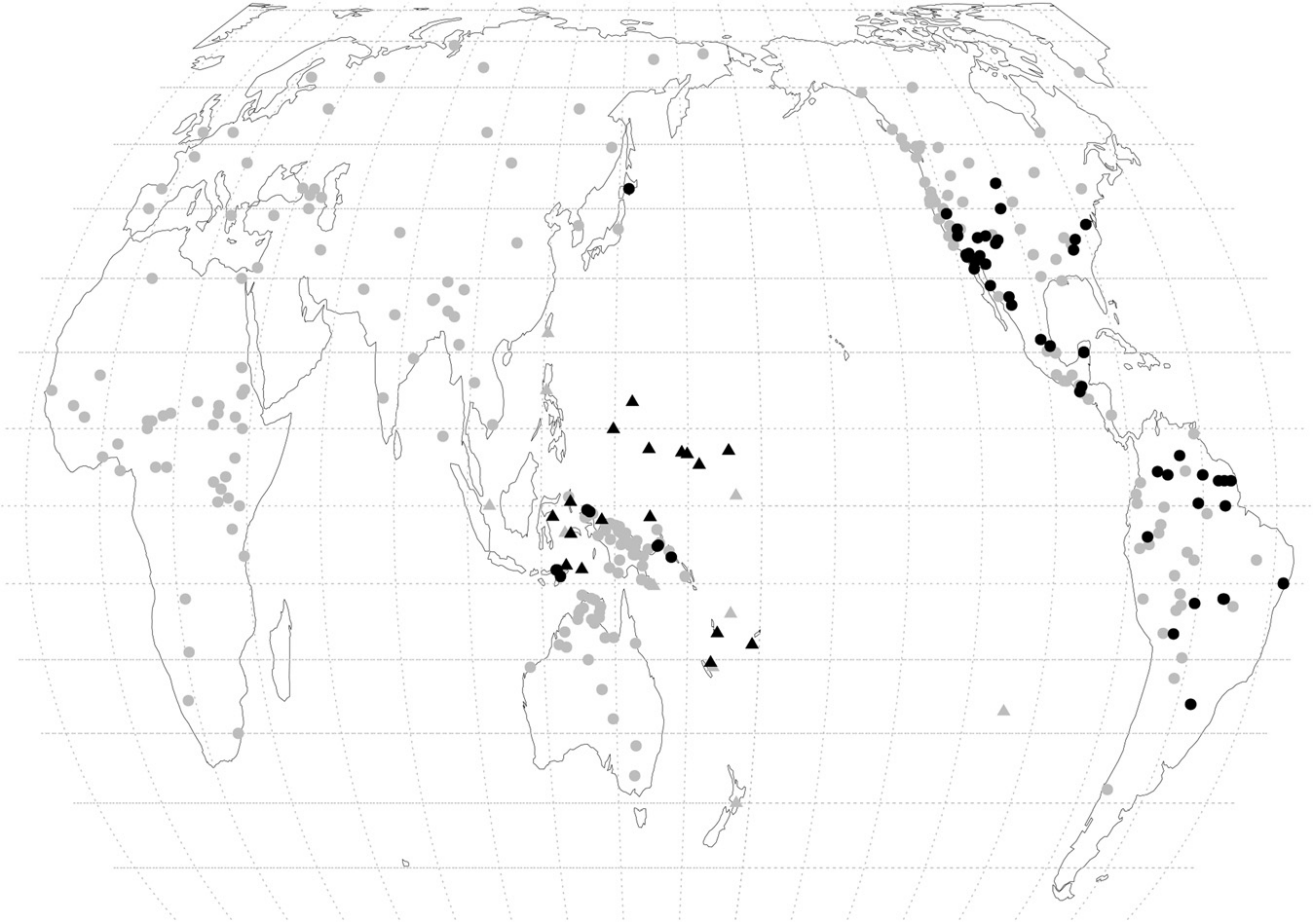
Languages with (black) vs. without (gray) appositive possessive constructions. Triangles represent Austronesian languages, which make up the majority of the cases with appositive possessives in the west. (Base map: Equal Earth projection, Šavrič et al. 2019).

Figure 1 combines the results of the survey with the data in the *World Atlas of Language Structures* (Nichols and Bickel 2005a).

There are two main clusters of languages with appositives: Oceania and the Americas, excluding the northern half of North America.

The Oceania cluster comprises six non-Austronesian families and four separate innovations in Austronesian (for the languages and branches see Supplementary Material S3.5–3.6; an example from Bunaq of the Timor-Alor-Pantar family is (39) above). All are from east of the Wallace Line, which in linguistic terms is the line east of which Papuan languages are found and most Austronesian languages have Papuan contact or substratal effects. Donohue and Schapper (2008) trace their origin in Austronesian to Papuan influence: calquing Papuan constructions and also more general Papuan-influenced pressure to use prenominal modifiers. In this view, the Oceanic construction is not an internal Proto-Oceanic development but a Papuan contact phenomenon. Since there is reason to believe that different Oceanic subgroups had different Papuan substrata (Donohue and Denham 2008), appositive possessive constructions in Oceanic may well result from separate post-Proto-Oceanic contact episodes rather than from a single Proto-Oceanic or Proto-Eastern-Malayo-Polynesian innovation. This would indicate that pre-Austronesian Oceania was a diverse hotbed in which appositive possessive constructions were a sizable minority tendency among the Papuan languages, and they survive as substratal properties in some Austronesian languages. No cases have been reported from inland New Guinea (or non-Austronesian New Guinea more generally), or from Australia.

The American group extends primarily from the U.S. southwest through Mexico and Central America to South America. In the U.S. and Mexico it chiefly involves the two widespread language families of the desert and semidesert interior, Uto-Aztecan and Yuman (yuma1250), and their neighbors. The speakers are mostly agricultural peoples or desert foragers with intensified techniques of plant gathering and use, or their neighbors.

There is a small cluster in the U.S. southeast, arguably stemming ultimately from the Lower Mississippi Valley (LMV), a region where agriculture spread early from Mexico and Central America, complex farming societies developed, and similar ecologies made overland and coastal migration from Mexico natural and probably common.22A specific case is Chitimacha (chit1248), a language isolate of the Mississippi delta, shown to be probably related to the Totozoquean macrofamily of Mesoamerica by Brown et al. (2014). D. Kaufman (2014) (q.v. for Lower Mississippi Valley areal properties more generally) finds that Totonac (toto1252), a Totozoquean member, scores high in structural resemblances to Lower Mississippi Valley languages. Four large language-family spreads, those of Caddoan, Siouan, Iroquoian, and Muskogean, originated at or near the periphery of the LMV, and all of them contribute examples of appositive possessives or non-possessibles to our survey.

The southwestern set continues into Mexico and then, in humid tropical environments, to Central America and South America, chiefly Amazonia. The speakers are mostly agricultural peoples. Thus the distribution of appositive possessives in the Americas largely coincides with the range of agriculture. Probably the best interpretation of this geography is that non-possessibles and appositive possessives have diffused through contact, and agricultural spreads testify to ecological continuity, which makes migrations and language contacts relatively easy. In the Pacific, horticulture is ancient on the islands and mainland New Guinea, but appositive possessives are found only on the islands and not on the mainland except where introduced by Austronesian settlement (one token in our survey). Diffusion of appositive systems here seems to follow cultural and economic lines rather than following cultivation per se, distinguishing coastally oriented from inland societies. The difference antedates the spread of Austronesian east of the Wallace line, over 4,000 years ago. Where anything is known about the sociolinguistics of the contacts, they have been intense, often substratal.

Language and language-family density are high all around the Pacific. We suppose that language contact and especially multilingualism increase tolerance of contact-induced reanalysis and enhance the likelihood that what would otherwise be a one-time-only novel construction or even a slip or outright mistake in a monolingual society would be accepted and even propagated.

In sum, appositive possessives appear to follow contact and diffusion lines and form large areas that reflect local language-to-language contacts within similar ecosystems. Appositive possessives are absent in large stretches of the near-coastal zone in the Pacific Northwest of North America, and in the Andean highlands and nearby in South America. We have no explanation for these gaps.

We found three large clusters where appositive possessives appear to be an areal or family tendency, found in a number of languages. In all three there is a mix of types of systems, including some large systems that have evidently expanded from smaller ones.

In the Pacific, small appositive systems in a number of Papuan languages are an occasional areal phenomenon in Oceania as described above, found east of the Wallace Line in a minority of languages representing several unrelated Papuan families and several Austronesian branches but absolutely lacking west of the line and in Australia and New Guinea. The appositive systems differ as to whether they are verbal or nominal, prefixal or suffixal, and in their forms, but nearly everywhere they are minimal or small systems; where there is more than one classifier there is generally one for edibles. Where they occur in Austronesian languages they are due to Papuan substratal influence (Donohue and Schapper 2008). One of those occasional cases must have been in Proto-Oceanic, whose proliferation over all of Oceania has given rise to hundreds of cases in the modern Oceanic languages. There have been three independent innovations of larger systems, all in the Oceanic branch of Austronesian: one in the Micronesian subbranch, one in the St. Matthias subbranch, and one in the New Caledonian subbranch (Supplementary Material S3.5.1). If the optional relative-like construction of Kiribati represents an earlier Micronesian stage (Supplementary Material S3.5.1), the ancestral construction must have originally been verbal.

In the Uto-Aztecan family (§4.2.1 and Supplementary Material S3.2.2), appositive systems are found in a number of languages in the central area of the family’s range. All but one are small, and several branches have a cognate appositive for animals, usually domesticates. Similar systems are found in the neighboring Yuman family and Washo isolate. A large system has arisen once, in Cahuilla (§4.2), where it is a transparent and evidently recent extension of the minimal system of its closest sisters. The system is verb-based and expanded by allowing the set of classifier verbs to become open-ended.

Despite widespread areality in Amazonia and nearby, there is considerable diversity from family to family in possessive constructions, with most families internally fairly consistent and discrete from others (Supplementary Material S3.4). Appositive systems, mostly small, are found in a number of Amazonian languages. Most Cariban languages have large, open systems of appositive nouns. Appositives meaning ‘animal’ and ‘food’ recur in nearly all Cariban languages, and are often cognate. Arawak languages typically have head-marked possessive morphology, and nouns are usually divided into possessive classes based on obligatoriness of possession and the presence of PSD suffixes when possessed and/or NPN suffixes when unpossessed (see §2.2 for the Yanesha’ system). In our sample, two Arawak languages have appositive possessives while two others have a system of classifiers that are not possessive but have more or less gender-like functions, appearing on various modifiers in agreement with the head noun. It appears that few Tupian languages have appositives, and those that do mostly have small systems. Seki (2000: 302–303) describes a large system in Kamaiurá, and, judging from her examples and her listing of the non-possessibles, the appositives can be used not only with non-possessibles but with other nouns as well. The classifiers include animals and food but also, curiously, ‘body, house’, ‘covering, wrap, clothing’, and ‘bow, weapon’, meanings that often surface among the obligatorily possessed nouns in other languages. These are the three large language families that are widespread in Amazonia. Several of the Amazonian isolates and non-Amazonian languages have small systems of appositives. Large systems have arisen at least twice in Amazonia: in an earlier stage of Cariban (or perhaps more than one early branch) and in Kamaiurá.

To summarize, occasional occurrence of small systems is part of an areal signature in three separate areas, and in each of the areas large systems are an occasional development of small systems, possible (but not frequent) where resources exist to expand the set of classifiers.

There is a consistent difference between the Americas and the Pacific involving the semantics of non-possessibles: they center on food in the Pacific and animals in the Americas. Table 1 shows the classifiers found in the smallest systems. Larger systems, in the Pacific, the Americas, and older Ainu, add subdivisions of food by preparation type; clothing; instruments and objects by function; fire and fire-related things; houses; various items by history of acquisition; and others. Of the core items in small systems, the food class is easily elaborated in the Oceanic languages but the animal one is not elaborated in the Americas.

**Table 1:** Numbers and semantics of classifiers in small systems. Glottolog codes are provided only for languages not mentioned in the text.

No. Classifiers	Semantics per class				Languages
	1st class	2nd class	3rd class	4th class	
*Americas:*					
1	pet				Shoshone, S. Paiute (sout2969), Washo, Luiseño (luis1254), Hopi (hopi1249), Acoma (acom1246), Creek (cree1270), recent Macushi
2	pet	food			Waimiri (waim1254), earlier Macushi
	pet	plants			Chemehuevi(chem1251)
	pet	inanimate			Cupeño, Yuman
	pet	paper			Copala Triqui (copa1237)
	pet	instrument, tool			Wichi Lhomtes
	animate	inanimate			
3	female	male	general		
	animal	animal			Kadiweu (kadi1248)
*Austronesian:*					
2	food	general			South Halmahera-West New Guinea (sout3231)
3	food	drink	general		Proto-Oceanic
4	food	drink	plant	general	Paamese
*Non-Austronesian Pacific (Papuan):*					
1	general				Various languages

### 5.2 Diachrony

Several processes of change in classifier systems can be inferred from our data. A small appositive classifier system can expand to a large one, as shown by comparing closely related Cupeño and Cahuilla (Uto-Aztecan) (§4.2 and Supplementary Material S3.2.2). Cupeño has a two-classifier verbal system distinguishing animal vs. general classifiers (a common pattern for central Uto-Aztecan languages and their neighbors); the classifier construction involves relativization. Cahuilla simply adds verbs to the system, creating a set of classifiers for foods and food plants by using verbs for conventional ways of acquiring possession and preparing foods. Some similar process of lexical expansion must have created the large systems of a few Oceanic languages (see Supplementary Material S3.5.1 for the Kiribati verbal appositive system as a possible early stage). However, the starting point – the classifier system reconstructed for Proto-Oceanic – is not verbal and consists of short morphemes that are dedicated classifiers.

Loss of classifiers can also be inferred. The several classifiers of earlier Ainu have shrunk to just one in the modern variety, and Macushi (§2.2, §4.2) probably lost all but one classifier during the 20th century. Obviously some classifiers have fallen out of use, probably as less fluent speech communities came to rely more and more on a single default classifier.

Classifier constructions and their semantic classes, though probably not the actual classifiers, evidently spread by contact. Evidence includes the strong resemblance between the small systems of Numic (Uto-Aztecan) languages and their neighbors Washo (isolate) and Arapaho (Algonquian) (§4.2, Supplementary Material S3.2.7), all of which distinguish an appositive ‘pet’ and often no more. An example of how contact operates is given by Berry and Berry 1999: speakers of Abun (abun1252) (which has a single appositive word), when speaking Indonesian (which does not have appositive possessives), construct possessive phrases with an appositive:

(56)

**Table:** 

*saya*	*punya*		*rumah*
1sg		POSS	house
‘my house’ (81)			

Standard Indonesian does not use *punya* in such phrases. The appositive is the verb ‘have, possess’ in Indonesian, so Abun-influenced Indonesian has a verbal appositive system.23Berry and Berry do not discuss the lexical source of the appositive *bi* of Abun, but gloss it POSS and describe it grammatically as a linker.


That is our sole piece of evidence for the rise of an appositive system where there was none before. It involves calquing, i.e. contact. We have no evidence on how appositive systems arise in the first place in the absence of contact influence. Donohue and Schapper (2008) note that a number of western Austronesian languages that have no appositive possessives do have two different possessive constructions (either with no reported semantic or pragmatic difference, or with only pragmatic difference), e.g. Tagalog (taga1270):

(57)

**Table:** 

Tagalog (Donohue and Schapper 2008: 319)

a.

**Table:** 

*ang*	*tiyan=ko*
NOM	stomach=1sg.GEN

b.

**Table:** 

*ang*	*aki(n)-ng*	*tiyan*	*∼*	*ang*	*tiya(n)-ng*	*akin*
NOM	1sg.DAT-POSS	stomach		NOM	stomach-POSS	1sg.DAT
all ‘my stomach’						

(a) is head-marked; (b) has a second-position linker (our POSS; in the source, LINK) which attaches to the first noun in the phrase, whether possessor or possessed. Donohue and Schapper argue that the availability of two models may facilitate the rise of an alienability distinction.

There is some evidence that the crucial step in the evolution of appositive systems is the development and grammaticalization of a class of non-possessibles. At least, there are languages, some on the outskirts of appositive-using areas, that have a set identified as non-possessible but without an identified adnominal construction for owned non-possessibles: Dakota (dako1258) and possibly Biloxi (Siouan; Supplementary Material S3.2.8.3), some Mayan languages (Supplementary Material S3.3.2), and our sole example from inland New Guinea, Koiari (footnote 3 above). Two sources report cases where items of modern technology were initially non-possessible but have become possessible with the ordinary head-marked possessive construction as they have become familiar and ownable in the culture. Tavares (2005: 147–149) describes Wayana (Cariban) non-possessibles as pertaining semantically to the wild or non-cultural world, while possessibles belong to human culture and their membership can be expanded, as items of national and European culture become familiar in Wayana society. Laughlin (1975: 24) relates how the Tzotzil (Mayan) word for ‘bus’ was firmly non-possessible when he began his fieldwork, but by 15 years later, when someone from a nearby village had bought a bus, the word was possessible.

All of these cases suggest that the essential starting point of non-possessibility is unfamiliarity or non-attestation of the noun with possessive inflection, i.e. that possessive inflection cannot easily be extended to a word that does not take it (as is of course the case for inflection in general). Grammaticalization of one or more inflectable words as appositives does not necessarily follow immediately on perception of a noun’s referent as ownable: the Iroquoian pattern of non-possessibles with recruitable ad hoc appositives but no dedicated appositives (Supplementary Material S3.2.8.2) appears to be durable in that family. The whole question needs more work, but it is interesting if the critical enabling factor is not something like the semantics of possession or the frequency of use with possessors but the covert argument structure of nouns.

Whatever the shorter-term evolutionary processes may have been, the modern distribution of appositive possessives must have resulted from a mix of contact and inheritance over a very long time span.

### 5.3 Measuring the strength of the Circum-Pacific effect

The distribution appears mostly consistent with Nichols and Bickel’s (2005a) hypothesis that appositive possession is characteristic of the Pacific Rim population.

However, that hypothesis is not about recent or current language contact nor about the current geographical distribution. Rather, it bears on probability differences during the entire history of the Circum-Pacific region: despite all fluctuations, linguistic lineages are hypothesized to have been more likely to develop, retain and/or re-develop possessive appositives because these lineages have been in contact at some time, or at several times, with families that show a similar bias. Proper testing of this hypothesis therefore requires an assessment of diachronic biases toward or against appositive possessives over the world’s language families. While such biases are best estimated through explicit evolutionary modeling, this is not feasible in our data since most families are very small (nearly half are isolates with only one member; Bickel 2015) and/or are known only through poorly resolved phylogenies.

In such situations, simple per-family proportions pick up bias signals much as explicit models do. Bickel (2020) demonstrates this for the per-family proportions estimated by the *Family Bias Method* (Zakharko and Bickel 2011), which we employ here. The basic idea of this method is to take the proportion of a feature in a family (e.g. 80% of the languages have appositive possessives) and evaluate whether this proportion differs from what can be expected by chance alone. If the proportion of appositive possessives is higher than expected, this indicates a bias toward retaining and/or (re-)developing appositive possessives over time; if it is lower, this indicates a bias toward losing and not developing appositive possessives; if it is expected by chance, this indicates random evolution and the absence of a bias. Estimates of this kind can easily be calculated for large families. For isolates and small families, the Family Bias Method estimates biases with an extrapolation sampling algorithm. The algorithm assumes that the isolates and members of small families are the survivors of unknown larger families with a similar proportion of biases (for or against appositive possessives) as opposed to no biases as in the known large families. Given this assumption, the method samples survivors in proportion to the large-family bias proportion and declares them to belong to biased unknown families as well. For the resulting sample of survivors, we furthermore assume that some of them faithfully reflect the bias in their unknown larger family, and so we can take their choice (e.g. having appositive possessives) to reflect that bias. But some will happen to be deviates, e.g. the exceptional language that lost appositive possessives although its family as a whole was biased to retain them. To allow for this possibility, the method declares a subsample to be deviates, in inverse proportion to the strength of the bias in large families, i.e. a smaller subsample if the large-family biases are stronger (e.g. on average 90% presence) and a larger one if they are weaker (e.g. 70% presence).

The extrapolation algorithm involves sampling in two cases (whether a survivor stems from a biased vs. non-biased family; whether it reflects the bias or deviates from it). While the size of the samples is set by the large-family proportions, the concrete choice of languages within these samples is random. To accommodate the uncertainty from this, we repeat the sampling procedure many times.

**Figure 2:**
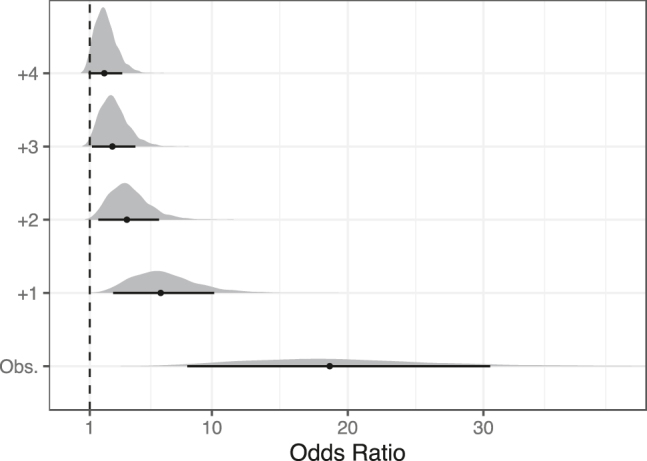
Estimated ratio between the odds of families being biased toward appositive possessive inside vs. outside the Circum-Pacific region. The dashed line indicates an odds ratio of 1, i.e. equal odds for appositive possessive biases inside and outside the region. The estimates of the odds ratios are plotted as densities across the large family data and all sampled extrapolations, so the heights of the gray areas indicate how many samples have a given odds ratio (*x*-axis). The black dots represent the median across the samples and the black horizontal lines represent the 90% highest density of estimates around the median (leaving 5% on each side). The bottom density (“obs.”) reports the estimates from the observed data, as reported in Supplementary Material S1. The other densities are counterfactual estimates and report how the odds ratio would look if +1, +2, +3, or +4 more families or isolates turned up with appositive possessives outside the Circum-Pacific region (cf. Table S2 in Supplementary Material S2 for a numerical summary).

Figure 2 summarizes the results of a Family Bias analysis in terms of *odds ratios*, which report how many times higher the odds are for families to be biased toward vs. against appositive possessives in the Circum-Pacific as opposed to families outside the region.24The odds is a commonly used statistic that allows compact representation of relative probabilities. An odds of 1 means that something is as probable to occur as not, i.e. it stands a 1:1 chance of occurring. The odds ratio is the ratio between two odds, e.g. if A has odds of 1 (i.e. stands a 1:1 chance) and B odds of 2 (stands a 2:1 chance), then the odds ratio between A and B is ½: A has half the odds of B and B has twice the odds of A (see e.g. Agresti 2002: Ch. 2 for a comprehensive textbook discussion). The details of the statistical analysis are summarized in Supplementary Material S2. A replicable script and the data are available at https://osf.io/p5xky. The figure also includes the results of a *Reliability Landscape* analysis comparing the estimated odds ratio with counterfactual scenarios (Janssen et al. 2006): how many more families (one more? two more? etc.) would have to turn up with a bias toward appositive possessives outside the Circum-Pacific in order for the effect to disappear (i.e. to reach each equal odds, a ratio of 1:1 = 1)?

Our results suggest that the odds for appositive possessive biases are about 18 times higher inside the Circum-Pacific than outside it, with all estimates far away from equal odds (Figure 2). The effect is also relatively reliable against finding up to three families with estimated biases outside the Circum-Pacific. Note that this reliability estimate is highly conservative since it is of course just as likely that we missed a family with a bias *inside* the Circum-Pacific. A case in point is the South Halmahera and Western New Guinea branch of Oceanic, for which there is actually good evidence that it is biased toward appositive possessives (cf. §5.1) although we did not fully survey the branch and therefore have left it out from our quantitative analysis. Another possibility is Koiari, also left out of the quantitative analysis.

## 6 Conclusions

### 6.1 Is Ainu a typical Circum-Pacific language?

Where possessive marking is concerned, the answer to this question is Yes. Ainu has an appositive possessive construction for alienable nouns, which in Ainu are optionally possessed nouns. Inalienables are obligatorily possessed, and they take head-marked person indexation. Ainu uses a verbal appositive, a type that is not frequent overall but is well represented in the Pacific, and the minimal one-classifier system of recent Ainu finds a close parallel in several Papuan languages that also use a verb ‘have’ as their only possessive appositive. The larger system reconstructible for earlier Ainu is reminiscent of the Cahuilla system, which is also verbal; in both, the classifiers have to do with the history and cultural status of possession. The rapid shrinking from the earlier larger Ainu system to the recent minimal one, and the durability of the minimal one through language death, recall the expansions and reductions of the inventory of appositives that we can infer from the variation in large families like Cariban and Uto-Aztecan. The system remains an appositive possessive system and a core set of classifiers is retained regardless of the number of appositives. Ainu uses unspecified possessor and antipassive morphology as NPN morphology, allowing an obligatorily possessed noun to be used without a specified possessor or an overt possessor (15–18). PSD suffixes register a possessor but do not index its properties, and they mark a noun as requiring a possessor (which is then indexed with a possessive prefix). All in all the Ainu system, with its bound inalienables and non-possessible alienables, represents a common type among Circum-Pacific languages.

### 6.2 Why only Circum-Pacific?

In the Americas, appositive possessives are not strictly a Pacific Rim phenomenon but extend into the interior in North America, probably via contact with the southwest and Great Basin and/or the Lower Mississippi Valley and Southeast, and in South America across the northern half of the continent. Outside of these two American clusters, however, they are a strictly Pacific Rim phenomenon. In the southern Pacific they are found throughout eastern Oceania but not in New Guinea (except for a few Austronesian immigrant languages, one in our survey) or Australia. In northern Eurasia Ainu is one of four coastal families (along with Nivkh, Chukotko-Kamchatkan, and Eskimo-Aleut) surviving from what must have been a diverse population prior to the post-Neolithic spreads of language families of the Inner Asian type: Tungusic, Turkic, Mongolic, Japanese, Korean. We do not know what preceded them in the near-coastal area, but the synchronic situation is that appositive possessives are absolutely lacking on the Eurasian mainland.

The typological prerequisite of head-marking NP morphology is widespread throughout the Americas and northern New Guinea, and in northern Eurasia it is found in Tungusic both near the coast and inland, and in Turkic and isolate Yukagir farther inland, so there is no obvious typological obstacle to further spread. Appositives are evidently ancient in both the Americas and Oceania, so there has been enough time that further diffusion could have occurred. The fact that appositives are largely coextensive with agriculture in the Americas and are widespread in early horticultural Oceania may be telling: food production brings larger, denser and more complex social networks, and these increase the chances that an innovation will arise, catch on, and/or be further transmitted (for these demographic effects on innovation and transmission see Fagyal et al. 2010).

### 6.3 Summary

The main typological contributions of this paper are:

– An appositive possessive is a construction where a lexeme or morpheme in apposition to the possessed noun bears the possessive morphology, usually person or person-number indexation of the possessor.
– The set of appositive words is often classificatory, classifying possessed nouns by their semantic properties. The classification is usually semantic rather than lexical.
– Nouns can have argument structure and valence. Alienability and possessibility distinctions are a matter of argument structure; the form of their morphological marking as well as the grammatical categories they index is a matter of valence; and derivational or inflectional processes that change alienability or possessibility classes are matters of valence change. NPN and PSD (as defined in §2.2) commonly implement valence changes.
– Non-possessible nouns preclude morphological indexation of a possessor. Their referents can be owned, and appositives are the usual way of treating an owned non-possessible.
– Appositives are usually inalienably possessed nouns, but they can also be verbs, usually in the form of grammaticalized relative constructions. They too can be classificatory, utilizing different verbs.
– Possessibility classes and appositives are found only in languages with head-marked possession. This refers to strict lexical categorization, which we distinguish from discourse frequencies and tendencies. In languages like English, with dependent-marked possession, there is a strong tendency for kin and body-part terms to occur with possessors, but this is not a categorical requirement.
– Ainu possession is entirely typical and well covered by this framework.

The main contributions to historical linguistics are:

– Appositive possessives are almost entirely limited to the Pacific Rim macroarea, with extensions into North and South America at the known leakage points of the Lower Mississippi Valley and Amazonia. This range can be described as Circum-Pacific (but strictly Pacific Rim outside of the Americas).
– A language’s chances of developing an appositive possessive are about 18 times as great in the Circum-Pacific as elsewhere. This is a calculation of its combined birth and death rates.
– Typological prerequisites to developing or copying appositive possessives are head-marking morphology and possessibility oppositions in nouns.
– Appositive possessive constructions, their semantic classificatory principles, and occasionally the forms of their markers apparently diffuse along trade and contact networks and can eventually come to characterize areas.
– Ainu is easily interpreted as a remnant of what may have been a larger set of north Asian Circum-Pacific languages with appositive possession. Any others have been absorbed in the spreads of Tungusic, Japanese (nucl1643), Korean (kore1280), Chinese (mand1415), and Austronesian.

Questions that remain open are:

– How does appositive possession arise in the first place? We have examples of diffusion and copying but no known examples of spontaneous and entirely independent innovation.
– Why is appositive possession limited to the Circum-Pacific? We have suggested that greater density of human population and language diversity in the region creates more opportunities for reanalysis and diffusion, including diffusion that lets what would otherwise be a one-time or accidental formation be laterally transmitted and retransmitted often enough to outpace the expected die-off rate. This remains only a hypothesis, however.
– Why is non-possessibility categorical only in languages with head-marked possession? After all, in clauses, languages with no head-marked argument indexation, such as Dyirbal or Japanese, have firm argument-structure classes of verbs.
– Is it true that (as suggested in §5.2) non-possessibility can exist and be fairly stable in the absence of grammaticalized appositive constructions?

A presently unanswerable question is whether any evidence (such as substratal effects) can be found that languages now absorbed in the large spreads of northern Asia had appositive constructions. This would let us firmly classify the Ainu possessive system as a sole survivor rather than a random outlier. We hope that future typological research will find criteria and propose answers.

We close with a call for data. For very few languages are all the phenomena defined in §2.2 covered in grammars. We hope this paper will spur publication of comprehensive descriptions of possession and possessibility.

## Supplementary Material

Supplementary Material Details
