# Supplementary material for: Appositive possession in Ainu and around the Pacific
Source: Linguist Typol. 2021 Jun 9;26(1):43–88. doi: 10.1515/lingty-2021-2079 (PMC11585906; doi:10.1515/lingty-2021-2079)
Supplement: Supplementary file 1 — Supplementary Material Details [file j_lingty-2021-2079_suppl.pdf]

Anna Bugaeva*, Johanna Nichols*, and Balthasar Bickel*

Appositive possession in Ainu and around the Pacific

Supplement 1. Survey language metadata

https://doi.org/10.1515/lingty-2021-2079
Received August 1, 2019; accepted January 12, 2021; published online June 1, 2021

Table S1. Survey languages and main survey feature

Language	Stock	Branch	Glottocode	Appositive Possession presence
Ainu	Ainu	(isolate)	ainu1240	present
Pipil	Uto-Aztecan	SUA: Aztecan	pipi1250	absent
Cora	Uto-Aztecan	SUA: Corachol	elna1235	absent
Tümpisa Shoshone	Uto-Aztecan	NUA: Numic	pana1305	absent
Eastern Shoshone	Uto-Aztecan	NUA: Numic	shos1248	absent
Northern Paiute	Uto-Aztecan	NUA: Numic	nort2954	absent
Chemehuevi	Uto-Aztecan	NUA: Numic	utes1238	present
Southern Paiute	Uto-Aztecan	NUA: Numic	utes1238	present
Tübatulabal	Uto-Aztecan	NUA isolate	tuba1278	present
Hopi	Uto-Aztecan	NUA isolate	hopi1249	present
Choguitá Rarámuri	Uto-Aztecan	SUA: Tarahumara-Warihio	lowl1265	present
SE Tepehuan	Uto-Aztecan	SUA: Tepiman	sout2976	present
Tohono O'odham	Uto-Aztecan	SUA: Tepiman	toho1245	present
N Tepehuan	Uto-Aztecan	SUA: Tepiman	nort2959	present
Luiseño	Uto-Aztecan	NUA: Cupan	luis1253	present
Cupeño	Uto-Aztecan	NUA: Cupan	cupe1243	present
Cahuilla	Uto-Aztecan	NUA: Cupan	cahu1264	present
Seri	Seri	(isolate)	seri1257	present
Washo	Washo	(isolate)	wash1253	present
Hualapai	Yuman	Pai	hava1248	present
Jamul Tiipay	Yuman	Delta-California	kumi1248	present
Maricopa	Yuman	River	mari1440	present
Cocopa	Yuman	Delta-California	coco1261	present
Kiliwa	Yuman	Kiliwa	kili1268	present
Acoma	Keresan	Western Keresan	west2632	present
Laguna	Keresan	Western Keresan	east1472	present

Corresponding authors: bugaeva@rs.tus.ac.jp; johanna@berkeley.edu; balthasar.bickel@uzh.ch
For affiliations see main article.

Language	Stock	Branch	Glottocode	Appositive Possession presence
Wiyot	Algic	Wiyot	wiyo1248	absent
Yurok	Algic	Yurok	yuro1248	absent
Arapaho	Algic	Algonquian: Arapahoic	arap1274	present
Menomini	Algic	Algonquian: E.Great Lakes	meno1252	absent
Creek	Muskogean	Creek	cree1270	present
Cherokee	Iroquoian	Southern	cher1273	present
Tutelo	Siouan	Southeastern	tute1247	present
Lakhota	Siouan	Mississippi Valley	lako1247	present
Crow	Siouan	Missouri River	crow1244	absent
Wichita	Caddoan	Northern Caddoan	wich1260	absent
Haida	Haida	Northern	haid1248	absent
Coast Tsimshian	Tsimshianic	Southern-Coastal	nucl1649	absent
Kwak'wala	Wakashan	Northern Wakashan	kwak1269	absent
Nuuchahnulth	Wakashan	Southern Wakashan	nuuc1236	absent
Thompson	Salishan	Interior Salish	thom1243	absent
Musqueam	Salish	Central Salish	musq1240	absent
Slave	Athabaskan	Northern Athabaskan	nort2942	absent
Navajo	Athabaskan	Apachean	nava1243	absent
Hupa	Athabaskan	Pacific Coast	hupa1239	absent
Chimariko	Chimariko	(isolate)	chim1301	absent
Tz'utujil	Mayan	Quiche-Mam	tzut1248	absent
K'ichee'	Mayan	Quiche-Mam	kich1262	present
Mam	Mayan	Quiche-Mam	mamm1241	present
Yucatec	Mayan	Yucatecan	yuca1254	present
Chichimeco Jonaz	Otomanguean	Otopamean	chic1272	present
Copala Triqui	Otomanguean	Mixtecan	copa1247	present
Wayana	Cariban	Guianan	waya1269	present
Panare	Cariban	Venezuelan	enap1235	present
Tiriyó	Cariban	Guianan	trio1238	present
Apalai	Cariban	Apalai	apal1257	present
Waimiri	Cariban	Yawaperi	waim1253	present
Macushi	Cariban	Pemong-Panare	macu1259	present
Yanesha'	Arawak	W. Maipuran	yahe1238	absent
Achagua	Arawak	N Maipuran	acha1250	present
Piapoco	Arawak	N Maipuran	piap1246	present
Nanti	Arawak	S Maipuran	nant1250	absent
Kukama-Kukamiria	Tupian	Tupi-Guaraní III	coca1259	absent
Kamaiurá	Tupian	Tupi-Guaraní isolate	kama1373	present

Language	Stock	Branch	Glottocode	Appositive Possession presence
Guaraní	Tupian	Tupi-Guaraní: Southern	para1311	present
Tapiete	Tupian	Tupi-Guaraní I	tapi1253	absent
Emerillon	Tupian	Tupi-Guaraní VIII	emer1243	present
Wichi Lhomtes	Matacoan	Mataguayo II	wich1261	present
Kadiweu	Guaycuruan	Kadiweu	qomm1235	present
Itonama	Itonama	(isolate)	iton1250	absent
Urarina	Urarina	(isolate)	urar1246	present
Kwaza	Kwaza	(isolate)	kwaz1243	present
Yurakare	Yurakare	(isolate)	yura1255	present
Bunaq	Timor-Alor-Panta	East Timor-Bunaq	buna1278	present
Blagar	Timor-Alor-Panta	Alor-Pantar	blag1240	present
Klon	Timor-Alor-Panta	Alor-Pantar	kelo1247	present
Adang	Timor-Alor-Panta	Alor-Pantar	adan1251	present
Fijian	Austronesian	Oceanic: Central Pacifi	fiji1243	present
Paamese	Austronesian	Oceanic: N-C Vanuatu	paam1238	present
Iaai	Austronesian	Oceanic: Loyalties	iaai1238	present
Mussau	Austronesian	Oceanic: St. Matthias	muss1246	present
Gilbertese	Austronesian	Oceanic: Micronesian	gilb1244	absent
Ambai	Austronesian	SHWNG	amba1265	present
Selaru	Austronesian	SHWNG	sela1259	present
Sawai	Austronesian	SHWNG	sawa1247	present
Banggai	Austronesian	Celebic	bang1368	present
Tugun	Austronesian	CEMP: Timoric	tugu1245	present
Kaitetu	Austronesian	CEMP:Central Maluku	seit1239	present
Motuna	S.Bougainville	Buin	siwa1245	present
Abun	Abun	(isolate)	abun1252	present
Mpur	Mpur	(isolate)	mpur1239	present
Kol	Kol	(isolate)	kolp1236	present
Sulka	Sulka	(isolate)	sulk1246	present
Bilua	Bilua	(isolate)	buky1245	present
Kuot	Kuot	(isolate)	kuot1243	absent
Mali	Baining		mali1284	absent
Kosraean	Austronesian	Oceanic: Micronesian	kosr1238	present
Marshallese	Austronesian	Oceanic: Micronesian	mars1254	present
Puluwatese	Austronesian	Oceanic: Micronesian	pulu1242	present
Ulithian	Austronesian	Oceanic: Micronesian	ulit1238	present
Mokilese	Austronesian	Oceanic: Micronesian	moki1238	present
Pohnpean	Austronesian	Oceanic: Micronesian	pohn1238	present

Anna Bugaeva, Johanna Nichols, and Balthasar Bickel

Appositive possession in Ainu and around the Pacific:

Supplement 2. Statistical analysis *

S2.1. Data

AUTOTYP (Bickel et al. 2017) only contains few entries on non-possessibles, but we collected more data for WALS (Nichols & Bickel 2005). We read in the WALS data via the R package lingtypology (Moroz 2020) so we have convenient access to Glottocodes for data matching. We re-code the entries as binary and remove Hixkaryana because closer inspection shows the data is in fact unclear.

```
wals.df <- wals.feature('58b') %>%
  rename_with(str_to_title) %>%
  filter(!Language %in% "Hixkaryana") %>%
  mutate(AppPoss.Presence = ifelse(`58b` %in% 'None reported', 'absent',
                                                   'present'))
```

We merge this data set with the appendix of the main paper, giving precedence to the newer data. We furthermore add genealogical and geographical information from the AUTOTYP register (v. 0.1.2) and define the Circum-Pacific area as in previous work (Bickel & Nichols 2005). Continent and Area definitions are explained and visualized in the documentation of the AUTOTYP repository (Bickel et al. 2017).

```
appendix.df <- read.xlsx('Supplement1.xlsx', startRow = 2)

poss.df <- rbind(appendix.df[,
                             c('Glottocode','AppPoss.Presence')],
                 wals.df[!wals.df$Glottocode %in% appendix.df$Glottocode,
                         c('Glottocode','AppPoss.Presence')])

poss.g.df <- inner_join(poss.df,
  read.csv('https://raw.githubusercontent.com/autotyp/autotyp-data/v0.1.2/data/Register.csv')) %>%
  # removing multiplication by doculects that share the same Glottocode and AppPoss value:
  distinct(Glottocode, AppPoss.Presence, .keep_all = T) %>%
  mutate(AncientArea = ifelse(Continent %in% c('Australia',
                                               'NG and Oceania',
                                               'W N America',
                                               'S America',
                                               'C America',
                                               'E N America') |
                                Area %in% 'N Coast Asia',
                              "Inside Circum-Pacific", "Outside Circum-Pacific"),
         AN = ifelse(Stock %in% 'Austronesian', 'Austronesian', 'Other')
        )
```

S2.2. Family bias analysis

Only few of the families with appositive possessives have sufficiently resolved phylogenies. In such situations, simple per-family proportions pick up similar signals as explicit evolutionary models in an

*This document was rmarkdown::render'ed from an R script available at https://osf.io/p5xky

Corresponding author: balthasar.bickel@uzh.ch For author affiliations see main article.

MCMC framework (Bickel 2020). A suitable proportion method is the *Family Bias Method* (Zakharko & Bickel 2011; Bickel 2011), which also performs extrapolations for estimating biases in small families and isolates (Bickel 2013) by combining the observed data with empirically trained priors on biases (in any direction) vs. non-biases (chance developments).

```
poss.fam <- familybias(poss.g.df,
                       family.names = c('Stock',
                                        'MajorBranch',
                                        'SubBranch',
                                        'SubSubBranch',
                                        'Language'),
                       r.name = 'AppPoss.Presence', p.names = 'AncientArea',
                       lapplyfunc = mclapply, B = 4000) # number of extrapolations
```

We summarize the results by limiting our attention to families estimated as biased towards or against appositive possessives because the others are uninformative (Bickel 2013). Figure S1 plots the estimates with the uncertainty from the extrapolation runs.

```
poss.fam.e <- do.call(rbind, mclapply(poss.fam$extrapolations, function(e) {
   df <- as.data.frame(xtabs( ~ majority.response + AncientArea,
                       subset = distribution  %in% 'biased', data = e,
                       drop.unused.levels = T))
  df$majority.response <- factor(df$majority.response, levels = c('present', 'absent'))
  return(df)
}))
```

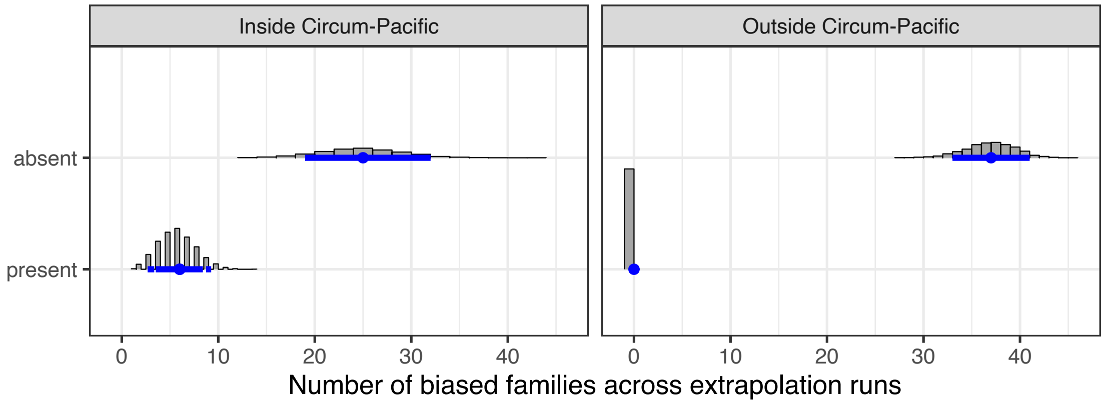

Figure S2.1: Estimated number of families with a bias towards appositive possessives ("present") vs. against appositive possessives ("absent"). Blue dots represent medians and blue horizontal lines the 90% highest density interval.

We capture the main effect of interest in terms of *Odds Ratios (OR)*, which report how many times higher the odds for vs. against appositive possessive biases are inside as opposed to outside the Circum-Pacific region. Our estimates for appositive possessive biases outside the Circum-Pacific are all zero, and so we add a .5 correction for continuity (Agresti 2002).

We also assess the *Reliability Landscape* of the effect (Janssen et al. 2006), comparing the estimated OR with counter-factual scenarios: how many more families might one discover with a bias towards appositive possessives outside the Circum-Pacific so that the effect would disappear? We include these estimates in Figure 2 of the main text, with 90% highest density intervals (HDI). Table S2 gives a numerical overview.

Our Reliability Landscape is conservative since we only explore what goes against our hypothesis. Of course, it is just as likely that we underestimate the appositive possessive biases in the Circum-Pacific, which would increase the effect. A case in point is the South Halmahera and Western New Guinea branch of Oceanic, for which there is actually good evidence that it is biased towards appositive

possessives (see the discussion in the main paper), although we didn't survey the branch at the level of detail required for the present analysis.

```
poss.fam.ORs <- do.call(rbind, mclapply(poss.fam$extrapolations, function(e) {
  e <- subset(e, distribution  %in% 'biased')
  e$majority.response <- factor(e$majority.response,
                                levels = c("absent", "present"))
  tab <- xtabs( ~ majority.response + AncientArea,
                data = e, drop.unused.levels = F) + .5
  # odds of presence:absence inside divided by the same odds outside:
  data.frame(OR = (tab[2,1]/tab[1,1])/(tab[2,2]/tab[1,2]),
             # adding 1...4 to "present outside CP" (cell 2,2):
             ORplus1 = (tab[2,1]/tab[1,1])/((tab[2,2]+1)/tab[1,2]),
             ORplus2 = (tab[2,1]/tab[1,1])/((tab[2,2]+2)/tab[1,2]),
             ORplus3 = (tab[2,1]/tab[1,1])/((tab[2,2]+3)/tab[1,2]),
             ORplus4 = (tab[2,1]/tab[1,1])/((tab[2,2]+4)/tab[1,2])
  ) })) %>%
  pivot_longer(cols = everything(), names_to ="Scenario", values_to = "OR") %>%
  mutate(Scenario = factor(Scenario,
                           levels = unique(Scenario),
                           labels = c('Obs.', '+1', '+2', '+3', '+4')))
```

Table S2.2: OR estimates in the observed data and counterfactual situations (like in Figure 2 of the main text), with 90% highest density intervals

Scenario	Median	90%CI
Obs.	18.67	[8.19, 30.49]
+1	6.22	[2.73, 10.16]
+2	3.73	[1.64, 6.1]
+3	2.67	[1.17, 4.36]
+4	2.07	[0.91, 3.39]

References

Agresti, Alan. 2002. *Categorical Data Analysis*. New York: Wiley-Interscience.

Bickel, Balthasar. 2011. Statistical Modeling of Language Universals. *Linguistic Typology* 15. 401–414.

Bickel, Balthasar. 2013. Distributional Biases in Language Families. In Balthasar Bickel, Lenore A. Grenoble, David A. Peterson & Alan Timberlake (eds.), *Language Typology and Historical Contingency*, 415–444. Amsterdam: Benjamins.

Bickel, Balthasar. 2020. Large and Ancient Linguistic Areas. In Mily Crevels & Pieter Muysken (eds.), *Language Dispersal, Diversification, and Contact: A Global Perspective*. Oxford: Oxford University Press.

Bickel, Balthasar & Johanna Nichols. 2005. Inclusive/Exclusive as Person Vs. Number Categories Worldwide. In Elena Filimonova (ed.), *Clusivity*, 47–70. Amsterdam: Benjamins.

Bickel, Balthasar, Johanna Nichols, Taras Zakharko, Alena Witzlack-Makarevich, Kristine Hildebrandt, Michael Rießler, Lennard Bierkandt, Fernando Zúñiga & John B Lowe. 2017. *The AUTOTYP Typological Databases, Version 0.1.2*. GitHub [https://github.com/autotyp/autotyp-data].

Janssen, Dirk, Balthasar Bickel & Fernando Zúñiga. 2006. Randomization Tests in Language Typology. *Linguistic Typology* 10. 419–440.

Moroz, George. 2020. `lingtypology`: Easy Mapping for Linguistic Typology. R package, https://CRAN.R-project.org/package=lingtypology, v. 1.14.

Nichols, Johanna & Balthasar Bickel. 2005. Possessive Classification and Obligatory Possessive Inflection. In Martin Haspelmath, Matthew S. Dryer, David Gil & Bernard Comrie (eds.), *The World Atlas of Language Structures*, 242–245. Oxford: Oxford University Press.

Zakharko, Taras & Balthasar Bickel. 2011. `familybias`: Family Bias Estimation. R package, https://github.com/IVS-UZH.

Anna Bugaeva*, Johanna Nichols*, and Balthasar Bickel*

Appositive possession in Ainu and around the Pacific:
Supplement 3. Survey language data

https://doi.org/10.1515/lingty-2021-2079
Received August 1, 2019; accepted January 12, 2021; published online June 1, 2021

Abstract: This supplement has two goals: (1) to give information on possessibility classes and possessive marking in all languages we surveyed that have appositive constructions or other non-possessibility; (2) to make available a body of data with consistent analyses, terminology, and glosses. Languages are ordered by continent and then family, clockwise from Ainu. The sections in order are:

S3.1. Ainu
S3.2. North America
S3.3. Mexico-Central America
S3.4. South America
S3.5. Pacific: Austronesian
S3.6. Pacific: Non-Austronesian
S3.7. References

S3.1. Ainu (ainu1240)

Additional examples of old classificatory verbs are the following.

(S1) *a-**mi*** *kosonte* *a-e-oriki-kut-kor* *kane*
4.A-wear Japanese.kimono 4.A-APPL-upwards-belt-have doing.so
'I put my Japanese kimono on.' (Satō 2003)

(S2) *a-**mi*** *hayokpe* *opitta* *a-yay-ko-anu* *wa*
4.A-wear armor all 4.A-REFL-to.APPL-put and
'I took off all my armor' (Nakagawa 1995)

The nouns attested with *mi* 'wear' are limited to *kosonte* 'Japanese short-sleeved kimono' and *hayokpe* 'armor'. The referent of the latter is also of Japanese origin, which additionally shows that the essential starting point of non-possessibility is unfamiliarity.

(S3) *a-**resu*** *kamuy* *a-**resu*** *pito* *e-i-nu*
4.A-raise god/spirit 4.A-raise god/spirit 2SG.S-APASS-hear
kat-u *ene* *an* *hi*
shape-POSS like.this exist.SG NMLZ
'My spirit, my spirit, listen carefully to what I have to say.', lit. '…your listening shape is like this.' (Kubodera 1977: 450)

Corresponding authors: bugaeva@rs.tus.ac.jp; johanna@berkeley.edu; balthasar.bickel@uzh.ch
For affiliations see main article.

Here too we are dealing with two old loanwords from Japanese, i.e. *kamuy* 'god/spirit' (< OJ *kamï* 'god, spirit') and *pito* 'god/spirit' (< OJ *pito* 'man, human'), which is consistent with the above scenario. Other nouns attested with *resu* 'raise' are *pewre-p* 'bear-cub', which was ritually raised in a cage in Ainu villages and killed to be sent off to the land of gods, and *ca-pe* (onomatopoeia-thing) 'cat', whose referent is borrowed from the Japanese.

A number of kin terms in Ainu are variable in that they can appear as both obligatorily possessed and non-possessible with *kor* 'have'.

(S4a) ***a****-mat-****ihi***
4.A-wife-PSD
'my wife' (Chiri and Kindaichi 1936(1974): 28)

(S4b) *a-****kor*** *mat*
4.A-have wife
'my wife' (Chiri and Kindaichi 1936(1974): 28)

The semantics of affection seems to be a factor that can determine the choice, but the structural fact is that these words are variable.

Moreover, some kinship terms are also attested in puzzling obligatory possessed and non-possessible combinations, which are regarded as symptoms of language attrition in Chiri and Kindaichi (1936(1974): 29) and Chiri (1942(1973): 533). Yet, such examples are not rare in Classical Ainu literature *yukar* 'heroic epics' and *kamuy yukar* 'divine songs', and on top of this they are found with other classificatory verbs too as in (S6) and (S7), cf. (25) in the main text.

(S5) *a-****kor*** *mac-****ihi***
1SG.A-have wife-PSD
'my wife' (Chiri and Kindaichi 1936(1974): 29)

(S6) *a-****an-te*** *mac-****i*** *itak-an ciki*
4.A-exist-CAUS wife-PSD speak-4.S if
pirka-no e-nu kus ne na
be.good-ADV 2SG.A-hear because COP SGST
'My wife, if I speak, you should listen well.', lit. 'lit. 'husband (who) I made stay (with myself)' (Bugaeva 2004: 191)

(S7) *an-****ante*** *hoku-****hu*** *e-ikka kusu*
4.A-exist.SG-CAUS husband-PSD APPL-steal because
'She stole my husband.', lit. 'husband (who) I made stay (with myself)'
(Kubodera 1977: 244)

Kinship terms are likely to be the first pioneers moving out of the obligatorily possessed class so obligatory possessed while non-possessible combinations can be regarded as an intermediate stage in this process of change in classification in Ainu, which is valuable for understanding the diachrony of how word classes evolve.

S3.2. North America

For the Pacific Northwest (Alaska to northern California), all languages surveyed lack appositive possession.

S3.2.1. Wakashan

Nuuchahnulth (nuuc1236, Southern Wakashan; Vancouver Island, Canada): There is no appositive possession, but apparently all nouns can optionally be treated as non-possessible. Possession can be marked either on the verb, as external possession indexing the possessor (and no possessive marking on the noun), or on the noun with subordinating or relative morphology (Nakayama 2001:128, Ravinski 2005). The PSD suffix makes an alienability distinction (Ravinski 2005) and 'wife' is alienable.

(S8) *7aap-h.ii-7a:k-s* *łuucma*
kind-DUR-PSD:AL-1sg wife
'my wife is kind' (Nakayama 128)
(lit. 'my-kind-one, wife') 7 = glottal stop; h. = h with underdot

(S9) *7aap-h.ii-7iš* *łuucma-7a:k-qs*
kind-DUR-IND.3 wife-PSD:AL-SUB.1sg
(lit. 'she-is-kind, my-wife')
id. (ibid.)

S3.2.2. Uto-Aztecan

The Uto-Aztecan family extends well into both North America and Mexico, but for convenience all of it is covered in this section. The family is divided into two halves, for which we use the abbreviations NUA = Northern Uto-Aztecan and SUA = Southern Uto-Aztecan.

Most Uto-Aztecan languages have possessive affixes or clitics indexing the person-number of the possessor, e.g. Cupeño (cupe1243, NUA: Cupan):

(S10) *nu-yu*
1sg-hair
'my hair' (Hill 2014:167)

Most make an alienable/inalienable distinction. Commonly, inalienables are obligatorily possessed and alienables optionally possessed. Inalienables usually include kin terms, body parts, and a few other close possessions.

In addition, most of the languages have a set of NPNs (Uto-Aztecanist term: *absolutive* suffixes) found on nouns that do not have possessive morphology or other suffixal morphology or compounded elements. Some of the languages also have PSD suffixes that cooccur with possessive indexation. E.g. Pipil (pipi1250, SUA: Aztecan):

(S11) *siwa:-t* *nu-siwa:-w* *-siwa:-*
woman-NPN 1sg-woman-PSD
'woman' 'my wife' 'wife' (stem) (Campbell 1985:38, 43)

Cupeño:
(S12) *waka-l* *-waq-ʔa* *-waq-*
spear-NPN spear-PSD
'spear, a spear' 'spear' (lexeme) 'spear' (stem)
(Hill 2014:172)

See also Sapir (1930:120–122) for Southern Paiute (utes1238) examples, and Langacker (1977:88).

Several of the languages have a set of non-possessible nouns, always including animal names and sometimes plants and natural phenomena. When the referent of an alienable is owned an appositive construction with a classifier noun is used. E.g. Cupeño (Hill 2014:129, 176–177):

(S13) *awal* *ne-ʔash*
dog 1sg-CL:pet
'my dog'

(S14) *wi-lʸ* *ne-mixan*
lard-NPN 1sg-CL:possession
'my lard' (cooking lard) [constructed from Hill's table (176)]

Languages at the far peripheries of the family's range have minimal or no appositive possession. Pipil (Aztecan subbranch; Campbell 1985), spoken in Nicaragua at the far southern limit of the Aztecan branch, has none (nor do other Aztec varieties to our knowledge). In western central Mexico, Cora (elna1235, SUA: Corachol subbranch, just north of Aztecan; Casad 1984) appears not to have appositives or classifiers, but in predicative possession the word 'animal' (sg. *kyi*, pl. *yá'amwa*) is inflected for possession and replaces the verb 'be' used for other possession:

(S15) *ńéeci* *pú* *ńa-kyi*
mine 3sg 1sg-animal
'It's my animal' (236)

(S16) *nye-síiku'u* *pú=pýryky*
1sg-shirt 3sg=be
[it's my shirt] (234)

This may be either a fossil or a possible nascent appositive construction.

The northernmost languages of the Numic branch (the northernmost Uto-Aztecan branch, spoken in and near the Great Basin) also lack appositive constructions in its northernmost languages. All the Numic languages have a noun *puŋku (the Proto-Numic form) or similar, which functions as an independent noun meaning 'pet, domestic animal' and is also the possessive classifier for animals. In Chemehuevi (utes1238) and Southern Paiute of the Southern Numic subbranch, the word is an appositive classifier, taking a cliticized pronominal possessor and compounding with the possessed noun:

(S17) Chemehuevi: *nyyni* *tuku-pungku-n*
1sg.GEN mountain.lion/cat-pet-1sg
'my mountain lion/cat' (Press 1980:60)

(S18) Southern Paiute: *qava:+vungqu-ni*
horse+pet-1sg
'my horse' (Sapir 1930:121)

Languages to the north of these use exclusively dependent-marked possession, or pronominal argument marking in which pronoun possessors cliticize to possessed nouns while nouns take the genitive case. In these languages, the *puŋku reflex and the putative classifier form a compound, e.g. Tümpisa Shoshone (Dayley 1989:189):

(S19) *nia-ng* *[wasüppi* *pungku]*
1sg-GEN mountain sheep pet
'It's my mountain sheep'
(structure: my [mountain.sheep pet], i.e. [my [pet mountain.sheep]])

(S20) *nia-n* *[nütsütü* *pungku]*
1sg.GEN airplane pet
'It's my airplane'[1]

Similarly in Eastern Shoshone (shos1248, Shaul 2012):

(S21) *goso* *vungku*
pig pet
'pig, domesticated pig'

(S22) *wovim* *bungku*
log pet
'wagon' (lit. 'wood[en vehicle')

Such compounds are listed in dictionaries: Eastern Shoshone *goso vungku* (pig pet) 'pig, domesticated pig' (Shaul 2012); Northern Paiute (nort2954) *sadyʔy+puku* (dog+*puku*) 'pet dog' (Liljeblad et al. 2012). In the entries they are cited without possessors, which would not be possible if they were possessive appositives (because then they would be inalienable nouns).

In the Eastern Shoshone examples above, the initial *p- of the classifier undergoes lenition, showing that it forms a close phrase or compound with the possessed noun.[2] In Northern Paiute, the word means 'horse' or 'dog as an independent noun and appears as second element in compounds referring to domestic animals: (*sadyʔy+puku* [dog+*puku*] 'pet dog', Liljeblad et al. 2012 s.v.).

In Tümpisa Shoshone (pana1305) predicative possession, *punku* is incorporated into the verb 'have':

(S23) *Satü* *yühmü* *pungku+pa'e*
DEM porcupine pet+have
'He has a pet porcupine' (Dayley 1989 s.v. *pungku*)

as are other head nouns:

(S24) *Nü* *petü+ppa'i*
1sg daughter+have
'I have a daughter' (Dayley 1989 s.v. *-pa'in*)

The word can also be used as an independent noun meaning 'pet, domestic animal'. There is also a compound *isa+punku* 'dog' (coyote+pet).

---

[1] In Numic languages the word means not only 'dog; horse' but (in a semantic extension from ''horse') 'vehicle, means of transport'.
[2] Dayley's Tümpisa Shoshone practical orthography is at a more abstract level and does not indicate whether (S19-S20) are to be read as non-compound phrases, with [p], or as second elements of compounds, with [β]. Shaul's spelling is unambiguous.

In these Numic languages, then, the possessed noun and the *puŋku reflex form a compound or close phrase, and the *puŋku reflex is an ordinary alienable noun. Since these languages use a genitive case in some or all possessive phrases, and the cognate to the possessive appositive of sister languages with head-marked possession is appositive but not a possessive appositive, they are consistent with the generalization that appositive possessives occur only in head-marked phrases.

Farther south in the Numic branch, Chemehuevi, the southernmost Southern Numic language, has a two-classifier appositive system, using the inalienable noun -*pungku* in appositive constructions and incorporation (Press 1980:60):

(S25)	*nyyni*	*tuku-pungku-n*
	1sg.GEN	mountain.lion/cat-pet-1sg
	'my mountain lion/cat'	

(S26)	*nyy-k*	*waha-ku-my*	*waʔarovi-my*	*pungku-wy-ga-nt*
	1sg-K	two-OBL-ANIM.OBL	horse-PL.OBL	pet-PL-have-PCP
	'I have two horses'		(K = 3rd person inanimate)	

and -*ygapy* 'plant (not wild)' for plants (115; no examples given).

Southern Paiute, a close sister of Chemehuevi, has only -*pungqu* 'domesticated animal, owned animal; horse, dog', which can be used by itself or as an appositive classifier (Sapir 1930:121):

(S27) *qava:+vungqu-ni*
horse-pet-1sg
'my horse'

(S28) *pungqu-ni*
pet-1sg
'my horse'

Qava 'horse' is alienable. We assume that *pungqu-* is probably inalienable when used by itself.

In all of the Numic languages the **puŋku* reflex forms a compound or close phrase with the possessed noun, unlike most appositive possessive words. It can be analyzed as a construct morpheme, one that registers the presence of a possessor (or the fact of possession) on the head. Unlike most construct morphemes, it also functions as an independent word. Given that the possessive phrases using it are spelled and pronounced as compounds, it is not an inflectional morpheme but derivational.

There is no discussion of whether words for domesticated animals and plants are non-possessible in these languages. There are examples showing that they are possessible, without the appositive, in other Numic languages, e.g. Tümpisa Shoshone:

(S29)	*Satümmü*	*tammi*	*putisiha*	*innüntükkanna*
	those	INCL-GEN	burro-ACC	steal
	'They stole our burros' (Dayley 1989:190)			

Tübatulabal (tuba1278), an NUA isolate branch, has both possessive affixes and a genitive case. It has obligatorily possessed inalienables (kin terms, some body parts, and a few special terms) and non-possessibles (proper names and some plant and animal names), but no information is given about possession of non-possessibles (Voegelin 1935:140). There is a cognate to *puŋku in

the verb *punggu?-* 'domesticate' and the (evidently suffixed) noun *pukubiš-t* 'dog' (Voegelin 1958 s.vv.) but no reported appositive classifier.

Hopi, another isolate NUA branch, has what appears to be a fossil appositive construction using the cognate to the Numic and Tübatulabal form. Most possessed nouns, including names of commonly possessed animals, take the regular prefixal possessive markers. Names of animals not usually possessed compound the noun with *pooko,* cognate to *puŋku:

(S30) *mosa* 'cat' *i-mosa* 'my cat'
kwaayo 'hawk' *i-kway-vooko* 'my (captive) hawk'
(Hopi Dictionary Project 1998: 873–874)

In the other Northern Uto-Aztecan languages we surveyed, Cupeño, Cahuilla, and Luiseño (luis1253, Cupan branch) use head-marked possession and have possessive appositives:

(S31) Cupeño *awal ne-ʔash*
dog 1sg-CL:pet
'my dog' (Hill 2014:129)

(S32) Cahuilla *né-ʔaš ʔáwal*
1sg-own dog
'my dog' (Seiler 1995:219)

(S33) Luiseño *no-ʔa:š awá:l*
1sg-CL:pet dog
'my dog' (Kroeber and Grace 1960:82)[3]

Languages of the SUA Tarahumara-Guarijio branch (northern Mexico) have appositives of different types. The Choguitá Rarámuri (lowl1265) variety of Central Tarahumara (Caballero in prep.) has neither genitive case nor person marking on the possessed noun, but uses PSD suffixes to register a possessor. The suffix *-la* (henceforth we cite examples in the more surface of Caballero's two levels) is found on both inalienables (where it is obligatory) and alienables. Some nouns can add a second suffix *-wa* before *-la*.

(S34) a *ne* *wasá-**la***
1sg.NOM cultivation.land-PSD

b *ne* *wasá-**wa-la***
1sg.NOM field-PSD-PSD
both 'my cultivation land'

The second suffix is standard for some nouns, impossible for some, and optional for many as in these examples. Where it is optional, for the most part there is no detectable semantic difference.

Some inalienables add a third suffix, the locative *-či* (added without regard to the syntactic function, i.e. not in a locative context):

[3] Kroeber and Grace do not write the initial glottal stop; Elliott (1999) does write it in similar examples. We have therefore added the glottal stop where it is lexeme-initial but word-internal, as Hill writes it (Seiler writes it even word-initially).

(S35)	*nehé*	*kawí-**wa-la-či***
	1sg.NOM	hill-PSD-PSD-LOC
	'my hill'	

Another possessive construction uses an appositive clause with the possessive noun *nía*, which takes the PSD suffix:

(S36)	*kúmi*	*bu'í*	*ne*	*nía-**la***	*wičá*
	where	lie.sg	1sg.NOM	POSS-PSD	needle
	Where is my needle?				

(S37)	*na*	*ko*	*ne*	*nía-**la***	*líbro*	*ko*
	PROX	EMPH	1sg	POSS-PSD	book	EMPH
	'This here is my book'					

Exs. (S38a–b) show *-la* present on an inalienable (where it is obligatory) and absent from the same word in its alienable sense (which when possessed requires *nía*):

(S38a)	*nehé*	*sa'pá-**la***
	1sg	meat-PSD
	'my flesh (of my body)'	

(S38b)	*nehé*	*nía-**la***	*sa'pá*
	1sg	POSS-PSD	meat
	'It is mine the meat (to eat)' [We assume also NP 'my meat' (i.e. to eat)]		

The possessive noun can also funtion as a verb in possessive predication, glossable as 'have'. This is the only appositive noun in Choguita Rarámuri.

Rarámuri as reported by Rosenthal (1981) has two classificatory possessive verbs, one for ownership of animals: *buku-,* cognate to the Numic 'pet' classifier, in Rarámuri a verb meaning 'own (of animal)' (Rosenthal 1981:196, without source but evidently Brambila 1953).

(S39)	*nihe*	*buku-ra*	*gawe*
	1sg	pet-PSD	horse
	'my horse'		

The remaining surveyed UA languages have classifier systems with non-possessibles (or apparent non-possessibles). In the SUA Tepiman branch (which extends from central Mexico to southern Arizona), Southeastern Tepehuan (sout2976, Willett 1991) uses head-marked possession with person-number markers that are proclitic to the noun (suffix for 3sg) or enclitic to a preposed article. It is obligatory with inalienables (kin terms, body parts, meronyms).

(S40)	*gu - ñ*	*moʔ*
	ART-1sg	head
	'my head'	(82)

There is a PSD suffix *-ga- / -ʔ-* used on words for domestic animals, affinal kin terms, plant and animal parts, and some basic possessed items such as 'house', when they are possessed (Willett 1991:211)

(S41)	*gu-m*	*junu-ʔ*
	ART-2sg	corn-PSD
	'your corn'	

(S42)	*gu-m*	*<ca>cvai-ʔ*
	ART-2sg	<RED>horse-PSD
	'...your horses'	[extracted from clause]

(S43)	*gu*	*joñ-ga-ʔn*	*gu*	*Macario*
	ART	wife-PSD-3sg	ART	M.
	'Macario's wife'		[extracted]	

(S44)	*upsu-ga-ʔn*	*kyʔn*	*ty-kykyʔ*	*gu*	*nacsyr*
	stinger-PSD-3sg	with	EXTENT-bite[4]	ART	scorpion
	'The scorpion stings with its stinger'				

(S45)	*Jum-xychuiʔ*	*gu*	*cúpar-ga-ʔn*	*gu-ñ*	*jóco-ʔ*
	RFL-break	ART	top-PSD-3sg	ART-1sg	flashlight
	'The top to my flashlight is broken.'				

As can be seen by scanning the above examples, the nouns that take *-ga / -ʔ* include both words that are cross-linguistically often inalienable (kin terms, body and plant parts, other parts) and frequent non-possessibles (domestic animals, food plant parts). With the typical non-possessibles it has PSD function, but with the inalienables it is pleonastic and divides semantic inalienables into two sets: obligatorily possessed kin terms and human body parts, and affinal terms and non-human parts. The suffix then marks, approximately, things that can be acquired (Willett 1991:211).

Other Tepiman languages have appositive possessive nouns which classify nouns as animate vs. inanimate, and which take suffixes that add further classification. Seiler (1985) reports these two: Tohono O'odham (toho1245, formerly Papago; Saxton 1982:182) has animate *šoi-* and inanimate *ïñ-*, with suffixes *-ka* (possessed plant parts), *-g* (loanwords), *-ga* (others). Northern Tepehuan (nort2959, Bascom 1982:213) has animate *šoi-ga* and inanimate *tʸulʸdʸa-ga*. These then add possessive suffixes. The suffixes *-ga* are cognate to the *-ga* allomorph of the Southeastern Tepehuan PSD suffix (above).

The Cupan branch of NUA provides evidence for evolution of classifier systems, specifically for rapid growth of a small system into a large and open one. Luiseño (southwestern California) has an appositive classifier *-ʔash* for names of animals, which are non-possessible (Kroeber and Grace 1960: 82-83):[5]

(S46)	*awá:l*	'dog'	*no-a:š awá:l*	'my dog'
	no:ta	'gopher'	*no-a:š no:ta*	'my (pet) gopher'

The word also functions as an independent noun *ʔash-la* (with NPN suffix) 'pet, domesticated animal, horse, cow; shaman's familiar', which can also be possessed: *po-ʔásh* 'his horse', 'her cow', etc. (Elliott 1999:135). It also forms a suffixed denominal verb *ʔásh-lu* 'breed (livestock), increase (one's herd)'.

Closely related Cupeño (Arizona; Hill 2014) has a cognate classifier for animals and another for inanimate possessions (examples repeated from above):

[4] EXTENT is a preverb, apparently directional or spatial.

[5] Kroeber and Grace do not write the initial glottal stop; Elliott (1999) (cited just below) does.

(S47) *awal ne-ʔash*
dog 1sg-CL:pet
'my dog' (129)

(S48) *wi-lʸ ne-mixan*
lard-NPN 1sg-CL:possession
'my lard' (cooking lard) (176, 177)

Cahuilla, a neighbor and closest sister of Cupeño, has cognate nouns in the same two functions, as well as additional semantically based classifiers (Seiler 1995; 1977:298ff.). All but one can be inflected and used as either nouns or verbs, and most are basic verbs as shown by the fact that in their appositive use they take the nominalizer *-ʔa.* (For this suffix see Seiler 1977:298 and Hill 2014:316-319. We use Hill's term *nominalizer.*) Those with the nominalizer are surface nouns in apposition, but basic verbs. There are two animate classifiers, generic *-ʔaš* for domestic animals and pets, and *-kil´iw* 'totem' for the two moiety animals. The first of these appears to be a flexible noun-verb, as it can function as appositive without a nominalizing suffix; the second was nearly obsolete at the time of Seiler's fieldwork and only known as a noun.

(S49a) *né-ʔaš ʔáwal*
1sg-own dog
'my dog' (Seiler 1995:219)

(S49b) *pe-n-áš-qal*
3sg.O-1sg-own-DUR
'I own it (as a pet)' (1995:219, 1977:305)

There is a generic inanimate classifier for possessions of all kinds based on a verb root that Seiler glosses 'do' (with INDEF prefix it gives the meaning 'do (in some way)'):

(S50) *qáwiš ne-m-éxan-ʔa*
rock 1sg-INDEF-do-NZ
'my rock' (1977:300)

(The Cahuilla word takes nominalizer *-ʔa* when used appositively; the Cupeño examples lack it. For these and other inanimate classifiers Seiler includes the *-ʔa* in the citation form.)

For edible plants and/or their fruit there are classifiers based on traditional relations of ownership and usufruct. All take the nominalizer *-ʔa*.

-kiʔiw-ʔa Group of plants (or the location where they grow), and their fruit (mesquite, oak/acorn, pinyon, chia), from the verb root 'wait'.

(S51a) *ne-kiʔiw-ʔa méñikiš*
1sg-wait-NZ mesquite.beans
'my mesquite beans' (1995:221; see also 1977:301)

(S51b) *pe-n-kíʔiw-qal*
3sg-1sg-wait-DUR
'I am waiting for it'

Such places were assigned to clan lineages; "[t]he members of the lineage had a legal claim and were allowed to harvest these places when the appropriate time came" (301).

-ʔay-ʔa Fresh fruit or blossoms picked from the plant: fresh mesquite beans (in the pod), squash blossoms, corn.

(S52a) *ne-ʔáy-ʔa* *méñikiš*
1sg-pick-NZ mesquite.beans
'my mesquite beans (fresh, on the tree)' (221)

(S52b) *pe-n-ʔáy-qal*
3sg-1sg-pick-DUR
'I am picking it'

-či-ʔa Edible items after they have fallen off the plant and are picked up from the ground.

(S53a) *ne-čí-ʔa* *méñikiš*
1sg-gather-NZ mesquite.beans
'my mesquite beans (picked up from the ground)' (221)

(S53b) *pe-n-číči-qal*
3sg-1sg-gather-DUR
'I am picking it up from the ground'

-wés-ʔa Crops planted in a row.

(S54a) *ne-wés-ʔa*
1sg-plant-NZ
'my planting' (1977:303) (no adnominal example given)

(S54b) *pe-n-wés-qal*
3sg-1sg-plant-DUR
'I am planting it' (ibid.)

These classifiers are primary verbs and take the nominalizer to function adnominally. Their meanings as verbs are human actions: 'own a claim (and harvest when ripe)', 'pick, harvest', 'gather', 'plant'. In their adnominal uses they also appear to carry nominal-like quantitative or distributional implications: patch of land where edible plants grow; scattered over the ground; ripe for picking, or fresh; in a row. We assume these are aspects of what is lexicalized in the nominalized form and not inherent in the verb meaning.

There are other classifiers for other food products, based on how they are prepared: *-sex-ʔa* 'cooked', *-waʔ* 'roasted', *-čáxni-ʔa* 'melted', *-téneq-ʔa* 'barbecued':

(S55a) *né-waʔ* *túkut*
1sg-roasted wildcat
'my roasted bobcat meat' (304)

(S55b) *pe-n-wáwa-qal*
3sg-1sg-roast:RED-DUR
'I am roasting it' (304)

(S56a) *ne-čáxni-ʔa wíl´*
1sg-melt-NZ lard (305)
'my melted lard'

(S56b) *pe-n-čáxni-qal*
3sg-1sg-melt-DUR
'I am melting it' (305)

-*waʔ* 'roast' does not take the nominalizer and must be a basic noun or flexible noun-verb; the others take it and are basic verbs. All have verbal meanings based on the type of preparation. They appear not to have the quantitative or distributional implications of the edible plant classifiers.

The Cahuilla system is the only large system we have found in Uto-Aztecan. The Cahuilla classifiers use morphology and syntax that are easily available for one type of relativization, which is morphologically minimal and apparently appositive rather than subordinating, as in this example from Cupeño:

(S57) *pe-pulinma pe-ʔayew-ʔa*
3sg-man's.child 3sg-like-NZ[6]
'his son whom he loved' (Hill 2014:317)

Such constructions could easily have been coined to create possessed NPs for owned non-possessible head nouns. Thus we can reconstruct a pre-Cahuilla system in which animal nouns and some inanimates were non-possessible, or perhaps all alienables were non-possessible. Owned non-possessibles could form the appositive construction as needed, and these have been lexicalized for the salient owned or apportioned categories of animals and food items. An open question is whether the foodstuff nouns were strictly grammatically non-possessible: perhaps the possession marked by possessive affixes is semantically a more intimate or inherent relationship that does not apply outside of kin terms, body parts, or certain other closely connected nouns (e.g. a basket which the owner has made, which is inflected as inalienable in Cahuilla); or perhaps notions like 'have', ownership, and possession in Cahuilla and pre-Cahuilla necessarily involve relations of ownership or actions of claiming and are therefore naturally conveyed by verbs. (See Seiler 1977:298–307 and 1995 for a close analysis of the semantics of possession in Cahuilla.)

All three Cupan languages have the appositive classifier *ʔaash 'pet, owned animal', which Rosenthal 1981:198 describes as similar to a 'pet' classifier of the neighboring Yuman language family. She does not give the Yuman form and we have not found it in our survey (§3.3 below); we have found only Tiipay (kumi1248) -*xaṭ*, Hualapai (hava1248) -*hadh.*

The range of Uto-Aztecan appositive possessive systems can be accounted for with two principles: (1) independent elaboration in Cahuilla, using verbs in a relative construction that was generally available and could easily have become standard for certain semantic kinds of possession; and (2) tapering off to both north and south of smaller systems with non-possessible animal names and some inanimates attested in the centrally located languages.[7] In the elaborate Cahuilla system and the simpler Cupeño type from which it evolved, the classificatory appositives are verbs in origin. To judge from the Tübatulabal cognate, the Numic 'pet' classifier may have been a verb in origin.

[6] Hill interlinearizes the suffix as PSD, but as this is a type of nominalization we interlinearize it functionally in this example as NZ. It is a single morpheme with those two functions.
[7] Unless the allomorph -*ʔ* of the Southeastern Tepehuan PSD suffix -*ga- / -ʔ-* is cognate to the Cupan nominalizer and is a remnant of a once larger or more open system.

S3.2.3. Washo

Washo (wash1253, isolate, northeastern California and western Nevada; Jacobsen 1964:391–392, 408, 468)has person-number possessive prefixes. Alienables may take prefixes; inalienables, mostly kin terms and body parts, are obligatorily possessed. (Inalienables fall into three morphologically defined subclasses: see Jacobsen's Appendix 3.) Animal and plant names cannot take possessive prefixes but use appositive classifiers: *gúšuʔ* 'pet' for animals and another word for plants (Washo term not given). Other non-possessibles can add the attributive-instrumental prefix when preceded by a noun possessor. We analyze this prefix as PSD. It registers a possessor but does not index it.

(S58)	*t'á:gɨm*	'pine nuts'	vs.	*dabóʔo*	*ʔit-t'á:gɨm*
				white.man	PSD-pinenuts
				'white man's nuts' (i.e. purchased nuts)	

S3.2.4 Yuman[8]

The Yuman family, spread across southern California and Arizona and Baja California, is probably somewhat over 2000 years old. We have surveyed five of its 10 daughters, including representatives of all major branches. The languages typically have a system of three possessive classes. Inalienables directly take possessive prefixes and are obligatorily possessed; they include kin terms, body parts and plant parts, and a few others. (Kin terms and body parts have slightly different allomorphs of their possessive prefixes in some of the languages.) Alienables are optionally possessed, and when possessed some or all of them take a PSD prefix following the person prefix. A possessor noun or pronoun precedes the possessed noun. These two classes have the same structure across the family; the PSD prefix has two allomorphs, contrasting in some languages and leveled in favor of one or the other in most.

Certain other nouns are apparently non-possessible (though never so described in grammars) and take postposed appositive possessive nouns that have some classification, most often 'pet' and some kinds of inanimates, but occasionally others. These are less consistent across the family.

Examples of the three classes from Hualapai (Pai branch; Watahomigie et al. 1982):[9]

Inalienable:

(S59)	*nya*	*ʔ-jídha*	*ma*	*m-jídha*
	I	1-mother	you	2-mother
	'my mother'		'your mother' (173)	

Alienable: In Hualapai the PSD form is used chiefly for nouns referring to real property (land, houses, etc.) (187). Most nouns can use the general appositive construction (below).

(S60)	*nya*	*nyi-ʔ-wa:-v-ch*	*hánkyu*	
	1sg	PSD-1-house-DEM-SUBJECT	good	
	This house of mine is good.			(188)

The PSD prefix is also used on the animal classifier (just below).

---

8 In Yuman examples we have replaced "ɗ" (with strikethrough) with *dh.* We linearize superscript "y" and "w".

9 This grammar is notable for including sizable lists of nouns in each class and mentioning nouns that can take more than one kind of possessive marking (with discussion of the semantic difference).

Appositive: Names for some domesticated animals and some wild animals in captivity use the animal classifier *-hadh*:

(S61) *waksí ma m-nyi-hádha*
cow you 2-PSD-pet
'your cow' (extracted from clause example) (179)

Other nouns use the general classifier *-wi:-*.[10]

(S62) *madh ma m-wi:-ny-ch hánkyu*
land 2sg 2-POSS-DEM-SUBJECT good
The land that belongs to you is good. (189)

Examples from Jamul Tiipay (Delta-California branch; Miller 2001):[11]

Inalienables:

(S63a) *Ø-shally* (1-hand) 'my hand, arm'
me-shally (2-hand) 'you hand, arm'
Ø-shally (3-hand) 'his/her hand, arm' (146)

(S63b) *Ø-ntaly* (1-mother) 'my mother'
me-ntaly (2-mother) 'your mother'
kwe-ntaly (3-mother) 'his/her mother' (147)

Note the allomorphy of third person prefixes, with Ø- for body parts and *kwe-* for kin terms.

Alienables:

(S64) *Ø-nye-wa* (1-PSD-house) 'my house'
me-nye-wa (2-PSD-house) 'your house'
Ø-nye-wa (3-PSD-house) 'his/her house' (147)

Appositive possessives are not explicitly discussed, but Langdon (1970:170) has this one among her examples of other appositional NPs:[12]

(S65) *ʔa:ṣa: ʔ-ny-xaṭ*
bird 1-PSD-pet
'my pet bird'

For Maricopa (mari1440, River branch) Gordon (1986:33) describes two appositive classifiers: *ny-hat* (PSD-dog) for animals and *ny-wish* for others:

---

[10] The prefixed alienable construction is an alternative. It implies a closer or more immediate, ongoing, possessive relation compared to the appositive one (Watahomigie et al. 188–189).
[11] We have added "Ø" and interlinearized it where there is no overt prefix.
[12] Retranscribed without predictable schwas, to match Miller's transcription.

(S66) *qwaqt '-ny-hat*
horse 1-PSD-dog
'my horse'

(S67) *m-ntay posh ny-hat*
2-mother cat PSD-dog
'your mother's cat'

(S68) *kwar'o m-nywish*
knife 2-PSD.POSS
'your knife'

In Cocopa (coco1261, Delta-California branch; Crawford 1966) inalienables are much as in Hualapai and Jamul Tiipay, except that kin terms are denominal verbs, renominalized with the nominalizer *kw-:*

(S69) *ny-šmá:l* (3-ear) 'his/her ear' (98)
kw-ny-a-cáy (NZ-PSD-3-mother)[13] 'his/her mother' (99)

Only a few alienables use the PSD prefix *ny*-:

(S70)	*máṭ*	'land, ground'	*m-ny-máṭ*<br>2-PSD-land	'your land, country'	
	wá	'house'	*Ø-ny-wá*	'my house'	
	kmí	'bag, sack'	*Ø-ny-kamí*	'his pocket'	(139)

It has an allomorph *i:*- that appears on a few body part nouns:

(S71) *Ø-i:-mí:* 'my foot, leg'
m-i:-yú 'your eye, face' (139)

Most alienable nouns use an appositive construction with a form of the verb *aʔi* 'do'.[14]

(S72) *nyqwál uʔás* 'her front dress' (front:dress 3sg-DO)
nyqwál maʔís 'your front dress' (front:dress 3sg-DO (97)

(S73) *ku:k* *Ø-aʔís* 'my coke', lit. 'I have a coke, the coke I have'
1-do/have (79)

The appositive construction uses a PSD prefix for animate possessum:

(S74) *kwramáṣ* *p-ny-aʔís* 'I have children'
?-PSD-do/have (79)

---

13 Note the homophony of *ny-*, which is PSD for alienables and kin terms but third person for most inalienables. *-a-* of 'his mother' is not originally a member of the person paradigm but an epenthetic or similar vowel, arguably reanalyzed as a person marker (Crawford, 98).
14 Crawford describes the *-s* as a distributive object plural marker, describes the entire construction as apparently nominalized, and translates some examples as finite. We take it to be an internally headed nominalization.

We found no mention of a separate animal classifier.

Kiliwa (kili1268, isolate branch, more distant from the rest; Mixco (1971:106–108) has the same treatment of inalienables, except that some take a PSD prefix -*i*-:[15]

(S75a) *ʔ - ʔíy*
1 head 'my head'

(S75b) *m - yíw*
2 eye 'your eye'

(S75c) *ʔ - i - wáʔ*
1sg-PSD-house 'my house'

(S75d) *m - i - phíʔ*
2 - PSD-nose 'your nose'

Apparently all alienables take one of three shape-based appositive classifiers: *páʔ* 'non-long', *háʔ* 'long', and *č-ʔî* 'perpendicular'. These are shape-classified verbs all meaning 'put, place'.

(S76a) *ximíʔ ʔ - páʔ*
bag 1sg-place:non-long
'my bag' (108)

(S76b) *ʔ that ʔ - háʔ*
1 dog 1-place:long
'my dog' (108)

(S76c) *naitháʔ m - č - ʔî:*
horse 2 - (place:perpendicular)
'your horse' (109)

There is no mention of an animal classifier, and the examples of 'dog' and 'horse' just above indicate that domestic animals take the regular shape-based classifiers.

To summarize, most Yuman languages treat inalienables alike: kin terms, body parts, and a few others take direct possessive affixation; only Kiliwa uses the PSD prefix with some of these. Alienables are more variable. Alienables with PSD are a default class in Jamul Tiipay but a minor class elsewhere, coexisting with appositive classifiers, and absent entirely from Kiliwa. Appositive classifiers are even more variable. The Kiliwa system is entirely different from those of the other languages (consistent with its geographical and phylogenetic separation from the rest of the family). In General Yuman (the non-Kiliwa branch of the family) the appositive classifier *haṭ* for animals and the PSD prefix *ny*- are found in all three subbranches and can be reconstructed to the General Yuman protolanguage, and the general appositive *wi:*- is found in two branches and can possibly be reconstructed for General Yuman. The only structural patterns that are pan-Yuman are the direct affixation with inalienables, which is common worldwide, and the appositives in general (though not specific ones). The appositives are described as verbs or deverbal wherever the point is mentioned (Watahomigie et al. 173, 180; Crawford 1966:97; 168, Mixco 1971:106–108). It is difficult to say whether, over time, it is PSD alienables that have been expanding at the expense of appositive ones or vice versa.

---

[15] We replace Mixco's word-internal plus signs with hyphens.

S3.2.5. Keresan

Laguna (east1472), of the small Keresan family (or more properly dialect chain) of the Pueblos, has four possessive classes (Lachler 2006:54–65): non-possessibles (natural kinds such as plant and animal species, and natural phenomena such as 'snow', 'sun'); optionally possessed nouns (including artifacts, institutions, etc. and also what Lachler terms *dealienables,* alienables derived from inalienables); body part nouns (obligatorily possessed), and kin terms (obligatorily possessed, but with different possessive prefixes from those of body parts). Non-possessibles, when owned (as with domesticated animals), take an appositive construction with the nominalized verb stem -*adyaa-she* (have.as.pet-NZ) with a possessive prefix:

(S77) *kawaayu* *k'-adyaa-she*
horse 3sg-have.as.pet-NZ
'his/her horse' (62)

Wild animal names can occur in this construction if they are pets, e.g. in a myth text:

(S78) *k'-adyaa-she* *muuk'aitra*
3sg-have.as-pet-NZ mountain lion
'his (pet) mountain lion' (63; Boas 1927:07.062)

The same construction, with the cognate possessive noun and also with animal names, occurs in closely related Acoma (west2632):

(S79) *kawâayu* *ś-adyá*
horse 1sg.pet
'my horse' (Miller 1965:147)

and Santa Ana (east1472; Davis 1964:129, unsegmented example with 'his eagle').

S3.2.6. Caddoan

In Wichita (wich1260, northern Texas to Kansas; Rood 1976:143–150), apparently no nouns can take person indexation (or any other inflection; nouns are a non-inflecting part of speech). The only possessive construction is relativization. (The exact form of relativization – argument indexation or incorporation – depends on whether the relative noun is A, S, O, or R.)

(S80a) *niye:s* *niya:wéʔekih*
{*niy-uR-waʔ-iki*}
child PPL.indef:S-POSSESSIVE-DISTRIBUTIVE-be.PLURAL
'their children' (144; extracted from clause)

(S80b) *natí:weʔesikih*
{*nat-u'r-weʔes-iki*}
PPL.1S-POSSESSIVE-dog-be.PLURAL
'my dogs' (145; extracted from clause)

POSSESSIVE (Rood's term) is a verbal morpheme, as is clear in (S80a). In (S80b) it looks as though it might be a PSD affix on the noun (incorporated together with the noun), but (S80a) shows that it is a verbal morpheme. Perhaps it is a construct affix, registering but not indexing an argument; in that case it could be glossed PSD in our sense (§2.2 of main text).

In Wichita, therefore, the entire class of nouns is non-possessible. All possessive constructions use relativization.

S3.2.7 Algonquian[16]

Arapaho (arap1274, Montana; Cowell and Moss 2008:60–68) uses prefixal person indexation with any and all nouns (animate and inanimate, alienable and inalienable), the only differences in nouns being that inalienables are obligatorily possessed and some alienables have a special possessed stem formed by adding a PSD suffix. [17]

(S81)	*híxon-o*	*ne-t-íxon-eb*	
	bone-PL	1sg-EP-bone-PSD	
	'bones'	'my bone'	(63)

(S82)	*béé3ei*	*hi-béé3ei-w*	
	owl	3poss-owl-OBV	
		'his/her owl'	(62)

With inanimate possessors, however, possessive indexation is not possible, and a relative construction is used instead:[18]

(S83)	Animate possessor:	Inanimate possessor:
	hi'-óó3	*hí-íooe-éihi-:noo-'*
	3sg-leg	3.leg-AI.PASS-II-ØS
	'his/her leg'	'its leg', lit. 'it has a leg' (e.g. a table) (64)

The relative construction does not define a class of non-possible nouns; rather, the possessor determines the construction type. (See main text §4.2.)

In addition, as in most Algonquian languages, a few nouns are non-possessible and have suppletive possessible counterparts. One of these, the possessible word for 'horse', also means 'pet' and can be used appositively in Arapaho (Cowell and Moss 2008:67):

(S84)	*ho3*	'arrow'	*wóxhoox*	'horse'
	néic	'my arrow'	*nó-toníhi'*	'my horse'
	héic	'your arrow'	*hó-toníhi'*	'your horse'
	hiníic	'his arrow'	*hí-toníh'o ~ hítoního'*	'his horse' (67)

(S85)	*nó-toníhi'*	*beníixóxko'ó'*
	1sg-pet	goat
	'my pet goat'	

(S86)	*nó-toníhi'*	*nih'óó3ouwóx*
	1sg-pet	pig
	'my pet pig'	(67)

---

[16] We thank Richard Rhodes for consultation on Algonquian.

[17] Transcription is in orthography; 3 = theta. Abbreviations: EP epenthetic; OBV obviative (case); AI animate intransitive, II intransitive inanimate; Ø inanimate; S subject.

[18] With verbs of the AI and II stem classes, relative and main clauses are identical (Cowell and Moss, 373).

Suppletive pairs are discussed in grammars of most Algonquian languages, e.g. Menomini (meno1252, Bloomfield 1962:42):

(S87) *anè:m* 'dog' (non-possessible)
ne-t-i:hsèh 'my dog'
1sg-EP-dog

Appositive treatment of non-possessibles seems not to be attested outside of Arapaho (where it seems to be a contact phenomenon: see main text §4.2).

S3.2.8 The U.S. Southeast[19]

Examples are attested from several of the diverse languages of the southeastern U.S, including one verbal appositive.

S3.2.8.1. Muskogean

The only case from the Muskogean family (Florida, Georgia, Alabama) that we have found is Creek (cree1270), originally spoken in northwestern Florida and nearby southwestern Alabama (now also Oklahoma). Creek has obligatorily possessed and optionally possessed nouns, and an additional set that, while not strictly non-possessible, "sound awkward with a possessive prefix" (Martin 2011:138). These include compounds, nominalizations, and nouns that are not ordinarily possessed. Instead they take an appositive construction with the noun 'thing':

(S88) *toɫ-sakká:ka* *ca-nâ:ki*
eye-sitting.in.it 1S.PAT-thing
'my glasses' (Martin 2011:138)

The same construction can be used with optionally possessed nouns as well.

S3.2.8.2. Iroquoian

In Cherokee (Virginia, Georgia), which comprises the Southern Iroquoian branch, human root nouns (i.e. chiefly kin terms; root nouns are underived basic nouns) can take possessive prefixes, but nonhuman root nouns cannot take any inflection and instead use an appositive construction with an empty or generic root and a possessive prefix indexing the person of the possessor:

(S89) *soógwíli* *agi-ajeéli*
horse 1B-POSS
'my horse' (Montgomery-Anderson 2015:134 citing Feeling 1975:17)

Montgomery-Anderson's term for this generic root is *possessive pronoun*. Kin terms are two-argument predicates that index the possessor with a Series B prefix (patient or S/O) and the kin referent with Series A (S/A).

(S90) *agi-ji* *ja-ji* *uu-ji*
1B-mother 2B-mother 3B-mother
my mother your mother her mother (145)

These can also mean 'She is my mother', 'She is your mother', 'She is her mother', functioning as predicates.

---

[19] We thank Jack Martin and Aaron Broadwell for consultation on Muskogean and Cherokee (cher1273).

Though we have not seen appositive possession discussed in grammars of other Iroquoian languages, in fact all of the languages have non-inflectable words including some animal names, and these when owned can take an appositive construction using a possessible noun (in Northern Iroquoian typically *-tshenv* or *-itshenv* 'domestic animal') (Marianne Mithun, pers. comm.). The Iroquoian system is distinctive in that it involves general non-inflectability of nouns and not just specifically non-possessibility.

S3.2.8.3. Siouan

Appositive constructions are found in two languages of the Southeastern Siouan branch, both of which went extinct without receiving full descriptions. Biloxi (bilo1248, Einaudi 1976) has three possessive classes: obligatorily possessed (kin terms, body parts), optionally possessed (a few nouns denoting intimate possessions such as 'house' and items of clothing, which are treated as inalienables when owned), and all other nouns, which cannot be inflected for person. How ownership of these non-possessibles was expressed is unknown.

In Tutelo (tute1247) of the same branch (Oliverio 1996) inalienables are obligatorily possessed (kin terms, body parts) and are often but not necessarily used with possessive prefixes. Alienables are optionally possessed and take a longer possessive marker consisting of the person prefix plus a PSD prefix *-ta:-*. A handful of nouns also have an alternative construction with an appositive word consisting of the verb 'belong to' plus an indefinite suffix that marks it as a noun:

(S91) *wi - ta: - ku:čka:-i*
1sgP-PSD-child-INDEF
'my child' (140) (prefixal)

(S92) *wakučka* *wį - kítǫ - wi*
child 1sgDAT-belong-INDEF
'my child' (140) (appositive)

The word 'dog', and no other noun, takes a verbal appositive word 'own', which we interpret as a (finite) relative (relative clauses are not described for Tutelo). If the use with only 'dog' is not an accident of under-documentation, this is our only example of a noun lexically governing a possessive appositive word.

(S93) *čhǫk* *o - wa - hkí^{n}phi*
dog LOC-1sgA-own
'my dog' (lit. 'the dog I own')

(S94) *čhǫk* *o - ya - hkí^{n}phi*
dog LOC-2A-own
'your dog' ('the dog you own')

Non-possessible nouns are also found in the Dakotan subgroup of the Mississippi Valley Siouan branch of the northern Great Plains. Dakota ordinarily uses external rather than adnominal possession, marking the possessor on the verb with a person-number object index plus a dedicated prefix marking its possessive function (Boas and Deloria 1941:86–92, 128–130; Mithun 2001). Some nouns are non-possessible: "According to Dakota concepts certain objects, particularly natural objects and food, cannot be personal property" (Boas and Deloria 1941:90), and for those nouns the verb cannot take the possessive form and is reflexive instead. (Adnominal possessive

constructions do exist in Dakota, but we have not found a discussion of adnominal treatment of owned non-possessibles.) The Dakota material is important, as it shows that non-possessibility is independent of adnominal possessive morphology and is a more general lexical property of nouns themselves. We also surveyed Crow (Graczyk 2007), for which there is no mention of non-possessibles.

S3.3. Central America

S3.3.1. Seri (seri1257, isolate, northern Mexico; Marlett 1981)
Seri inalienables (kin terms, body parts) take possessive prefixes that index person. Alienables do not take possessive prefixes but use a verbal appositive, a relative clause with the verb 'own' in a nominalized form:

(S95)	*simalón*	*kiʔ*	*tro:ki*	*ya:*	*kiʔ*
	Cimalon	the	car	3.NMZ.own	the

'Cimalon's car' (69; extracted from clause)

(S96)	*kanóatax*	*ʔi-o-ya:t*	*koi*
	boats	1-NMZ-own	the

'our boats' (70; extracted from clause)

S3.3.2. Mayan
The Mayan family consists of some 40 languages mostly spoken in Guatemala. Mora-Marín (2021) surveys possession across the entire family. All Mayan languages use head-marked possession using person-number prefixes, and most have a variety of different types based on obligatory possession, possessibility with restrictions, non-possessibility, animate vs. inanimate possessor, intimacy of possession, and other distinctions, some of these marked by PSD and NPN suffixes of various types. One of these is what Dayley (1985:145) describing Tz'utujil (tzut1248) calls *abnormal possession:* a noun such as a body part or meronym takes unmediated possessive inflection in its alienable sense but requires a special suffix in the inalienable sense:

(S97) *kik'* 'blood': *nuu-kiik'* 'my blood' (e.g. to make sausage with)
n-kik'-eel 'my blood' (in my body)

Mora Marín shows that these suffixed forms can be analyzed as derivation, specifically abstractivization, and not as inflection.

Only a few Mayan languages have an appositive construction for non-possessibles, e.g. some natural phenomena (Mora Marín 29 and pers. comm.):

(S98)	K'ichee'	*qa-naan jab*	'our mother rain'	(1PL-mother rain)
		qa-naan uleew	'our mother earth'	(1PL-mother earth)
		qa-taat q'iij	'our father sun'	(1PL-father sun)
(S99)	Mam	*q-txuu jb'aal*	'our mother rain'	(1PL-mother rain)
		q-man q'iij	'our father sun'	(1PL-father sun)
		q-txu xjaaw	'our mother moon'	(1PL-mother moon)

or animals and food:

(S100a) Yucatec *inw-aalak'* *pèek'*
1sg-CL:animal dog
'my (pet) dog'

(S100b) *inw-ó'och* *b'u'ul*
1sg-CL:food beans
'my beans (ready to eat)'

The Yucatec pattern is like the one found in several Uto-Aztecan and nearby languages, with appositive classifiers for animals and plant food. The examples from K'ichee' and Mam are different from any we have found elsewhere, classifying (if that is the right term) nouns not by their function or the way they were acquired or prepared but by what is probably a cultural or mythic connection or metaphor. Here too, an inalienable noun serves as classifier.

S3.3.3. Otomanguean

S3.3.1. Chichimeco Jonaz (chic1272, Otopamean branch of Otomanguean; central Mexico; Lastra de Suarez 1984) has a large number of inalienable possessive classes defined by stem changes in the noun when they take possessive prefixes. These nouns are mostly kin terms, body parts, and items of clothing. Alienables do not take possessive prefixes or the internal possessive marking, but occur with one of four appositive classifiers, which are inalienable nouns and do take stem changes. Examples with singular possessors (25):

		1sg	2sg	3sg
(S101)	CL:food	*nantʔé'*	*útʔe*	*utʔé*
	CL:clothing	*nuntʔú:*	*nírʔu:*	*nintʔú:*
	CL:animal	*námbæʔæ*	*ungwǽʔæ*	*úngwæʔæ*
	CL:thing	*námbihi*	*úngwihi*	*úm̲įhį* [20]

The noun *kúroho* 'rock' with singular possessors (25):

(S102) 1sg *námbihi kúroho*
2sg *úngwihi kúroho*
3sg *úm̲įhį kúroho*

S3.3.2. Copala Triqui (copa1247, Mixtecan branch, southern Mexico; Broadwell 2011, 2016) has obligatorily possessed inalienables, possessible alienables, and non-possessible alienables. Inalienables have a juxtaposed possessor (S103a-b), which for some nouns can optionally be a clitic (c). (The noun in (S103a-b-c) has suppletive alienable and inalienable forms.)

(S103a) *rej* *xnii*
father child:ALIENABLE
'the child's father'

(S103b) *ta'nii* *so'*
child:INALIENABLE he
'his child'

[20] The original has a small underscore centered under the "m", indicating a lenis nasal (highly nasalized approximant).

(S103c) *tani-j*
child:INALIENABLE-1sg
'my child'

A small number of alienables are non-possessible and require an appositive classifier. There are two classifiers, one for animals and one for objects made of paper:

(S104a) *daán=j* *chuvee*
CL:animal=1sg dog
my dog

(S104b) *danj* *xnii* *libroó*
CL:paper child book
'the child's book'

S3.4. South America

S3.4.1. Cariban[21]

The Cariban family is fairly widespread in northern South America. The Glottolog classification lists 42 languages in 8 branches.

Cariban languages typically distinguish obligatorily possessed (typically kin terms, body parts, and a few others), optionally possessed, and non-possessible nouns, of which the latter always include domestic animals, growing plants, and some natural phenomena. (Tavares 2005:147–149 describes the semantics of Wayana non-possessibles as pertaining to the wild or non-cultural world, while possessibles belong to human culture, and their membership can be expanded as items of national and European culture become familiar in Wayana society.) Non-possessibles whose referents are owned take an appositive possessive construction with a classifier that behaves synchronically like an obligatorily possessed noun.

The classifiers are a fairly large class in languages of different branches, and often open in the sense that any relatively generic noun can be used in the same way. Classifiers can be used as appositives, e.g. Panare (enap1235) (using classifier 'liquid' as appositive):

(S105) *kape-ya* *y-úku-nh*
coffee-in 1-CL:liquid-PSD
'in my coffee' (Payne and Payne 2013:81)

or as independent nominals:

(S106) *Asa'* *t-ïkë* *mémpa-sejpa* *këj* *Toman*
two 3UNSP-CL:animal DI.steal-FUT AN.PROX Tom
'Tom is going to steal someone's two domestic animals' (86)

Several descriptions note that the classifier constructions can be discontinuous, with the classifier detached from the possessed noun, while in some languages they form a tighter phrase with strict order and no detachability (e.g. Meira 1999 for Tiriyó). Those describing the looser appositive

[21] For readability barred “i” of some of the Cariban sources is replaced with *ï*.

syntax generally describe the construction as something other than an NP and prefer the term "generic noun" to "classifier"; we call them all classifiers regardless of the closer vs. looser structure of the phrase.

Payne and Payne (2013:81–82) list 20 such classifiers for Panare (Pemong-Panare subbranch of Venezuelan Cariban); Koehn (1994) lists 9 for Apalai (apal1257, isolate branch); Tavares 2005:144 lists 13 for Wayana (waya1269, Wayanic subbranch of Guianan Cariban); Meira lists 7 for Tiriyó (trio1238, Taranoan subbranch of Guianan); Bruno (2003:69) gives only two, 'pet' and 'food', for Waimiri (waim1253, Yawaperi branch) (without, however, claiming that this is an exhaustive list). Classifier inventories for some of these are:

(S107) Apalaí (cited with possessive prefixes)

j-o-ty	1sg-meat.type.food-PSD
y-napy-ry	INCLdu-veg/fruit.type.food-PSD
ỹ-kyry-ry	1sg-field.produce-PSD
y-kyry-ry	1sg-thing-PSD
eky	3sg-pet/domestic.animal-PSD[22]
j-uhme	1sg-nut/corn/seed
i-karimo-ry	3sg-killed.game-PSD
apwahto-ry	2sg-firewood-PSD
z-u-ru	3sg-manioc.cake/bread-PSD

(S108) Wayana

ot(ï)	animal-based food
kaimo	game [animal]
akïï	farm animal; parasite; breed
anon(u)	body painting
(w)okï	beverage
ekï	pet
kïlïï	thing
muhunu	bait
pataa	place, village
kanpë	smoked animal-based food
nepïï	soft vegetable food
neme	juicy fruit/food
ka-top	thing

(S109) Waimiri

ieky	pet
wyty	food

For Macushi (macu1259, Pemong-Panare subgroup of Venezuelan branch; Carson 1982) only one classifier is found in more recent work:

(S110) *-ekïng* pet

but Carlson and Payne (1989:103) note that an older source (Williams 1932) also has a classifier

[22] Glossing *sic.* The grammar does not identify 3sg or PSD morphemes in this word.

for food.

We have found little discussion of classifier origins in Cariban. Synchronically, all are syntactic nouns and mostly inalienables. In Panare (Payne and Payne 2013:85) and Wayana (Tavares 2005:145) a few of them are verbal in origin (in Wayana those are nominalized verbs). The only source mentioned for new classifiers in open systems is nouns (usually with fairly generic semantics).

S3.4.2. Arawak

Arawak is an old and widespread family that once covered much of northern Amazonia and several Caribbean islands. The languages typically have head-marked possessive morphology, and nouns are usually divided into possessive classes based on obligatoriness of possession and the presence of PSD suffixes when possessed and/or NPN suffixes when unpossessed. Non-possessibles are generally not mentioned in grammars and where discussed at all they are few and there is no mention of classifiers or appositives. Most Arawak languages have a system of classifiers that are not possessive but have more or less gender-like functions, appearing on various modifiers in agreement with the head noun.

Examples from Yanesha' (yahe1238) showing the various classes (Duff-Tripp 1997:30–34):

(S111) Obligatorily possessed: Person-number prefix plus noun, no suffix:

po-ʔse (3sg-brother) 'her brother'

(S112) Usually possessed; NPN when not possessed.

oñ-ets (head-NPN) 'a head' *p-oñ* (3sg-head) 'his/her head'

Optionally possessed. Animates (also some relational terms and terms for edibles), usually have PSD when possessed. Inanimates are without PSD.

(S113)	*ochec*	'dog'	
	p-ochc-ar	(3sg-dog-PSD)	'his/her dog'
	cac	'fish'	
	po-cac-ar	(3sg-fish-PSD)	'his fish; his catch of fish'
	noñt´ [23]	'canoe'	
	poʔ-noñt´	(3sg-canoe)	'his/her canoe'

Non-possessibles are chiefly *chesha'* 'child' and *huocchanesha'* 'orphan'.

When an inalienably possessed noun is additionally owned, the noun takes two possessive prefixes:

(S114)	*pa-ʔmeʔ*	*no-pa-ʔme-r*
	3sg-egg	1sg-3sg-egg-PSD
	'its egg' (i.e. the chicken's)	'my egg' (chicken's egg that I own)

[23] Duff-Tripp uses a tilde over all palatalized consonants. We write *t´*, etc. for letters we cannot place a tilde over.

Achagua (acha1250, Wilson 1992:65) has two possessive classifiers, animate and inanimate, used with alienable nouns. The classifier takes a possessive prefix if the possessor is a pronoun.

(S115)	*hi-íhiža*	*éema*	*Manuéľi*	*íhiža*	*éema*
	2sg-POSS	horse	Manuel	POSS	horse
	'your horse'		'Manuel's horse'		

(S116)	*ľi-šínaa*	*páiniya*	*wašiáľikuá-eži*	*šínaa*	*páiniya*
	3sgM-POSS	machete	man-SG	POSS	machete
	'his machete'		'the man's machete'		

Achagua also has a system of 12 numeral classifiers. The appositive classifiers above are not part of that system (Wilson's term for them is *posposición de pertenencia*.)

Piapoco (piap1246, Reinoso 1999:92–98) also has an appositive possessive classifier. Inalienables (kin terms, body parts, plant parts) are obligatorily possessed. Alienables, if owned, take a PSD suffix together with the possessive prefix. Non-possessibles cannot take possessive prefixes or the PSD suffix. When owned they can optionally take the appositive possessive noun:[24]

(S117)	*tzema*		'tobacco'
	nu-azu tzema	(1sg-POSS tobacco)	'my tobacco'

In Nanti (nant1250, Kampa branch; Michael 2008:297–301) the usual Arawak system is undergoing change. Possessive prefixes are obligatory with inalienables (which are further distinguished by being able to be incorporated into verbs, with the possessor marked by verbal person markers). Alienables take the same possessive prefixes and, optionally, the PSD suffix. The pan-Arawak NPN suffix that derives alienables from inalienables has been lost; instead, Nanti speakers use the inclusive possessive marker: *a-gito* (INCL-head) 'our.INCL head; a head'.

S3.4.3. Tupian

Tupian is an old family widespread in Amazonia and nearby. We have surveyed only the major branch, Tupi-Guarani.

Nearly all Tupi-Guarani languages distinguish inalienables (obligatorily possessed), which include kin terms, body and plant parts, and some intimate possessions such as clothing; alienables (optionally possessed); and non-possessibles, and grammars generally mention these three types and cover inalienables and alienables. The possessive marking for these is generally identical (except for the obligatory vs. optional nature), but the treatment of nouns without possessors or with unspecified possessors differs: inalienables have dedicated unspecified marking, while alienables instead use the first person inclusive for the same meaning (e.g. Guarani (para1311): Gustafson 2014:328). Just how unspecified possession of inalienables is marked varies. In Kamaiurá (kama1373, Seki 2000) and Tapiete (tapi1253, Gonzalez 2005:111) there is a dedicated UNSP possessor prefix *t-* in the paradigm of person-number prefixes; in Wayampi (waya1270) the marking is initial consonant alternations or vowel loss in the noun, e.g. *-poã* 'medicine': *e-poã* 'my medicine', *moã* 'medicine' (Jensen 1998:500).

---

[24] Wilson illustrates only with non-possessibles that when owned can be treated as either alienable (with classifier) or inalienable (with possessive prefix and no PSD), without discussion of the semantic difference. When 'tobacco' is treated inalienably the possessed form is *nu-tzema* (1sg-tobacco) 'my tobacco'.

While grammars generally identify a non-possessible class of nouns, they are less likely to discuss the marking of owned non-possessibles. Kukama-Kukamiria (coca1259, Vallejos 2016) has no person agreement but simply preposes a possessor to the head, creating an identifiable type of NP but not alienable vs. inalienable classes of nouns. Kin terms that vary for ego's gender (such as 'son' and 'daughter') have a "strong tendency" (114) to have a possessor; those without gender reference are optionally possessed. Non-possessibles would not be grammatically identifiable in such a system. Tapiete (tapi1253, Gonzalez 2005) has affixal possessive marking, which is obligatory for inalienables and optional for alienables, but perhaps no firmly grammaticalized class of non-possessibles; possessive morphology is pragmatically odd but elicitable for nouns denoting wild animals, air, earth, etc. For neither of these languages do there appear to be appositive possessive nouns.

For Paraguayan Guarani, Velasquez-Castillo (1996:9–85) shows that possessive predication is usually non-verbal for inalienables but verbal for alienables:

(S118) *(Che)* *che-memby-ta*
1sg 1sg.INACTIVE-offspring-FUT
I will have a child. (Velasquez-Castillo 66)

(S119) *A-reko* *Maria* *mesa* *che-roga-pe*
1sg.ACTIVE-have Maria table 1sg.INACTIVE-house-in
I have Maria's table at home. (76)

and equational predication of a possessed noun generally involves alienables:

(S120) *Ko-mesa* *che-mba'e*
this-table 1sg.INACTIVE-thing
This table is mine. (alienable noun) (84)

(S121) * Ko-memby che-mba'e
this-child 1sg.INACTIVE-thing
This child is mine. (inalienable noun) (84)

Her explanation relies on iconicity: less conceptual distance means less formal distance, so the inherent relationality of inalienables favors lack of an overt verbal form. See the discussion of appositives as predicative in §4.8 of the main text: we suggest that that there is an implicational hierarchy for null manifestation of a copula, and a first step in the hierarchy is predicative possession with an inalienable noun.

Some languages do have appositives, typically one for animals or two for domestic vs. wild animals. Emerillon (emer1243, Rose 2003) has only one, for domestic animals:

(S122) *de-le-iba* *zawal*
2sg-LINK-animal dog
'your dog'

Aikhenvald (2012:291) says that most Tupi-Guarani languages distinguish classifiers for domestic vs. wild animals. Only in Kamaiura, carefully described in Seki (2000), have we found a large system of appositive classifier nouns (302–303):

(S123) *-emijat* prey, game (lit. 'lo que é apanhado' = 'what's caught')
-emi'u food ('what's ingested')
-eymap domestic animal, pet
-yet canoe; vehicle, means of transport
-ywyrapat bow, weapon
-yru covering, wrap, clothing
-pyt body, house
-kyap sleeping place
-ata fire
-apo artifact, accessory

Note that these include prototypical non-possessibles such as prey and domesticated animals, and meanings that are commonly found among inalienables such as clothing, weapons, and houses. Not all of these are explicitly identified as non-possessibles, which (p. 54) are listed as including animals, plants, natural phenomena, and persons, and artifacts and tools are explicitly identified as alienable. The appositive construction, then, is broader than the non-possessible class.

Examples of the appositive construction are all translated as predicative (302):

(S124) *paku-a* *je = r - emijat*
paca-N 1sg-LINK-prey
'The paca is my prey (game animal)'

(S125) *ini -a* *je=kyap*
net-N 1sg-sleeping.place
'A net is my sleeping place'

(S126) *wararwijaw-a* *je =r -eymap*
dog -N 1sg-LINK-pet
'The dog is my pet'

Seki paraphrases *-emijat* 'game animal' and *-emi'u* 'food' as with verbal locutions: 'what is caught/hunted' and 'what is ingested' (302). We note that *-emijat* 'game animal' and *-eymap* 'domestic animal' have cognates elsewhere in the family (e.g. Jensen 1998:503) and these are the ones that Aikhenvald reports as most common in the family.

We conclude that the typical and probably reconstructable Tupi-Guarani system had appositive classifiers for game animals and domestic animals. The system has shrunk or disappeared in a few languages, and has grown considerably in Kamaiurá, where a more widely used appositive construction for equative predicative possession ('X is my Y') is used and perhaps conventionalized for owned non-possessibles.

S3.4.4. Matacoan

Matacoan is a small family found mainly in the Gran Chaco and east Andean region of Argentina.

Wichi Lhomtes (wich1261, Argentina; Nercesian 2014:167) uses direct person-number prefixation with inalienables, but with alienables it attaches the person-number index to a classifier: *ka-* for inanimates, especially instruments and tools but also others, and *lo=* for domesticated animals. *ka-* is only a prefix, but *lo=* can also be used as an independent noun 'animal'.

(S127a) *n' -ka-husan*
1sg-CL-axe
'mi hacha' (168)

(S127b) *n' -lo=mitsi*
1sg-CL=cat
'mi gato' (168)

(S127c) *la-lo=y*
3-CL=PL
'los animales' (169; extracted from clause example)

S3.4.5. Guaycuruan

Guaycuruan is a small family spoken in the Gran Chaco area of northern Argentina. Kadiweu (qomm1235, Sandalo 1995) has obligatorily possessed inalienables, which directly add a person-number prefix. [25]

(S128a) *l-Geladi*
3-house
'his house'

(S128b) *Gad:-akilo*
2-head
'your head' (56)

Alienables ordinarily have no prefix; they can be possessed, but then must have a classifier or what we gloss PSD between the person prefix and the noun root. There are three classifiers: female domestic animal, non-female domestic animal, and generic (all others).

(S129a) *l-wiGadi* *apolikGanGa*
3-CL:animal horse
'his horse'

(S129b) *l-wiqate* *apolikGanGa*
3-CL:female.animal horse
'his female horse'

(S129c) *Gad:-neb:i* *aqi:di*
2-CL:generic river
'your river'

The PSD prefix *n-* may be a reduced form of the generic classifier (Sandalo 57).

(S130) Gad:-n-aqi:di
2-PSD-river
'your river'

---

[25] Sandalo gives examples in both phonemic and more abstract transcription. We use only the more abstract one.

Two classifiers can co-occur (for emphasis: 57)

(S131)

i-wiGadi	*i-neb:i*	*apolikGanGa*
1-CL:animal	1-CL:general	horse
'this horse of mine' (57)		

There are some nouns that cannot be possessed (even with classifiers). "If they do occur with possessive markers, the meaning changes" (58). The only example given is *epenay* 'moon' : *i-n-epenay* (1-PSD-moon) 'my month' (the month I was born in).

S3.4.6. South American isolates

S3.4.6.1. Itonama (iton1250, Bolivia; Crevels 2006, 2009) uses relativizing morphology on alienably possessed nouns, but with no verb root involved in the possession. Inalienables take a possessive prefix on the noun. Kin terms are verbs, and additionally take a subordinating prefix and verbal number marking.

(S132) Inalienable:

us-nu	'my nose'	
u'-nu	'your.M nose'	
ku'-nu	'your.F nose'	(2009:250)

(S133) Kin term:

ah - may - maye'ne	*ah - may - maye'ne-'cha'ke*	
3-SUBORD-father	3-SUBORD-father-MULTIPLE:ARG.	
'his father'	'their fathers'	(2006:163)

Alienables take the regular person-number prefixes followed by the relativizer/nominalizer *mi- / ni-,* which we gloss POSS. Forms of *uku* 'house' (2009:250):

(S134)

as - mi - ku	*dih - ni - ku*	*sih - ni - ku*
1sgPOSS-house	INCL-POSS-house	1pl-POSS-house
‘my house’	‘our-INCL house’	‘our-EXCL house’

S3.4.6.2. Urarina (northern Peru lowlands; Olawsky 2006)
Modern Urarina (urar1246), under Spanish influence, has lost aspects of its former alienability opposition, but the original system is still evident in traditional language. Inalienables (kin terms, body parts, and a few others) were obligatorily possessed, with person prefixes. 3sg *n-* is now frozen in the citation form of some of these nouns (350–351):

(S135)

alaarihja	'chest'	1sg	*k=alaarihja*	3sg	*n=alaarihja*
(n)anaae	'leg'		*k=anaae*		*n=anaae*
nïlïnï	'neck'		*ka=lïnï*		*raj nïlïnï*

Alienables were non-possessible and, when owned, used an appositive construction using a nominalized form *raj* of the verb 'receive' preceded by a juxtaposed noun or independent pronoun

(S136)

akaïrï	*raj*	*batia*
3pl	POSS	pot
'their pots'	(336)	

(S137) *kača* *raj* *lureri*
man POSS house
'the man's house' (335)

The appositive word is now optional, or can take the possessive clitic of inalienables:

(S138) *kanï* *raj* *teru* ~ *kanï* *teru*
1sg POSS axe 1sg axe
'my axe' (353)

(S139) *ka=raj* *kanaanaj-ïrï=ra*
1sg=POSS child=PL=EMF (EMF = clause-final emphatic particle)
'my children' (338)

S3.4.6.3. Yurakare (yura1255, central Bolivia; van Gijn 2006) uses a person prefix for inalienables:

(S140) *ti-sibë*
1sg-house
my house

(S141) *shunñe* *a-sibë*
man 3sg-house
'the man's house' (116)

Most animal nouns cannot take the possessive prefixes but use a lexical classifier:

(S142a) *ti-talipa
1sg-chicken
'my chicken'

(S142b) *ti-tiba* *talipa*
1sg-pet chicken
'my chicken' (74)

Human nouns can only take possessive prefixes if they are kin terms or proper names (116–117; the grammar does not discuss whether non-kin human nouns can take appositives).

S3.4.6.4. Kwaza (kwaz1243, northwestern Brazil; van der Voort 2004)
Kwaza has a very large and probably open set of classifiers, nearly 150 (van der Voort 2004:177) which have a wide range of functions, one of which is possession. There is no alienability distinction. The possessor is in the nominative case, followed by either a nominalizer or a classifier. Van der Voort analyzes the nominalizer as a neutral classifier. In the following examples *-hỹ* is the nominalizer/neutral classifier and *-xy* is the classifier for such things as houses.

(S143) *'si-dy-hỹ* *a'xy*
1sg-GEN-NMZ house
'my house'

(S144) *Tawi'wi-dy-hỹ a'xy*
T.-GEN-NMZ house
'Tavivi's house'

(S145) *(a'xy) si-dy-xy*
house 1sg-GEN-CL:house
'my house'

(S146) *Tawi'wi-dy-xy*
T.-GEN-CL:house
'Tavivi's house'

These examples are from pp. 130–131; for other nouns with other classifiers see 181–186.

Since there is no alienability distinction, and since the possessor is nominalized, Kwaza can be described as a language with exclusively appositive possession using classifiers.

S3.5. The Pacific

S3.5.1. Oceanic

Most languages of the Oceanic branch of Austronesian contrast what the Austronesianist tradition calls *direct* and *indirect* possession, largely corresponding to our *inalienable* and *alienable* respectively. Lichtenberk (2018) gives a detailed survey of possessive constructions and their evolution. Lynch et al. (2002) is an earlier survey of just Oceanic. Proto-Oceanic had a small closed set: POc *ka- 'food', *m(w)a- 'drink', *na- general. These have no recoverable lexical source but may possibly be related to demonstratives (Lynch et al. 77). Whatever their sources, in modern languages they apparently are inalienable nouns in synchronic morphology, taking possessive affixes, showing that they are also to be found in the closest sister group to Oceanic, the South Halmahera-West New Guinea branch. Outside of Oceanic, Proto-South Halmahera-West New Guinea had a two-classifier system, *na- 'food' and *ri- general; the *na- classifiers of the two branches may be cognate (Lichtenberk 2018:186–190). Donohue and Schapper (2008) find other, more distantly related Austronesian languages with classifiers

With direct possession, the head noun takes a person-number possessive affix. This pattern is used on kin terms, body parts, other parts, and other prototypical inalienables. With indirect possession, the possessed noun cannot take possessive person affixes; instead, a classifier carries the person-number affixes and is syntactically in apposition to the possessed noun. Classifiers do not classify nouns rigidly; there is often freedom to choose a classifier based on the semantics. See main text §1 for Fijian examples and §2.2 for Paamese examples.

Larger inventories of possessive classifiers are found in three separate subgroups of Oceanic: Mussau (St. Matthias branch; the branch comprises Mussau (muss1246) and one very similar but nearly undescribed language); the Nuclear Micronesian branch (14 languages: Kosraean (kosr1238), Kiribati (gilb1244), Marshallese (mars1254), and 11 languages of the Pohnpeian-Chuukic subfamily); and Iaai (iaai1238, of the three-language Loyalties subgroup of New Caledonian). Since these three instances are widely separated phylogenetically and geographically, Lynch et al. reconstruct the abstract set of classifiers to Proto-Oceanic, but since it was an open class with changing membership and probably optional usage no individual classifiers can be reconstructed (79).

This larger set of classifiers again pattern as inalienable nouns, and many of them are derived from lexical nouns or verbs. E.g. Iaai (Lynch et al. 782–783; list selected from Ozanne-Rivierre 1976):

(S147) From nouns:

umwa-	houses, shelters	<	*uma* 'house'
nuu-	plants		*nu* 'coconut'
huu-	boats		*hu* 'boat'
tange-	baskets		*tang* 'basket'
mëni-	power		*mën* 'power'
waii-	reefs		*wai* 'reef'

(S148) From verbs:

hlogu-	fires	*hlök* 'warm oneself'
hicö-	things to be chewed	*hic* 'chew'
bicö-	headgear	*bicâ* 'wear on the head'
haalee-	animals	*haatr* 'look after'
döö-	spears	*dââ* 'pierce'

(S149) No known lexical source:

a-	food
bele-	drink
hnââ-	passive [undergoer of nominalized verb]
hne-	agent [of nominalized verb]
hanii	catch (of fish, game)
hwa-	noise, voice
iie-	piece for chewing
ii-	land
dee-	road, path
anyi-	general

Examples:

(S150) *wââ* *a-n* *thaan*
fish POSS:FOOD-3sg chief
'the chief's fish (to eat)' (783)

(S151) *köiö* *bele-n* *moomo*
water POSS:DRINK-3sg woman
'the woman's water (to drink)' (783)

Note also that, orthogonal to possessive classification, many Oceanic languages have a separate system of classifiers used as numeral classifiers and/or attached to the nouns themselves. These are different in function and also in semantics: "the numeral classifiers are generally based on physical form, while the possessive classifiers are generally based on function" (Lynch et al. 42).

Harrison (1988) shows that Kiribati/Gilbertese, the only Micronesian language lacking possessive classifiers, uses appositive possessed nouns and nominalized verbs in a looser construction for alienables.

(S152) *nima-u* *te* *ran*
drink-1sg ART:PSD water
'water for me to drink' (70)

The verb is an object nominalization with the semantics of a headless relative ('what I drink') (1988:74). The article is a dedicated possessive article.

This construction is optional, used only when further specification (here, the intended use of the water) is wanted, while in the other Micronesian languages a possessive classifier is obligatory with non-possessibles. Harrison argues that the Gilbertese construction is archaic and reflects the ancestral construction which evolved into the classifier construction of the sister languages. Appositive words that tend to figure in the Gilbertese construction are mostly cognate to classifiers of the other Micronesian languages.

Are the Oceanic possessive classifiers nominal or verbal in origin? In individual languages some of them are described as nominal or verbal in origin (as for Iaai above), but this is synchronic derivation or synchronically transparent etymological relatedness. Harrison reconstructs a nominalized verb form as ancestral to the construction in Micronesian and notes that many of the classifiers are cognate within Micronesian. Lynch et al. do not mention whether they can be traced back within the other two branches, stating only that no Proto-Oceanic set of classifier words is reconstructable. In any case, given the considerable part-of-speech flexibility of major class lexemes in Oceanic and more generally Austronesian languages (Foley in prep., 2017, 2008; Himmelmann 2008, and much earlier discussion; Foley reconstructs flexibility of the great majority of lexemes as the Proto-Austronesian state), etymological work tracing parts of speech of classifiers may prove inconclusive and the question itself may not be particularly meaningful. It may be telling, though, that the classifier construction must have evolved independently in Micronesian and must have been verbal in origin there.

S3.5.2. Non-Oceanic Austronesian

All are geographically distant from each other. South Halmahera-Western New Guinea is the closest sister to Oceanic. The others are from entirely different branches of Austronesian.

S3.5.2.1. South Halmahera-Western New Guinea: Donohue and Schapper (2008); s.a. Lichtenberk (2018). In Ambai (amba1265), most nouns use an appositive noun, but body parts and some kin terms take a possessive suffix directly. (There are further complications; see Donohue and Schapper 2008:320.)

(S153) Ambai (Donohue and Schapper 2008:320, citing Anceaux 1961, Silzer 1983, Price et al. n.d.)

ne-hu	*wafo*	*ne-mu*	*wiwing*
POSS-1sg	canoe	POSS-2sg	woman
'my canoe'		'your wife'	

awe-hu	*tama-mu(ai)*
leg-1sg	father-2sg
'my leg'	'your father'

In Selaru (sela1259) and Sawai (sawa1247), body parts are inalienable. Other nouns take one of two classifiers: food and general.

(S154) Selaru (Donohue and Schapper 2008:320, citing Coward 1990)

(a) *iblu-mw-ke*
skin-2sg-ART
'your skin'

(b)	*wasi-mw*	*hahy-desy-ke*
	CL:GENERAL-2sg	pig-that-ART

'your pig there', 'that pig of yours'

(c)	*hina-mw*	*hahy-desy-ke*
	CL:EDIBLE-2sg	pig-that-ART

'your pork there', 'that pork of yours'

S3.5.5.2. Others. Examples also occur in other branches: Banggai in the Celebic branch and Tugun and Kaitetu in different subbranches of Central-Eastern Malayo-Polynesian (Mark Donohue, p.c.).

S3.6. Non-Austronesian (Papuan) Pacific

Six non-Austronesian languages, phylogenetically and geographically distant from each other, have appositive classifiers. All are isolates or from small families.

S3.6.1. Abun (abun1252, isolate) (Berry and Berry 1999:77)

Inalienables (body parts and other parts of wholes) form possessive constructions with juxtaposition:

(S155)	*ndar*	*sye*	*ne*	*gwes*
	dog	big	DET	leg

'the big dog's leg' (77; extracted from clause)

(S156)	*ji*	*syim*
	1sg	arm

'my arm' (77; extracted from clause)

Alienables use an appositive word (called a linker in the grammar, following Croft (1990:32), and glossed POSS in the grammar)

(S157)	*gap*	*sye*	*ne*	*bi*	*gan*	*ge*	*we*
	rat	big	DET	POSS	young.one	CL	two

'the big rat's two young offspring' (78; extracted from clause)

(S158)	*ji*	*bi*	*nggwe*
	1sg	POSS	garden

'my garden' (78; extracted from clause)

Kin terms are alienable (78, 82):

(S159)	*Rahel*	*bi*	*ai*	*bi*	*nyom*
	Rachel	POSS	father	POSS	machete

'Rachel's father's machete' (82)

(S160) *An bi nji bi nggon bi nu*
3sg POSS brother POSS wife POSS house
'his brother's wife's house'

The Indonesian of Abun speakers calques the Abun possessive phrase, using the verb 'have' as possessive :

(S161) *saya punya rumah*
1sg have house
'my house' (81)

The fact that a verb is used suggests that the possessive morpheme *bi* of Abun is a verb (its source is not discussed in the grammar).

S3.6.2. Mpur (mpur1239, isolate) (Odé 2002)

Alienables use an appositive verb *tar* or *bi* 'possess' (61):

(S162) *n-tar jan*
1sg-POSS house
'my house' (62; extracted from clause)

(S163) *Lambert-a*[26] *a-bi ba(r)-in*
L.-3sgM 3sgM-POSS thing-grow
'Lambert's garden' (62; extracted from clause)

Inalienables (body parts, kin terms, and a few others) use a person-number prefix.

(S164) *a-wom*
3sgM-hand
'his hand'

(S165) *e-yen*
1pl-mother
'our mother'

(S166) *an-muk*
2sg-name
'your name'

S3.6.3. Bilua (buky1245, isolate) (Obata 2003:98–105)

In Bilua, inalienable nouns can take either direct (prefixal) or indirect (appositive) possession; alienables can take only appositive possession (34–35).

(S167) *ke=mama*
3pl father
'their father' (99; extracted from clause)

---

[26] The *-a* 3sgM on *Lambert* is autogender. Personal names are suffixed with *-n* (female) or *-a* (male), as are some other human nouns: 'mother', 'father', 'girl, woman', 'boy, man'; 'girlfriend', 'boyfriend' (63).

(S168) *o=baerebaere* *poso*
3sgM=friend PL.M
'his friends' (99; extracted from clause)

Inalienable nouns include kin terms, body parts, 'match', 'self', and "common nouns which have a special significance in the life and culture of Vella La Vella island people" (34–35): 'house', 'village/home', 'garden', 'island', 'friend', 'tribe'.

Indirect (alienable or inalienable; alienables can take only indirect possession) (35):

(S169) *Ilai=ko* *matu=ma* *pade*
I.=POSS.3sgF big=3sgF house (101)
'Ilai's big house'

(S170) *vo = ko* *ngi*
3sgM POSS.3sgF name
'his name' (75; extracted from clause)

ko (POSS.3sgF), the possessive word in these examples, is a 3sg distal demonstrative, *=vo* for singulative number and *=ko* for unspecified number (75; 100).

S3.6.4. Sulka (sulk1246, isolate) (Tharp 1996:80–81; Schneider 1962:259–261; Reesink 2005:179–183)

It appears that in Sulka part-whole terms and probably body parts and kin terms take direct head-marked prefixal indexation of the possessor. There are four different sets of possessor indices, for non-kin, kin, and two emphatic possessors.

(S171) a *kua-rik*
1sg-house
'my house' (80)

b *ko-nan*
1sg-mother
'my mother' (80)

c *kota-kom*
1sg.EMPH-knife
'my own knife' (81) (EMPH non-kin)

d *kot* *nan*
1sg.EMPH mother
'my own mother' (81)

Examples with overt possessors:

(S172) a *a-kom* *ka-rain*
sg-knife 3sg-handle
'the knife's handle' (118)

b *en* *ka-rai*
3sg 3sg-handle
'its handle'

Kin terms and parts of wholes, which appear in these examples, are not obligatorily possessed (118–119).

Other nouns use an uninflected form of the verb 'belong to' without argument indexation. It is not clear whether this is a prepositional or appositive construction. If appositive, it is atypical in that the putative appositive word does not bear person inflection. (It is not a relative construction, as those have relative pronouns and verbs inflected for person and TAM in the relative clause: 147–148. Schneider 1962: 259–261 considers the form a preposition. Reesink 2005:181 considers it possibly either a verb or a preposition.)

(S173) *a-kom* *to* *mkor* *e-Pruo*
sg-knife DEM.sg POSS:belong.to PN-Pruo
'Pruo's knife' (117)

(S174) *a-kom* *to* *mkor* *dok*
sg-knife DEM.sg POSS:belong.to 1sg
'my knife' (117) (PN = proper noun prefix)

S3.6.5. Bunaq (buna1278, Timor-Alor-Pantar; Donohue and Schapper 2008:322, citing Steinhauer 1977, 1991) uses prefixal possessive marking for inalienables and a single appositive word for alienables:

(S175) *n-up* *ni-e* *zap*
1-tongue 1-POSS dog
'my tongue' (inalienable) 'my dog' (inalienable)

Other languages in the family with this pattern include Blagar and Klon.

S3.6.6. Motuna (siwa1245, South Bougainville family, Bougainville Island, Papua New Guinea; Onishi 2012). Motuna has obligatorily possessed kin terms (*po-oro* [her-daughter] (89). Alienables cannot take possessive prefixes but have the option of either dependent-marked inflection or an appositive construction using one of the 51 classifiers of the language.

(S176) a *hoo* *howo* *peeko-na-pa*
ART.M house 3nonsg.POSS-LINK-CL:shelter
'their house' (in contrast to other houses) (244) (appositive)

b *peeko-ng* *howo*
3nonsgPOSS-M house
'their house' (245) (dependent-marked simple NP)

(The classifiers are used with both numerals and demonstratives, articles, etc.) There is a semantic or pragmatic difference: the appositive construction implies contrastive focus on the possessive NP or the possessive relationship. The classifiers are not independent nouns in Motuna, but they take the same possessive prefixation as the kin terms, so they are structurally like inalienable nouns.

This is the only example we have found where an appositive construction is just one option for alienables, and the choice has semantic consequences.

S3.6.7. Koiari (gras1249; Koiarian family, southern New Guinea; Dutton 1993, 1996). Animal names are non-possessible (no owned non-possessibles are reported). Dutton classifies animal names as proper names, none of which can occur in possessive constructions.

References

Aikhenvald, Alexandra Y. 2012. *The languages of the Amazon*. Oxford: Oxford University Press.

Anceaux J. C. 1961. *The linguistic situation in the islands of Yapen, Kurudu, Nau, and Miosnum, New Guinea*. The Hague: Martinus Nijhoff.

Bascom, Burton. 1982. Northern Tepehuan. In Ronald W. Langacker, (ed.), *Studies in Uto-Aztecan grammar, 3,* 271–393. Dallas-Arlington: SIL and University of Texas, Arlington.

Berry, Keith and Christine Berry. 1999. *A description of Abun: A West Papuan language of Irian Jaya*. Canberra: Australian National University.

Bloomfield, Leonard. 1962. *The Menomini language*. New Haven: Yale University Press.

Boas, Frans. 1927. *Keresan texts.* American Ethnological Society Publications 8:2 and 8:1. Leiden: Brill.

Boas, Franz & Deloria, Ella. 1941. *Dakota grammar* (23:2). Washington, DC: Government Printing Office.

Brambila, David. 1953. *Gramática rarámuri*. México: Editorial Buena Prensa.

Broadwell, George Aaron. 2011. Surrogate possession in Copala Triqui. Presented at HPSG Conference. MS, University at Albany.

Broadwell, George Aaron. 2016. La sintaxis de posesión par sustituto en trique de Copala. Presented at Coloquio sobre Lenguas Otomangues y Vecinos (COLOV). April 2016, Oaxaca, Mexico. http://www.academia.edu/24371419/La_sintaxis_de_posesion_por_sustituto_en_triqui

Bruno, Ana Carla. 2003. *Waimiri Atraoari grammar: Some phonological, morphological, and syntactic aspects.* PhD, University of Arizona.

Bugaeva, Anna. 2004. *Grammar and folklore texts of the Chitose dialect of Ainu (Idiolect of Ito Oda).* ELPR Publication Series A-045. Suita: Osaka Gakuin University.

Caballero, Gabriela. In preparation. A grammar of Choguitá Rarámuri. MS, University of California, San Diego.

Campbell, Lyle. 1985. *The Pipil Language of El Salvador*. Berlin: Mouton de Gruyter.

Carlson, Robert & Doris L. Payne. 1989. Genitive classifiers. In *Proceedings of the Fourth Meeting of the Pacific Linguistics Conference*, (ed.) Robert Carlson, Scott DeLancey, Spike Gildea, Doris L. Payne, & Anju Saxena, 87–119. Eugene: University of Oregon.

Carson, Neusa. 1982. *Phonology and morphosyntax of Macuxi (Carib).* PhD dissertation, University of Kansas.

Casad, Eugene H. 1984. Cora. In Ronald W. Langacker (eds) *Studies in Uto-Aztecan grammar, 4*, 153–459. Dallas: SIL.

Chiri, Mashiho. 1942(1973). Ainu gohoo kenkyū – karafuto hōgen o chūshin to shite [Studies in Ainu grammar – with an emphasis on the Sakhalin dialect]. In *Chiri Mashiho chosakushū* 3, 455–586. Tokyo: Heibonsha.

Chiri, Mashiho & Kyōsuke Kindaichi. 1936(1974). Ainu gohō gaisetsu [An outline of Ainu grammar]. In *Chiri Mashiho chosakushū* 4, 3–197. Tokyo: Heibonsha.

Coward, David Forrest 1990. An introduction to the grammar of Selaru. Phd dissertation, University of Texas At Arlington.

Cowell, Andrew & Alonzo Moss Sr. 2008. *The Arapaho language*. Boulder: University Press of Colorado.
Crawford, James M. 1966. *The Cocopa language.* PhD dissertation, University of California, Berkeley.
Crevels, Mily. 2006. Verbal number in Itonama. In Grażyna Rowicka & Eithne B. Carlin, eds., *What's in a verb? Studies in the verbal morphology of the languages of the Americas,* 160–170. (LOT Occasional Series, 5.) Utrecht: LOT.
Crevels, Mily. 2009. Itonama. In *Lenguas de Bolivia, II: Amazonía*, ed. Mily Crevels and Pieter C. Muysken, 233–294. La Paz: Plural.
Croft, William. 1990. *Typology and universals*. Cambridge: Cambridge University Press.
Davis, Irvine. 1964 *The language of Santa Ana Pueblo* (BAE Bulleti 191.53–190). Washington, DC: Government Printing Office.
Dayley, Jon P. 1985. *Tzutujil Grammar*. Berkeley-Los Angeles: University of California Press.
Dayley, Jon P. 1989. *Tümpisa (Panamint) Shoshone Dictionary*. Berkeley-Los Angeles: University of California Press.
Donohue, Mark & Annette Schapper. 2008. Whence the Austronesian possessive construction? *Oceanic Linguistics* 47(2). 316–327.
Duff-Tripp, Martha. 1997. *Gramática del idioma Yanesha' (amuesha)*. Peru: Ministerio de Educación and SIL.
Dutton, Thomas E. 1993. Possession in Koiari. *Language and Linguistics in Melanesia* 24(1). 39–58.
Dutton, Thomas E. 1996. *Koiari*. Munich: Lincom.
Einaudi, Paula Ferris. 1976. A grammar of Biloxi. New York: Garland.
Elliott, Eric Bryant. 1999. *Dictionary of Rincon Luiseño.* PhD, University of California, San Diego.
Feeling, Durbin. 1975. *Cherokee-English dictionary.* Tahlequah: Cherokee Nation of Oklahoma.
Foley, William A. 2008. The place of Philippine languages in a typology of voice systems. In Peter Austin & Simon Musgrave, eds., *Voice and Grammatical Relations in Austronesian Languages,* 22–44. Stanford: CSLI.
Foley, William A. 2017. Structural and semantic dependencies in word class membership. In Nick Enfield (ed.) *Dependencies in language: On the causal ontology of linguistic systems*, 179–195. Berlin: Language Science Press.
Foley, William A. In preparation. *The epidemiology of language: The evolution of word class categorialization in the Austronesian languages*.
González, Hebe Alicia. 2005. *A grammar of Tapiete (Tupi-Guarani).* PhD, University of Pittsburgh.
Gordon, Lynn. 1986. *Maricopa morphology and syntax*. University of California Press: Berkeley-Los Angeles.
Graczyk, Randolph. 2007 *A Grammar of Crow*. Lincoln: University of Nebraska Press.
Gustafson, Bert. 2014. Guaraní. In Pieter Muysken & Mily Crevels, eds., *Lenguas de Bolivia,* 307–368. La Paz: Plural Editores.
Harrison, S. P. 1988. A plausible history for Micronesian possessive classifiers. *Oceanic Linguistics* 27(1/2). 63–78.
Hill, Jane H. 2014. *A grammar of Cupeño*. Berkeley-Los Angeles: University of California Press.
Himmelmann, Nikolaus P. 2008. Lexical categories and voice in Tagalog. In Peter K. Austin & Simon Musgrave, eds., *Voice and grammatical relations in Austronesian languages,* 247–293. Stanford: CSLI.
Hopi Dictionary Project. 1998. *Hopi Dictionary*. Tucson: University of Arizona Press.
Jacobsen, William H., Jr. 1964. *A Grammar of the Washo Language.* Ph.D., University of California, Berkeley.
Jensen, Cheryl. 1998. Comparative Tupi-Guarani syntax. In Desmond C. Derbyshire & Geoffrey K. Pullum, eds., *Handbook of Amazonian Languages,* 489–616. Berlin: Mouton de Gruyter.
Koehn, Sally Sharp. 1994. The use of generic terms in Apalaí genitive constructions. *Revista latinoamericana de estudios ethnolingüísticos* 8.
Kroeber, A. L. & George William Grace. 1960. *The Sparkman grammar of Luiseño*. Berkeley-Los

Angeles: University of California Press.
Kubodera, Itsuhiko. 1977. *Ainu jojishi shin'yō seiden-no kenkyū* [The study of Ainu heroic epics and songs of gods]. Tokyo: Iwanami Shoten.
Lachler, J. 2006. *A grammar of Laguna Keres.* PhD dissertation, University of New Mexico.
Langdon, Margaret H. 1970. *A grammar of Diegueño: The Mesa Grande dialect*. Berkeley-Los Angeles: University of California Press.
Langacker, Ronald W., ed. *Studies in Uto-Aztecan grammar, vol. 1: An overview of Uto-Aztecan grammar.* Dallas and Arlington: SIL and University of Texas, Arlington, 1977.
Lastra de Suarez, Yolanda. 1984. Chichimeco Jonaz. In Munro S. Edmonson & Patricia A. Andrews, eds., *Supplement to the handbook of Middle American Indians: Linguistics,* Austin: University of Texas Press.
Lichtenberk, Frantisek. 2018. The diachrony of Oceanic possessive classifiers. In William B. McGregor & Søren Wichmann, eds., *The diachrony of classification systems,* 165–200. Amsterdam: Benjamins.
Liljeblad, Sven, Catherine S. Fowler and Glenda Powell. 2012. *The Northern Paiute-Bannock dictionary*. Salt Lake City: University of Utah Press.
Lynch, John, Malcolm D. Ross and Terry Crowley. 2002. *The Oceanic languages*. Richmond, Surrey: Curzon.
Marlett, Stephen A. 1981. *The structure of Seri.* PhD dissertation, University of California, San Diego.
Martin, Jack B. 2011. *A grammar of Creek (Muskogee)*. Lincoln: University of Nebraska Press.
Meira, Sergio. 1999. *A grammar of Tiriyó.* PhD dissertation, Rice University.
Michael, Lev. 2008. *Nanti evidential practice: Language, knowledge, and social practice in an Amazonian society.* PhD dissertation, University of Texas.
Miller, Wick R. 1965. *Acoma grammar and texts*. (UCPL 40.) Berkeley-Los Angeles: University of California Press.
Miller, Amy. 2001. *A grammar of Jamul Tiipay*. Berlin: Mouton de Gruyter.
Mithun, Marianne. 2001. The difference a category makes in the expression of possession and inalienability. In *Dimensions of possession*, ed. Irène Baron, Michael Herslund, and Finn Sørenson, 285–310. Amsterdam-Philadelphia: Benjamins.
Mixco, Mauricio. 1971. *Kiliwa grammar.* PhD dissertation, University of California, Berkeley.
Montgomery-Anderson, Brad. 2015. *Cherokee reference grammar*. Norman: University of Oklahoma Press.
Mora-Marín, David. 2021. Reconstructing possessive morphology in Mayan languages. To appear in *IJAL* 87.
Nakagawa, Hiroshi. 1995. *Ainugo Chitose hōgen jiten* [A dictionary of the Chitose dialect of Ainu].
Tokyo: Sōfūkan.
Nakayama, Toshihide. 2001. *Nuuchahnulth (Nootka) Morphosyntax*. (UCPL 134.) Berkeley-Los Angeles: University of California Press.
Nercesian, Verónica. 2014. *Wichi lhomtes: Estudio de la gramática y la ineracción fonología-morfología-sintaxis-semántica*. München: Lincom Europa.
Obata, Kazuko. 2003. *A grammar of Bilua*. Canberra: Research School of Pacific and Asian Studies, Australian National University.
Odé, Cecilia. 2002. A sketch of Mpur. In Ger P. Reesink (ed.) *Languages of the eastern Bird's Head*. 45–107. Canberra: Pacific Linguistics.
Olawsky, Knut J. 2006. *A grammar of Urarina*. Berlin: Mouton de Gruyter.
Oliverio, Giulia R. M. 1996. A grammar and dictionary of Tutelo. PhD dissertation, University of Kansas.
Onishi, Masayuki. 2012. *A grammar of Motuna*. München: Lincom Europa.
Ozanne-Riviere, Françoise. 1976. *Le Iaai: Langue mélanésienne d'Ouvéa (Nouvelle-Calédonie)*. Paris: SELAF.
Payne, Doris L. & Thomas E. Payne. 2013. *A typological grammar of Panare*. Berlin: De Gruyter

Mouton.
Press, Margaret L. 1980. *Chemehuevi: Grammar and lexicon*. (UCPL 92.) Berkeley-Los Angeles: University of California Press.
Price, David S., Tamara R. Price, Peter J. Silzer, Cheryl Silzer, & Nataniel Merasi. *Ambai-Indonesian dictionary.* n.d. MS, SIL, Papua Branch.
Ravinski, Christine. 2005. *Grammatical possession in Nuu-Chah-Nulth.* M.A. thesis, University of British Columbia.
Reesink, Ger P. 2005. Sulka of East New Britain: A mixture of Oceanic and Papuan traits. *Oceanic Linguistics* 44(1). 145–193.
Reinoso Galindo, Andrés Eduardo. 1999. *Elementos para una gramática de la lengua piapoco*. Bogotá: Ministerio de Cultura.
Rood, David S. 1976. *Wichita grammar*. New York/London: Garland.
Rose, Françoise. 2003. *Morphosyntaxe de l'émérillon: Langue tupi-guarani de Guyane française.* PhD, Université Lumière Lyon 2.
Rosenthal, Jane M. 1981. How Uto-Aztecan is the Nahuatl possessive? In Frances Karttunen, (ed.), *Nahuatl studies,* 182–214.
Sandalo, Filomena. 1995. *A grammar of Kadiweu*. Ph.D. dissertation, University of Pittsburgh.
Sapir, Edward. 1930. *Southern Paiute, a Shoshonean language*. Boston: American Academy of Arts and Sciences.
Satō, Tomomi. 2003. Sakata shiritsu mitsuoka bunko shozō Ezo-ki no Ainugo ni tsuite [Notes on an old Ainu vocabulary in the Sakata City Kōkyū Library]. *Hokkaidō Daigaku Bungaku Kenkyūka Kiyō* 111. 95-118.
Saxton, Dean. 1982. Papago. In Ronald W. Langacker, (ed.), *Uto-Aztecan grammatical sketches, v. 3,* Dallas and Arlington: SIL and University of Texas, Arlington.
Schneider, Joseph. *Grammatik der SulkaSsprache (Neubritannien)*. (Micro-Biblioteca Anthropos, 36.) Posieux: Anthropos Institut, 1962.
Seiler, Hansjakob. 1977. *Cahuilla Grammar*. Banning, CA: Malki Museum Press.
Seiler, Hansjakob. 1995. Possession and classifiers in Cahuilla (Uto-Aztecan). Egon Renner, Michael Dürr & Wolfgang Oleschinski, eds., *Language and Culture in Native North America: Studies in honor of Heinz-Jürgen Pinnow*, 211-225. Munich: Lincom.
Seiler, Walter. 1985. *Imonda, a Papuan language*. Canberra: Research School of Pacific Studies Australian National University.
Seki, Lucy. 2000. Gramática do kamaiurá: Língua tupi-guarani do Alto Xingu. Campinas - São Paulo: Editora da Unicamp & Imprensa Oficial SP.
Shaul, David Leedom. 2012. *Eastern Shoshone working dictionary*. MS.
Silzer, Peter James. 1983. *Ambai: An Austronesian Language of Irian Jaya, Indonesia.* Phd Dissertation, Australian National University.
Steinhauer, Hein. 1977. "Going" and "coming" in the Blagar of Dolap (Pura, Alor, Indonesia). *NUSA: Miscellaneous Studies in Indonesian and Languages of Indonesia* 4. 39–49.
Steinhauer, Hein. 1991. Demonstratives in the Blagar language of Dolap (Pura, Alor, Indonesia). In *Papers in Papuan linguistics*, ed. Tom Dutton, 177–221. Canberra: Pacific Linguistics.
Tavares, Petronila da Silva. 2005. *A grammar of Wayana.* PhD dissertation, Rice University.
Tharp, Doug. 1996. Sulka grammar essentials. In John M. Clifton (ed.) *Two non-Austronesian grammars from the islands*. 77–179. Ukarumpa: SIL.
Vallejos, Rosa. 2016. *A grammar of Kukama-Kukamiria: A language from the Amazon*. Leiden: Brill.
van der Voort, Hein. 2004. *A Grammar of Kwaza*. Berlin: Mouton de Gruyter.
van Gijn, Rik. 2006. A grammar of Yurakaré. PhD dissertation, Radboud University.
Velasquez-Castillo, Maura. 1996. *The Grammar of Possession: Inalienability, incorporation, and possessor ascension in Guaraní*. Amsterdam: Benjamins.
Voegelin, C. F. 1935. *Tübatulabal grammar.* (UCPAAE 34:2.) Berkeley: University of California Press.
Voegelin, Charles F. 1958. Working dictionary of Tübatulabal. *IJAL* 24.221-228.

Watahomigie, Lucille J., Jorigine Bender & Akira Y. Yamamoto. 1982. *Hualapai reference grammar*. Los Angeles: American Indian Studies Center, UCLA.

Willett, Thomas L. 1991. *A reference grammar of Southeastern Tepehuan*. Dallas: SIL and University of Texas at Arlington.

Williams, James. 1932. *Grammar, notes, and vocabulary of the Makuchi Indians of Guiana*. St. Gabriel-Mödlung.

Wilson, Peter J. 1992. *Una description preliminar de la gramatica del achagua (arawak)*. Bogotá: ILV [i.e. SIL].

Anna Bugaeva, Johanna Nichols, and Balthasar Bickel

Appositive possession in Ainu and around the Pacific:

Supplement 2. Statistical analysis *

S2.1. Data

AUTOTYP (Bickel et al. 2017) only contains few entries on non-possessibles, but we collected more data for WALS (Nichols & Bickel 2005). We read in the WALS data via the R package lingtypology (Moroz 2020) so we have convenient access to Glottocodes for data matching. We re-code the entries as binary and remove Hixkaryana because closer inspection shows the data is in fact unclear.

```
wals.df <- wals.feature('58b') %>%
  rename_with(str_to_title) %>%
  filter(!Language %in% "Hixkaryana") %>%
  mutate(AppPoss.Presence = ifelse(`58b` %in% 'None reported', 'absent',
                                                          'present'))
```

We merge this data set with the appendix of the main paper, giving precedence to the newer data. We furthermore add genealogical and geographical information from the AUTOTYP register (v. 0.1.2) and define the Circum-Pacific area as in previous work (Bickel & Nichols 2005). Continent and Area definitions are explained and visualized in the documentation of the AUTOTYP repository (Bickel et al. 2017).

```
appendix.df <- read.xlsx('Supplement1.xlsx', startRow = 2)

poss.df <- rbind(appendix.df[,
                             c('Glottocode','AppPoss.Presence')],
                 wals.df[!wals.df$Glottocode %in% appendix.df$Glottocode,
                         c('Glottocode','AppPoss.Presence')])

poss.g.df <- inner_join(poss.df,
  read.csv('https://raw.githubusercontent.com/autotyp/autotyp-data/v0.1.2/data/Register.csv')) %>%
  # removing multiplication by doculects that share the same Glottocode and AppPoss value:
  distinct(Glottocode, AppPoss.Presence, .keep_all = T) %>%
  mutate(AncientArea = ifelse(Continent %in% c('Australia',
                                               'NG and Oceania',
                                               'W N America',
                                               'S America',
                                               'C America',
                                               'E N America') |
                              Area %in% 'N Coast Asia',
                 "Inside Circum-Pacific", "Outside Circum-Pacific"),
         AN = ifelse(Stock %in% 'Austronesian', 'Austronesian', 'Other')
        )
```

S2.2. Family bias analysis

Only few of the families with appositive possessives have sufficiently resolved phylogenies. In such situations, simple per-family proportions pick up similar signals as explicit evolutionary models in an

*This document was rmarkdown::render'ed from an R script available at https://osf.io/p5xky

Corresponding author: balthasar.bickel@uzh.ch For author affiliations see main article.

MCMC framework (Bickel 2020). A suitable proportion method is the *Family Bias Method* (Zakharko & Bickel 2011; Bickel 2011), which also performs extrapolations for estimating biases in small families and isolates (Bickel 2013) by combining the observed data with empirically trained priors on biases (in any direction) vs. non-biases (chance developments).

```
poss.fam <- familybias(poss.g.df,
                       family.names = c('Stock',
                                        'MajorBranch',
                                        'SubBranch',
                                        'SubSubBranch',
                                        'Language'),
                       r.name = 'AppPoss.Presence', p.names = 'AncientArea',
                       lapplyfunc = mclapply, B = 4000) # number of extrapolations
```

We summarize the results by limiting our attention to families estimated as biased towards or against appositive possessives because the others are uninformative (Bickel 2013). Figure S1 plots the estimates with the uncertainty from the extrapolation runs.

```
poss.fam.e <- do.call(rbind, mclapply(poss.fam$extrapolations, function(e) {
   df <- as.data.frame(xtabs( ~ majority.response + AncientArea,
                       subset = distribution  %in% 'biased', data = e,
                       drop.unused.levels = T))
  df$majority.response <- factor(df$majority.response, levels = c('present', 'absent'))
  return(df)
}))
```

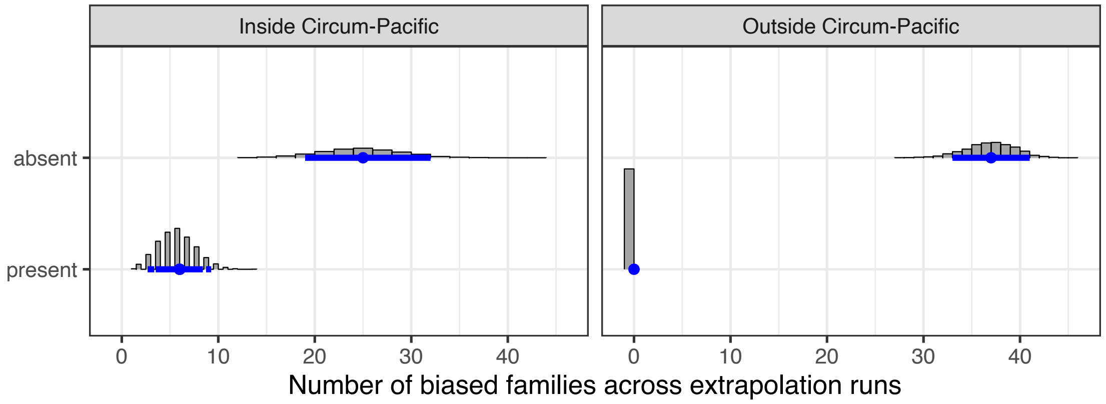

Figure S2.1: Estimated number of families with a bias towards appositive possessives ("present") vs. against appositive possessives ("absent"). Blue dots represent medians and blue horizontal lines the 90% highest density interval.

We capture the main effect of interest in terms of *Odds Ratios (OR)*, which report how many times higher the odds for vs. against appositive possessive biases are inside as opposed to outside the Circum-Pacific region. Our estimates for appositive possessive biases outside the Circum-Pacific are all zero, and so we add a .5 correction for continuity (Agresti 2002).

We also assess the *Reliability Landscape* of the effect (Janssen et al. 2006), comparing the estimated OR with counter-factual scenarios: how many more families might one discover with a bias towards appositive possessives outside the Circum-Pacific so that the effect would disappear? We include these estimates in Figure 2 of the main text, with 90% highest density intervals (HDI). Table S2 gives a numerical overview.

Our Reliability Landscape is conservative since we only explore what goes against our hypothesis. Of course, it is just as likely that we underestimate the appositive possessive biases in the Circum-Pacific, which would increase the effect. A case in point is the South Halmahera and Western New Guinea branch of Oceanic, for which there is actually good evidence that it is biased towards appositive

possessives (see the discussion in the main paper), although we didn't survey the branch at the level of detail required for the present analysis.

```
poss.fam.ORs <- do.call(rbind, mclapply(poss.fam$extrapolations, function(e) {
  e <- subset(e, distribution  %in% 'biased')
  e$majority.response <- factor(e$majority.response,
                                levels = c("absent", "present"))
  tab <- xtabs( ~ majority.response + AncientArea,
              data = e, drop.unused.levels = F) + .5
  # odds of presence:absence inside divided by the same odds outside:
  data.frame(OR = (tab[2,1]/tab[1,1])/(tab[2,2]/tab[1,2]),
             # adding 1...4 to "present outside CP" (cell 2,2):
             ORplus1 = (tab[2,1]/tab[1,1])/((tab[2,2]+1)/tab[1,2]),
             ORplus2 = (tab[2,1]/tab[1,1])/((tab[2,2]+2)/tab[1,2]),
             ORplus3 = (tab[2,1]/tab[1,1])/((tab[2,2]+3)/tab[1,2]),
             ORplus4 = (tab[2,1]/tab[1,1])/((tab[2,2]+4)/tab[1,2])
  ) })) %>%
  pivot_longer(cols = everything(), names_to ="Scenario", values_to = "OR") %>%
  mutate(Scenario = factor(Scenario,
                           levels = unique(Scenario),
                           labels = c('Obs.', '+1', '+2', '+3', '+4')))
```

Table S2.2: OR estimates in the observed data and counterfactual situations (like in Figure 2 of the main text), with 90% highest density intervals

Scenario	Median	90%CI
Obs.	18.67	[8.19, 30.49]
+1	6.22	[2.73, 10.16]
+2	3.73	[1.64, 6.1]
+3	2.67	[1.17, 4.36]
+4	2.07	[0.91, 3.39]

References

Agresti, Alan. 2002. *Categorical Data Analysis*. New York: Wiley-Interscience.

Bickel, Balthasar. 2011. Statistical Modeling of Language Universals. *Linguistic Typology* 15. 401–414.

Bickel, Balthasar. 2013. Distributional Biases in Language Families. In Balthasar Bickel, Lenore A. Grenoble, David A. Peterson & Alan Timberlake (eds.), *Language Typology and Historical Contingency*, 415–444. Amsterdam: Benjamins.

Bickel, Balthasar. 2020. Large and Ancient Linguistic Areas. In Mily Crevels & Pieter Muysken (eds.), *Language Dispersal, Diversification, and Contact: A Global Perspective*. Oxford: Oxford University Press.

Bickel, Balthasar & Johanna Nichols. 2005. Inclusive/Exclusive as Person Vs. Number Categories Worldwide. In Elena Filimonova (ed.), *Clusivity*, 47–70. Amsterdam: Benjamins.

Bickel, Balthasar, Johanna Nichols, Taras Zakharko, Alena Witzlack-Makarevich, Kristine Hildebrandt, Michael Rießler, Lennard Bierkandt, Fernando Zúñiga & John B Lowe. 2017. *The AUTOTYP Typological Databases, Version 0.1.2*. GitHub [https://github.com/autotyp/autotyp-data].

Janssen, Dirk, Balthasar Bickel & Fernando Zúñiga. 2006. Randomization Tests in Language Typology. *Linguistic Typology* 10. 419–440.

Moroz, George. 2020. `lingtypology`: Easy Mapping for Linguistic Typology. R package, https://CRAN.R-project.org/package=lingtypology, v. 1.14.

Nichols, Johanna & Balthasar Bickel. 2005. Possessive Classification and Obligatory Possessive Inflection. In Martin Haspelmath, Matthew S. Dryer, David Gil & Bernard Comrie (eds.), *The World Atlas of Language Structures*, 242–245. Oxford: Oxford University Press.

Zakharko, Taras & Balthasar Bickel. 2011. `familybias`: Family Bias Estimation. R package, https://github.com/IVS-UZH.

Anna Bugaeva*, Johanna Nichols*, and Balthasar Bickel*

Appositive possession in Ainu and around the Pacific:
Supplement 3. Survey language data

https://doi.org/10.1515/lingty-2021-2079
Received August 1, 2019; accepted January 12, 2021; published online June 1, 2021

Abstract: This supplement has two goals: (1) to give information on possessibility classes and possessive marking in all languages we surveyed that have appositive constructions or other non-possessibility; (2) to make available a body of data with consistent analyses, terminology, and glosses. Languages are ordered by continent and then family, clockwise from Ainu. The sections in order are:

S3.1. Ainu
S3.2. North America
S3.3. Mexico-Central America
S3.4. South America
S3.5. Pacific: Austronesian
S3.6. Pacific: Non-Austronesian
S3.7. References

S3.1. Ainu (ainu1240)

Additional examples of old classificatory verbs are the following.

(S1)	*a-**mi***	*kosonte*	*a-e-oriki-kut-kor*	*kane*
	4.A-wear	Japanese.kimono	4.A-APPL-upwards-belt-have	doing.so

‘I put my Japanese kimono on.’ (Satō 2003)

(S2)	*a-**mi***	*hayokpe*	*opitta*	*a-yay-ko-anu*	*wa*
	4.A-wear	armor	all	4.A-REFL-to.APPL-put	and

‘I took off all my armor’ (Nakagawa 1995)

The nouns attested with *mi* ‘wear’ are limited to *kosonte* ‘Japanese short-sleeved kimono’ and *hayokpe* ‘armor’. The referent of the latter is also of Japanese origin, which additionally shows that the essential starting point of non-possessibility is unfamiliarity.

(S3)	*a-**resu***	*kamuy*	*a-**resu***	*pito*	*e-i-nu*
	4.A-raise	god/spirit	4.A-raise	god/spirit	2SG.S-APASS-hear
	kat-u	*ene*	*an*	*hi*	
	shape-POSS	like.this	exist.SG	NMLZ	

‘My spirit, my spirit, listen carefully to what I have to say.’, lit. ‘…your listening shape is like this.’ (Kubodera 1977: 450)

Corresponding authors: bugaeva@rs.tus.ac.jp; johanna@berkeley.edu; balthasar.bickel@uzh.ch
For affiliations see main article.

Here too we are dealing with two old loanwords from Japanese, i.e. *kamuy* 'god/spirit' (< OJ *kamï* 'god, spirit') and *pito* 'god/spirit' (< OJ *pito* 'man, human'), which is consistent with the above scenario. Other nouns attested with *resu* 'raise' are *pewre-p* 'bear-cub', which was ritually raised in a cage in Ainu villages and killed to be sent off to the land of gods, and *ca-pe* (onomatopoeia-thing) 'cat', whose referent is borrowed from the Japanese.

A number of kin terms in Ainu are variable in that they can appear as both obligatorily possessed and non-possessible with *kor* 'have'.

(S4a) ***a****-mat-****ihi***
4.A-wife-PSD
'my wife' (Chiri and Kindaichi 1936(1974): 28)

(S4b) *a-****kor*** *mat*
4.A-have wife
'my wife' (Chiri and Kindaichi 1936(1974): 28)

The semantics of affection seems to be a factor that can determine the choice, but the structural fact is that these words are variable.

Moreover, some kinship terms are also attested in puzzling obligatory possessed and non-possessible combinations, which are regarded as symptoms of language attrition in Chiri and Kindaichi (1936(1974): 29) and Chiri (1942(1973): 533). Yet, such examples are not rare in Classical Ainu literature *yukar* 'heroic epics' and *kamuy yukar* 'divine songs', and on top of this they are found with other classificatory verbs too as in (S6) and (S7), cf. (25) in the main text.

(S5) *a-****kor*** *mac-****ihi***
1SG.A-have wife-PSD
'my wife' (Chiri and Kindaichi 1936(1974): 29)

(S6) *a-****an-te*** *mac-****i*** *itak-an* *ciki*
4.A-exist-CAUS wife-PSD speak-4.S if
pirka-no *e-nu* *kus* *ne* *na*
be.good-ADV 2SG.A-hear because COP SGST
'My wife, if I speak, you should listen well.', lit. 'lit. 'husband (who) I made stay (with myself)' (Bugaeva 2004: 191)

(S7) *an-****ante*** *hoku-****hu*** *e-ikka* *kusu*
4.A-exist.SG-CAUS husband-PSD APPL-steal because
'She stole my husband.', lit. 'husband (who) I made stay (with myself)'
(Kubodera 1977: 244)

Kinship terms are likely to be the first pioneers moving out of the obligatorily possessed class so obligatory possessed while non-possessible combinations can be regarded as an intermediate stage in this process of change in classification in Ainu, which is valuable for understanding the diachrony of how word classes evolve.

S3.2. North America

For the Pacific Northwest (Alaska to northern California), all languages surveyed lack appositive possession.

S3.2.1. Wakashan

Nuuchahnulth (nuuc1236, Southern Wakashan; Vancouver Island, Canada): There is no appositive possession, but apparently all nouns can optionally be treated as non-possessible. Possession can be marked either on the verb, as external possession indexing the possessor (and no possessive marking on the noun), or on the noun with subordinating or relative morphology (Nakayama 2001:128, Ravinski 2005). The PSD suffix makes an alienability distinction (Ravinski 2005) and 'wife' is alienable.

(S8) *7aap-h.ii-7a:k-s* *łuucma*
kind-DUR-PSD:AL-1sg wife
'my wife is kind' (Nakayama 128)
(lit. 'my-kind-one, wife') 7 = glottal stop; h. = h with underdot

(S9) *7aap-h.ii-7iš* *łuucma-7a:k-qs*
kind-DUR-IND.3 wife-PSD:AL-SUB.1sg
(lit. 'she-is-kind, my-wife')
id. (ibid.)

S3.2.2. Uto-Aztecan

The Uto-Aztecan family extends well into both North America and Mexico, but for convenience all of it is covered in this section. The family is divided into two halves, for which we use the abbreviations NUA = Northern Uto-Aztecan and SUA = Southern Uto-Aztecan.

Most Uto-Aztecan languages have possessive affixes or clitics indexing the person-number of the possessor, e.g. Cupeño (cupe1243, NUA: Cupan):

(S10) *nu-yu*
1sg-hair
'my hair' (Hill 2014:167)

Most make an alienable/inalienable distinction. Commonly, inalienables are obligatorily possessed and alienables optionally possessed. Inalienables usually include kin terms, body parts, and a few other close possessions.

In addition, most of the languages have a set of NPNs (Uto-Aztecanist term: *absolutive* suffixes) found on nouns that do not have possessive morphology or other suffixal morphology or compounded elements. Some of the languages also have PSD suffixes that cooccur with possessive indexation. E.g. Pipil (pipi1250, SUA: Aztecan):

(S11) *siwa:-t* *nu-siwa:-w* *-siwa:-*
woman-NPN 1sg-woman-PSD
'woman' 'my wife' 'wife' (stem) (Campbell 1985:38, 43)

Cupeño:
(S12) *waka-l* *-waq-ʔa* *-waq-*
spear-NPN spear-PSD
'spear, a spear' 'spear' (lexeme) 'spear' (stem)
(Hill 2014:172)

See also Sapir (1930:120–122) for Southern Paiute (utes1238) examples, and Langacker (1977:88).

Several of the languages have a set of non-possessible nouns, always including animal names and sometimes plants and natural phenomena. When the referent of an alienable is owned an appositive construction with a classifier noun is used. E.g. Cupeño (Hill 2014:129, 176–177):

(S13) *awal* *ne-ʔash*
dog 1sg-CL:pet
'my dog'

(S14) *wi-lʸ* *ne-mixan*
lard-NPN 1sg-CL:possession
'my lard' (cooking lard) [constructed from Hill's table (176)]

Languages at the far peripheries of the family's range have minimal or no appositive possession. Pipil (Aztecan subbranch; Campbell 1985), spoken in Nicaragua at the far southern limit of the Aztecan branch, has none (nor do other Aztec varieties to our knowledge). In western central Mexico, Cora (elna1235, SUA: Corachol subbranch, just north of Aztecan; Casad 1984) appears not to have appositives or classifiers, but in predicative possession the word 'animal' (sg. *kyi*, pl. *yá'amwa*) is inflected for possession and replaces the verb 'be' used for other possession:

(S15) *ńéeci* *pú* *ńa-kyi*
mine 3sg 1sg-animal
'It's my animal' (236)

(S16) *nye-síiku'u* *pú=pýryky*
1sg-shirt 3sg=be
[it's my shirt] (234)

This may be either a fossil or a possible nascent appositive construction.

The northernmost languages of the Numic branch (the northernmost Uto-Aztecan branch, spoken in and near the Great Basin) also lack appositive constructions in its northernmost languages. All the Numic languages have a noun *puŋku (the Proto-Numic form) or similar, which functions as an independent noun meaning 'pet, domestic animal' and is also the possessive classifier for animals. In Chemehuevi (utes1238) and Southern Paiute of the Southern Numic subbranch, the word is an appositive classifier, taking a cliticized pronominal possessor and compounding with the possessed noun:

(S17) Chemehuevi: *nyyni* *tuku-pungku-n*
1sg.GEN mountain.lion/cat-pet-1sg
'my mountain lion/cat' (Press 1980:60)

(S18) Southern Paiute: *qava:+vungqu-ni*
horse+pet-1sg
'my horse' (Sapir 1930:121)

Languages to the north of these use exclusively dependent-marked possession, or pronominal argument marking in which pronoun possessors cliticize to possessed nouns while nouns take the genitive case. In these languages, the *puŋku reflex and the putative classifier form a compound, e.g. Tümpisa Shoshone (Dayley 1989:189):

(S19) *nia-ng* *[wasüppi* *pungku]*
1sg-GEN mountain sheep pet
'It's my mountain sheep'
(structure: my [mountain.sheep pet], i.e. [my [pet mountain.sheep]])

(S20) *nia-n* *[nütsütü* *pungku]*
1sg.GEN airplane pet
'It's my airplane'[1]

Similarly in Eastern Shoshone (shos1248, Shaul 2012):

(S21) *goso* *vungku*
pig pet
'pig, domesticated pig'

(S22) *wovim* *bungku*
log pet
'wagon' (lit. 'wood[en vehicle')

Such compounds are listed in dictionaries: Eastern Shoshone *goso vungku* (pig pet) 'pig, domesticated pig' (Shaul 2012); Northern Paiute (nort2954) *sadyʔy+puku* (dog+*puku*) 'pet dog' (Liljeblad et al. 2012). In the entries they are cited without possessors, which would not be possible if they were possessive appositives (because then they would be inalienable nouns).

In the Eastern Shoshone examples above, the initial *p- of the classifier undergoes lenition, showing that it forms a close phrase or compound with the possessed noun.[2] In Northern Paiute, the word means 'horse' or 'dog as an independent noun and appears as second element in compounds referring to domestic animals: (*sadyʔy+puku* [dog+*puku*] 'pet dog', Liljeblad et al. 2012 s.v.).

In Tümpisa Shoshone (pana1305) predicative possession, *punku* is incorporated into the verb 'have':

(S23) *Satü* *yühmü* *pungku+pa'e*
DEM porcupine pet+have
'He has a pet porcupine' (Dayley 1989 s.v. *pungku*)

as are other head nouns:

(S24) *Nü* *petü+ppa'i*
1sg daughter+have
'I have a daughter' (Dayley 1989 s.v. *-pa'in*)

The word can also be used as an independent noun meaning 'pet, domestic animal'. There is also a compound *isa+punku* 'dog' (coyote+pet).

---

[1] In Numic languages the word means not only 'dog; horse' but (in a semantic extension from ''horse') 'vehicle, means of transport'.
[2] Dayley's Tümpisa Shoshone practical orthography is at a more abstract level and does not indicate whether (S19-S20) are to be read as non-compound phrases, with [p], or as second elements of compounds, with [β]. Shaul's spelling is unambiguous.

In these Numic languages, then, the possessed noun and the *puŋku reflex form a compound or close phrase, and the *puŋku reflex is an ordinary alienable noun. Since these languages use a genitive case in some or all possessive phrases, and the cognate to the possessive appositive of sister languages with head-marked possession is appositive but not a possessive appositive, they are consistent with the generalization that appositive possessives occur only in head-marked phrases.

Farther south in the Numic branch, Chemehuevi, the southernmost Southern Numic language, has a two-classifier appositive system, using the inalienable noun -*pungku* in appositive constructions and incorporation (Press 1980:60):

(S25) *nyyni* *tuku-pungku-n*
1sg.GEN mountain.lion/cat-pet-1sg
'my mountain lion/cat'

(S26) *nyy-k* *waha-ku-my* *waʔarovi-my* *pungku-wy-ga-nt*
1sg-K two-OBL-ANIM.OBL horse-PL.OBL pet-PL-have-PCP
'I have two horses' (K = 3rd person inanimate)

and -*ygapy* 'plant (not wild)' for plants (115; no examples given).

Southern Paiute, a close sister of Chemehuevi, has only -*pungqu* 'domesticated animal, owned animal; horse, dog', which can be used by itself or as an appositive classifier (Sapir 1930:121):

(S27) *qava:+vungqu-ni*
horse-pet-1sg
'my horse'

(S28) *pungqu-ni*
pet-1sg
'my horse'

Qava 'horse' is alienable. We assume that *pungqu-* is probably inalienable when used by itself.

In all of the Numic languages the **puŋku* reflex forms a compound or close phrase with the possessed noun, unlike most appositive possessive words. It can be analyzed as a construct morpheme, one that registers the presence of a possessor (or the fact of possession) on the head. Unlike most construct morphemes, it also functions as an independent word. Given that the possessive phrases using it are spelled and pronounced as compounds, it is not an inflectional morpheme but derivational.

There is no discussion of whether words for domesticated animals and plants are non-possessible in these languages. There are examples showing that they are possessible, without the appositive, in other Numic languages, e.g. Tümpisa Shoshone:

(S29) *Satümmü* *tammi* *putisiha* *innüntükkanna*
those INCL-GEN burro-ACC steal
'They stole our burros' (Dayley 1989:190)

Tübatulabal (tuba1278), an NUA isolate branch, has both possessive affixes and a genitive case. It has obligatorily possessed inalienables (kin terms, some body parts, and a few special terms) and non-possessibles (proper names and some plant and animal names), but no information is given about possession of non-possessibles (Voegelin 1935:140). There is a cognate to *puŋku in

the verb *punggu?*- 'domesticate' and the (evidently suffixed) noun *pukubiš-t* 'dog' (Voegelin 1958 s.vv.) but no reported appositive classifier.

Hopi, another isolate NUA branch, has what appears to be a fossil appositive construction using the cognate to the Numic and Tübatulabal form. Most possessed nouns, including names of commonly possessed animals, take the regular prefixal possessive markers. Names of animals not usually possessed compound the noun with *pooko,* cognate to *puŋku:

(S30) *mosa* 'cat' *i-mosa* 'my cat'
kwaayo 'hawk' *i-kway-vooko* 'my (captive) hawk'
(Hopi Dictionary Project 1998: 873–874)

In the other Northern Uto-Aztecan languages we surveyed, Cupeño, Cahuilla, and Luiseño (luis1253, Cupan branch) use head-marked possession and have possessive appositives:

(S31) Cupeño *awal ne-ʔash*
dog 1sg-CL:pet
'my dog' (Hill 2014:129)

(S32) Cahuilla *né-ʔaš ʔáwal*
1sg-own dog
'my dog' (Seiler 1995:219)

(S33) Luiseño *no-ʔa:š awá:l*
1sg-CL:pet dog
'my dog' (Kroeber and Grace 1960:82)[3]

Languages of the SUA Tarahumara-Guarijio branch (northern Mexico) have appositives of different types. The Choguitá Rarámuri (lowl1265) variety of Central Tarahumara (Caballero in prep.) has neither genitive case nor person marking on the possessed noun, but uses PSD suffixes to register a possessor. The suffix *-la* (henceforth we cite examples in the more surface of Caballero's two levels) is found on both inalienables (where it is obligatory) and alienables. Some nouns can add a second suffix *-wa* before *-la*.

(S34) a *ne* *wasá-**la***
1sg.NOM cultivation.land-PSD

b *ne* *wasá-**wa-la***
1sg.NOM field-PSD-PSD
both 'my cultivation land'

The second suffix is standard for some nouns, impossible for some, and optional for many as in these examples. Where it is optional, for the most part there is no detectable semantic difference.

Some inalienables add a third suffix, the locative *-či* (added without regard to the syntactic function, i.e. not in a locative context):

[3] Kroeber and Grace do not write the initial glottal stop; Elliott (1999) does write it in similar examples. We have therefore added the glottal stop where it is lexeme-initial but word-internal, as Hill writes it (Seiler writes it even word-initially).

(S35) *nehé* *kawí-**wa-la-či***
1sg.NOM hill-PSD-PSD-LOC
'my hill'

Another possessive construction uses an appositive clause with the possessive noun *nía*, which takes the PSD suffix:

(S36) *kúmi* *bu'í* *ne* *nía-**la*** *wičá*
where lie.sg 1sg.NOM POSS-PSD needle
Where is my needle?

(S37) *na* *ko* *ne* *nía-**la*** *líbro* *ko*
PROX EMPH 1sg POSS-PSD book EMPH
'This here is my book'

Exs. (S38a–b) show *-la* present on an inalienable (where it is obligatory) and absent from the same word in its alienable sense (which when possessed requires *nía*):

(S38a) *nehé* *sa'pá-**la***
1sg meat-PSD
'my flesh (of my body)'

(S38b) *nehé* *nía-**la*** *sa'pá*
1sg POSS-PSD meat
'It is mine the meat (to eat)' [We assume also NP 'my meat' (i.e. to eat)]

The possessive noun can also funtion as a verb in possessive predication, glossable as 'have'. This is the only appositive noun in Choguita Rarámuri.

Rarámuri as reported by Rosenthal (1981) has two classificatory possessive verbs, one for ownership of animals: *buku-,* cognate to the Numic 'pet' classifier, in Rarámuri a verb meaning 'own (of animal)' (Rosenthal 1981:196, without source but evidently Brambila 1953).

(S39) *nihe* *buku-ra* *gawe*
1sg pet-PSD horse
'my horse'

The remaining surveyed UA languages have classifier systems with non-possessibles (or apparent non-possessibles). In the SUA Tepiman branch (which extends from central Mexico to southern Arizona), Southeastern Tepehuan (sout2976, Willett 1991) uses head-marked possession with person-number markers that are proclitic to the noun (suffix for 3sg) or enclitic to a preposed article. It is obligatory with inalienables (kin terms, body parts, meronyms).

(S40) *gu - ñ* *moʔ*
ART-1sg head
'my head' (82)

There is a PSD suffix *-ga- / -ʔ-* used on words for domestic animals, affinal kin terms, plant and animal parts, and some basic possessed items such as 'house', when they are possessed (Willett 1991:211)

(S41)	*gu-m*	*junu-ʔ*
	ART-2sg	corn-PSD
	'your corn'	

(S42)	*gu-m*	*<ca>cvai-ʔ*
	ART-2sg	<RED>horse-PSD
	'...your horses'	[extracted from clause]

(S43)	*gu*	*joñ-ga-ʔn*	*gu*	*Macario*
	ART	wife-PSD-3sg	ART	M.
	'Macario's wife'		[extracted]	

(S44)	*upsu-ga-ʔn*	*kyʔn*	*ty-kykyʔ*	*gu*	*nacsyr*
	stinger-PSD-3sg	with	EXTENT-bite[4]	ART	scorpion
	'The scorpion stings with its stinger'				

(S45)	*Jum-xychuiʔ*	*gu*	*cúpar-ga-ʔn*	*gu-ñ*	*jóco-ʔ*
	RFL-break	ART	top-PSD-3sg	ART-1sg	flashlight
	'The top to my flashlight is broken.'				

As can be seen by scanning the above examples, the nouns that take *-ga / -ʔ* include both words that are cross-linguistically often inalienable (kin terms, body and plant parts, other parts) and frequent non-possessibles (domestic animals, food plant parts). With the typical non-possessibles it has PSD function, but with the inalienables it is pleonastic and divides semantic inalienables into two sets: obligatorily possessed kin terms and human body parts, and affinal terms and non-human parts. The suffix then marks, approximately, things that can be acquired (Willett 1991:211).

Other Tepiman languages have appositive possessive nouns which classify nouns as animate vs. inanimate, and which take suffixes that add further classification. Seiler (1985) reports these two: Tohono O'odham (toho1245, formerly Papago; Saxton 1982:182) has animate *šoi-* and inanimate *ïñ-*, with suffixes *-ka* (possessed plant parts), *-g* (loanwords), *-ga* (others). Northern Tepehuan (nort2959, Bascom 1982:213) has animate *šoi-ga* and inanimate *tʸulʸdʸa-ga*. These then add possessive suffixes. The suffixes *-ga* are cognate to the *-ga* allomorph of the Southeastern Tepehuan PSD suffix (above).

The Cupan branch of NUA provides evidence for evolution of classifier systems, specifically for rapid growth of a small system into a large and open one. Luiseño (southwestern California) has an appositive classifier *-ʔash* for names of animals, which are non-possessible (Kroeber and Grace 1960: 82-83):[5]

(S46)	*awá:l*	'dog'	*no-a:š awá:l*	'my dog'
	no:ta	'gopher'	*no-a:š no:ta*	'my (pet) gopher'

The word also functions as an independent noun *ʔash-la* (with NPN suffix) 'pet, domesticated animal, horse, cow; shaman's familiar', which can also be possessed: *po-ʔásh* 'his horse', 'her cow', etc. (Elliott 1999:135). It also forms a suffixed denominal verb *ʔásh-lu* 'breed (livestock), increase (one's herd)'.

Closely related Cupeño (Arizona; Hill 2014) has a cognate classifier for animals and another for inanimate possessions (examples repeated from above):

---

[4] EXTENT is a preverb, apparently directional or spatial.

[5] Kroeber and Grace do not write the initial glottal stop; Elliott (1999) (cited just below) does.

(S47) *awal* *ne-ʔash*
dog 1sg-CL:pet
'my dog' (129)

(S48) *wi-l*^{y} *ne-mixan*
lard-NPN 1sg-CL:possession
'my lard' (cooking lard) (176, 177)

Cahuilla, a neighbor and closest sister of Cupeño, has cognate nouns in the same two functions, as well as additional semantically based classifiers (Seiler 1995; 1977:298ff.). All but one can be inflected and used as either nouns or verbs, and most are basic verbs as shown by the fact that in their appositive use they take the nominalizer *-ʔa.* (For this suffix see Seiler 1977:298 and Hill 2014:316-319. We use Hill's term *nominalizer.*) Those with the nominalizer are surface nouns in apposition, but basic verbs. There are two animate classifiers, generic *-ʔaš* for domestic animals and pets, and *-kil´iw* 'totem' for the two moiety animals. The first of these appears to be a flexible noun-verb, as it can function as appositive without a nominalizing suffix; the second was nearly obsolete at the time of Seiler's fieldwork and only known as a noun.

(S49a) *né-ʔaš* *ʔáwal*
1sg-own dog
'my dog' (Seiler 1995:219)

(S49b) *pe-n-áš-qal*
3sg.O-1sg-own-DUR
'I own it (as a pet)' (1995:219, 1977:305)

There is a generic inanimate classifier for possessions of all kinds based on a verb root that Seiler glosses 'do' (with INDEF prefix it gives the meaning 'do (in some way)'):

(S50) *qáwiš* *ne-m-éxan-ʔa*
rock 1sg-INDEF-do-NZ
'my rock' (1977:300)

(The Cahuilla word takes nominalizer *-ʔa* when used appositively; the Cupeño examples lack it. For these and other inanimate classifiers Seiler includes the *-ʔa* in the citation form.)

For edible plants and/or their fruit there are classifiers based on traditional relations of ownership and usufruct. All take the nominalizer *-ʔa*.

-kiʔiw-ʔa Group of plants (or the location where they grow), and their fruit (mesquite, oak/acorn, pinyon, chia), from the verb root 'wait'.

(S51a) *ne-kiʔiw-ʔa* *méñikiš*
1sg-wait-NZ mesquite.beans
'my mesquite beans' (1995:221; see also 1977:301)

(S51b) *pe-n-kíʔiw-qal*
3sg-1sg-wait-DUR
'I am waiting for it'

Such places were assigned to clan lineages; "[t]he members of the lineage had a legal claim and were allowed to harvest these places when the appropriate time came" (301).

-ʔay-ʔa Fresh fruit or blossoms picked from the plant: fresh mesquite beans (in the pod), squash blossoms, corn.

(S52a) *ne-ʔáy-ʔa* *méñikiš*
1sg-pick-NZ mesquite.beans
'my mesquite beans (fresh, on the tree)' (221)

(S52b) *pe-n-ʔáy-qal*
3sg-1sg-pick-DUR
'I am picking it'

-či-ʔa Edible items after they have fallen off the plant and are picked up from the ground.

(S53a) *ne-čí-ʔa* *méñikiš*
1sg-gather-NZ mesquite.beans
'my mesquite beans (picked up from the ground)' (221)

(S53b) *pe-n-číči-qal*
3sg-1sg-gather-DUR
'I am picking it up from the ground'

-wés-ʔa Crops planted in a row.

(S54a) *ne-wés-ʔa*
1sg-plant-NZ
'my planting' (1977:303) (no adnominal example given)

(S54b) *pe-n-wés-qal*
3sg-1sg-plant-DUR
'I am planting it' (ibid.)

These classifiers are primary verbs and take the nominalizer to function adnominally. Their meanings as verbs are human actions: 'own a claim (and harvest when ripe)', 'pick, harvest', 'gather', 'plant'. In their adnominal uses they also appear to carry nominal-like quantitative or distributional implications: patch of land where edible plants grow; scattered over the ground; ripe for picking, or fresh; in a row. We assume these are aspects of what is lexicalized in the nominalized form and not inherent in the verb meaning.

There are other classifiers for other food products, based on how they are prepared: *-sex-ʔa* 'cooked', *-waʔ* 'roasted', *-čáxni-ʔa* 'melted', *-téneq-ʔa* 'barbecued':

(S55a) *né-waʔ* *túkut*
1sg-roasted wildcat
'my roasted bobcat meat' (304)

(S55b) *pe-n-wáwa-qal*
3sg-1sg-roast:RED-DUR
'I am roasting it' (304)

(S56a) *ne-čáxni-ʔa* *wíl´*
1sg-melt-NZ lard (305)
'my melted lard'

(S56b) *pe-n-čáxni-qal*
3sg-1sg-melt-DUR
'I am melting it' (305)

-*waʔ* 'roast' does not take the nominalizer and must be a basic noun or flexible noun-verb; the others take it and are basic verbs. All have verbal meanings based on the type of preparation. They appear not to have the quantitative or distributional implications of the edible plant classifiers.

The Cahuilla system is the only large system we have found in Uto-Aztecan. The Cahuilla classifiers use morphology and syntax that are easily available for one type of relativization, which is morphologically minimal and apparently appositive rather than subordinating, as in this example from Cupeño:

(S57) *pe-pulinma pe-ʔayew-ʔa*
3sg-man's.child 3sg-like-NZ[6]
'his son whom he loved' (Hill 2014:317)

Such constructions could easily have been coined to create possessed NPs for owned non-possessible head nouns. Thus we can reconstruct a pre-Cahuilla system in which animal nouns and some inanimates were non-possessible, or perhaps all alienables were non-possessible. Owned non-possessibles could form the appositive construction as needed, and these have been lexicalized for the salient owned or apportioned categories of animals and food items. An open question is whether the foodstuff nouns were strictly grammatically non-possessible: perhaps the possession marked by possessive affixes is semantically a more intimate or inherent relationship that does not apply outside of kin terms, body parts, or certain other closely connected nouns (e.g. a basket which the owner has made, which is inflected as inalienable in Cahuilla); or perhaps notions like 'have', ownership, and possession in Cahuilla and pre-Cahuilla necessarily involve relations of ownership or actions of claiming and are therefore naturally conveyed by verbs. (See Seiler 1977:298–307 and 1995 for a close analysis of the semantics of possession in Cahuilla.)

All three Cupan languages have the appositive classifier *ʔaash 'pet, owned animal', which Rosenthal 1981:198 describes as similar to a 'pet' classifier of the neighboring Yuman language family. She does not give the Yuman form and we have not found it in our survey (§3.3 below); we have found only Tiipay (kumi1248) -*xaṭ*, Hualapai (hava1248) -*hadh.*

The range of Uto-Aztecan appositive possessive systems can be accounted for with two principles: (1) independent elaboration in Cahuilla, using verbs in a relative construction that was generally available and could easily have become standard for certain semantic kinds of possession; and (2) tapering off to both north and south of smaller systems with non-possessible animal names and some inanimates attested in the centrally located languages.[7] In the elaborate Cahuilla system and the simpler Cupeño type from which it evolved, the classificatory appositives are verbs in origin. To judge from the Tübatulabal cognate, the Numic 'pet' classifier may have been a verb in origin.

---

[6] Hill interlinearizes the suffix as PSD, but as this is a type of nominalization we interlinearize it functionally in this example as NZ. It is a single morpheme with those two functions.
[7] Unless the allomorph -*ʔ* of the Southeastern Tepehuan PSD suffix -*ga- / -ʔ-* is cognate to the Cupan nominalizer and is a remnant of a once larger or more open system.

S3.2.3. Washo

Washo (wash1253, isolate, northeastern California and western Nevada; Jacobsen 1964:391–392, 408, 468)has person-number possessive prefixes. Alienables may take prefixes; inalienables, mostly kin terms and body parts, are obligatorily possessed. (Inalienables fall into three morphologically defined subclasses: see Jacobsen's Appendix 3.) Animal and plant names cannot take possessive prefixes but use appositive classifiers: *gúšuʔ* 'pet' for animals and another word for plants (Washo term not given). Other non-possessibles can add the attributive-instrumental prefix when preceded by a noun possessor. We analyze this prefix as PSD. It registers a possessor but does not index it.

(S58)	*t'á:gɨm*	'pine nuts'	vs.	*dabóʔo*	*ʔit-t'á:gɨm*
				white.man	PSD-pinenuts
				'white man's nuts' (i.e. purchased nuts)	

S3.2.4 Yuman[8]

The Yuman family, spread across southern California and Arizona and Baja California, is probably somewhat over 2000 years old. We have surveyed five of its 10 daughters, including representatives of all major branches. The languages typically have a system of three possessive classes. Inalienables directly take possessive prefixes and are obligatorily possessed; they include kin terms, body parts and plant parts, and a few others. (Kin terms and body parts have slightly different allomorphs of their possessive prefixes in some of the languages.) Alienables are optionally possessed, and when possessed some or all of them take a PSD prefix following the person prefix. A possessor noun or pronoun precedes the possessed noun. These two classes have the same structure across the family; the PSD prefix has two allomorphs, contrasting in some languages and leveled in favor of one or the other in most.

Certain other nouns are apparently non-possessible (though never so described in grammars) and take postposed appositive possessive nouns that have some classification, most often 'pet' and some kinds of inanimates, but occasionally others. These are less consistent across the family.

Examples of the three classes from Hualapai (Pai branch; Watahomigie et al. 1982):[9]

Inalienable:

(S59)	*nya*	*ʔ-jídha*	*ma*	*m-jídha*
	I	1-mother	you	2-mother
	'my mother'		'your mother' (173)	

Alienable: In Hualapai the PSD form is used chiefly for nouns referring to real property (land, houses, etc.) (187). Most nouns can use the general appositive construction (below).

(S60)	*nya*	*nyi-ʔ-wa:-v-ch*	*hánkyu*
	1sg	PSD-1-house-DEM-SUBJECT	good
	This house of mine is good. (188)		

The PSD prefix is also used on the animal classifier (just below).

---

8 In Yuman examples we have replaced "ɖ" (with strikethrough) with *dh.* We linearize superscript "y" and "w".

9 This grammar is notable for including sizable lists of nouns in each class and mentioning nouns that can take more than one kind of possessive marking (with discussion of the semantic difference).

Appositive: Names for some domesticated animals and some wild animals in captivity use the animal classifier *-hadh*:

(S61) *waksí ma m-nyi-hádha*
cow you 2-PSD-pet
'your cow' (extracted from clause example) (179)

Other nouns use the general classifier *-wi:-*.[10]

(S62) *madh ma m-wi:-ny-ch hánkyu*
land 2sg 2-POSS-DEM-SUBJECT good
The land that belongs to you is good. (189)

Examples from Jamul Tiipay (Delta-California branch; Miller 2001):[11]

Inalienables:

(S63a) *Ø-shally* (1-hand) 'my hand, arm'
me-shally (2-hand) 'you hand, arm'
Ø-shally (3-hand) 'his/her hand, arm' (146)

(S63b) *Ø-ntaly* (1-mother) 'my mother'
me-ntaly (2-mother) 'your mother'
kwe-ntaly (3-mother) 'his/her mother' (147)

Note the allomorphy of third person prefixes, with Ø- for body parts and *kwe-* for kin terms.

Alienables:

(S64) *Ø-nye-wa* (1-PSD-house) 'my house'
me-nye-wa (2-PSD-house) 'your house'
Ø-nye-wa (3-PSD-house) 'his/her house' (147)

Appositive possessives are not explicitly discussed, but Langdon (1970:170) has this one among her examples of other appositional NPs:[12]

(S65) *ʔa:ṣa: ʔ-ny-xaṭ*
bird 1-PSD-pet
'my pet bird'

For Maricopa (mari1440, River branch) Gordon (1986:33) describes two appositive classifiers: *ny-hat* (PSD-dog) for animals and *ny-wish* for others:

---

[10] The prefixed alienable construction is an alternative. It implies a closer or more immediate, ongoing, possessive relation compared to the appositive one (Watahomigie et al. 188–189).
[11] We have added "Ø" and interlinearized it where there is no overt prefix.
[12] Retranscribed without predictable schwas, to match Miller's transcription.

(S66) *qwaqt* *'-ny-hat*
horse 1-PSD-dog
'my horse'

(S67) *m-ntay* *posh* *ny-hat*
2-mother cat PSD-dog
'your mother's cat'

(S68) *kwar'o* *m-nywish*
knife 2-PSD.POSS
'your knife'

In Cocopa (coco1261, Delta-California branch; Crawford 1966) inalienables are much as in Hualapai and Jamul Tiipay, except that kin terms are denominal verbs, renominalized with the nominalizer *kw-:*

(S69) *ny-šmá:l* (3-ear) 'his/her ear' (98)
kw-ny-a-cáy (NZ-PSD-3-mother)[13] 'his/her mother' (99)

Only a few alienables use the PSD prefix *ny*-:

(S70) *máṭ* 'land, ground' *m-ny-máṭ* 'your land, country'
2-PSD-land
wá 'house' *Ø-ny-wá* 'my house'
kmí 'bag, sack' *Ø-ny-kamí* 'his pocket' (139)

It has an allomorph *i:*- that appears on a few body part nouns:

(S71) *Ø-i:-mí:* 'my foot, leg'
m-i:-yú 'your eye, face' (139)

Most alienable nouns use an appositive construction with a form of the verb *aʔi* 'do'.[14]

(S72) *nyqwál uʔás* 'her front dress' (front:dress 3sg-DO)
nyqwál maʔís 'your front dress' (front:dress 3sg-DO (97)

(S73) *ku:k* *Ø-aʔís* 'my coke', lit. 'I have a coke, the coke I have'
1-do/have (79)

The appositive construction uses a PSD prefix for animate possessum:

(S74) *kwramáṣ* *p-ny-aʔís* 'I have children'
?-PSD-do/have (79)

---

13 Note the homophony of *ny*-, which is PSD for alienables and kin terms but third person for most inalienables. -*a*- of 'his mother' is not originally a member of the person paradigm but an epenthetic or similar vowel, arguably reanalyzed as a person marker (Crawford, 98).
14 Crawford describes the -*s* as a distributive object plural marker, describes the entire construction as apparently nominalized, and translates some examples as finite. We take it to be an internally headed nominalization.

We found no mention of a separate animal classifier.

Kiliwa (kili1268, isolate branch, more distant from the rest; Mixco (1971:106–108) has the same treatment of inalienables, except that some take a PSD prefix *-i-*:[15]

(S75a) *ʔ - ʔíy*
1 head 'my head'

(S75b) *m - yíw*
2 eye 'your eye'

(S75c) *ʔ - i - wáʔ*
1sg-PSD-house 'my house'

(S75d) *m - i - phíʔ*
2 - PSD-nose 'your nose'

Apparently all alienables take one of three shape-based appositive classifiers: *páʔ* 'non-long', *háʔ* 'long', and *č-ʔî* 'perpendicular'. These are shape-classified verbs all meaning 'put, place'.

(S76a) *ximíʔ ʔ - páʔ*
bag 1sg-place:non-long
'my bag' (108)

(S76b) *ʔ that ʔ - háʔ*
1 dog 1-place:long
'my dog' (108)

(S76c) *naitháʔ m - č - ʔî:*
horse 2 - (place:perpendicular)
'your horse' (109)

There is no mention of an animal classifier, and the examples of 'dog' and 'horse' just above indicate that domestic animals take the regular shape-based classifiers.

To summarize, most Yuman languages treat inalienables alike: kin terms, body parts, and a few others take direct possessive affixation; only Kiliwa uses the PSD prefix with some of these. Alienables are more variable. Alienables with PSD are a default class in Jamul Tiipay but a minor class elsewhere, coexisting with appositive classifiers, and absent entirely from Kiliwa. Appositive classifiers are even more variable. The Kiliwa system is entirely different from those of the other languages (consistent with its geographical and phylogenetic separation from the rest of the family). In General Yuman (the non-Kiliwa branch of the family) the appositive classifier *haṭ* for animals and the PSD prefix *ny-* are found in all three subbranches and can be reconstructed to the General Yuman protolanguage, and the general appositive *wi:-* is found in two branches and can possibly be reconstructed for General Yuman. The only structural patterns that are pan-Yuman are the direct affixation with inalienables, which is common worldwide, and the appositives in general (though not specific ones). The appositives are described as verbs or deverbal wherever the point is mentioned (Watahomigie et al. 173, 180; Crawford 1966:97; 168, Mixco 1971:106–108). It is difficult to say whether, over time, it is PSD alienables that have been expanding at the expense of appositive ones or vice versa.

---

[15] We replace Mixco's word-internal plus signs with hyphens.

S3.2.5. Keresan

Laguna (east1472), of the small Keresan family (or more properly dialect chain) of the Pueblos, has four possessive classes (Lachler 2006:54–65): non-possessibles (natural kinds such as plant and animal species, and natural phenomena such as 'snow', 'sun'); optionally possessed nouns (including artifacts, institutions, etc. and also what Lachler terms *dealienables,* alienables derived from inalienables); body part nouns (obligatorily possessed), and kin terms (obligatorily possessed, but with different possessive prefixes from those of body parts). Non-possessibles, when owned (as with domesticated animals), take an appositive construction with the nominalized verb stem -*adyaa-she* (have.as.pet-NZ) with a possessive prefix:

(S77) *kawaayu* *k'-adyaa-she*
horse 3sg-have.as.pet-NZ
'his/her horse' (62)

Wild animal names can occur in this construction if they are pets, e.g. in a myth text:

(S78) *k'-adyaa-she* *muuk'aitra*
3sg-have.as-pet-NZ mountain lion
'his (pet) mountain lion' (63; Boas 1927:07.062)

The same construction, with the cognate possessive noun and also with animal names, occurs in closely related Acoma (west2632):

(S79) *kawâayu* *ś-adyá*
horse 1sg.pet
'my horse' (Miller 1965:147)

and Santa Ana (east1472; Davis 1964:129, unsegmented example with 'his eagle').

S3.2.6. Caddoan

In Wichita (wich1260, northern Texas to Kansas; Rood 1976:143–150), apparently no nouns can take person indexation (or any other inflection; nouns are a non-inflecting part of speech). The only possessive construction is relativization. (The exact form of relativization – argument indexation or incorporation – depends on whether the relative noun is A, S, O, or R.)

(S80a) *niye:s* *niya:wéʔekih*
{*niy-uR-waʔ-iki*}
child PPL.indef:S-POSSESSIVE-DISTRIBUTIVE-be.PLURAL
'their children' (144; extracted from clause)

(S80b) *natí:weʔesikih*
{*nat-u'r-weʔes-iki*}
PPL.1S-POSSESSIVE-dog-be.PLURAL
'my dogs' (145; extracted from clause)

POSSESSIVE (Rood's term) is a verbal morpheme, as is clear in (S80a). In (S80b) it looks as though it might be a PSD affix on the noun (incorporated together with the noun), but (S80a) shows that it is a verbal morpheme. Perhaps it is a construct affix, registering but not indexing an argument; in that case it could be glossed PSD in our sense (§2.2 of main text).

In Wichita, therefore, the entire class of nouns is non-possessible. All possessive constructions use relativization.

S3.2.7 Algonquian[16]

Arapaho (arap1274, Montana; Cowell and Moss 2008:60–68) uses prefixal person indexation with any and all nouns (animate and inanimate, alienable and inalienable), the only differences in nouns being that inalienables are obligatorily possessed and some alienables have a special possessed stem formed by adding a PSD suffix. [17]

(S81) *híxon-o* *ne-t-íxon-eb*
bone-PL 1sg-EP-bone-PSD
'bones' 'my bone' (63)

(S82) *béé3ei* *hi-béé3ei-w*
owl 3poss-owl-OBV
'his/her owl' (62)

With inanimate possessors, however, possessive indexation is not possible, and a relative construction is used instead:[18]

(S83) Animate possessor: Inanimate possessor:
hi'-óó3 *hí-íooe-éihi-:noo-'*
3sg-leg 3.leg-AI.PASS-II-ØS
'his/her leg' 'its leg', lit. 'it has a leg' (e.g. a table) (64)

The relative construction does not define a class of non-possible nouns; rather, the possessor determines the construction type. (See main text §4.2.)

In addition, as in most Algonquian languages, a few nouns are non-possessible and have suppletive possessible counterparts. One of these, the possessible word for 'horse', also means 'pet' and can be used appositively in Arapaho (Cowell and Moss 2008:67):

(S84) *ho3* 'arrow' *wóxhoox* 'horse'
néic 'my arrow' *nó-toníhi'* 'my horse'
héic 'your arrow' *hó-toníhi'* 'your horse'
hiníc 'his arrow' *hí-toníh'o ~ hítoníhoʼ* 'his horse' (67)

(S85) *nó-toníhi'* *beníixóxko'ó'*
1sg-pet goat
'my pet goat'

(S86) *nó-toníhi'* *nih'óó3ouwóx*
1sg-pet pig
'my pet pig' (67)

---

[16] We thank Richard Rhodes for consultation on Algonquian.
[17] Transcription is in orthography; 3 = theta. Abbreviations: EP epenthetic; OBV obviative (case); AI animate intransitive, II intransitive inanimate; Ø inanimate; S subject.
[18] With verbs of the AI and II stem classes, relative and main clauses are identical (Cowell and Moss, 373).

Suppletive pairs are discussed in grammars of most Algonquian languages, e.g. Menomini (meno1252, Bloomfield 1962:42):

(S87) *anè:m* 'dog' (non-possessible)
ne-t-i:hsèh 'my dog'
1sg-EP-dog

Appositive treatment of non-possessibles seems not to be attested outside of Arapaho (where it seems to be a contact phenomenon: see main text §4.2).

S3.2.8 The U.S. Southeast[19]

Examples are attested from several of the diverse languages of the southeastern U.S, including one verbal appositive.

S3.2.8.1. Muskogean

The only case from the Muskogean family (Florida, Georgia, Alabama) that we have found is Creek (cree1270), originally spoken in northwestern Florida and nearby southwestern Alabama (now also Oklahoma). Creek has obligatorily possessed and optionally possessed nouns, and an additional set that, while not strictly non-possessible, "sound awkward with a possessive prefix" (Martin 2011:138). These include compounds, nominalizations, and nouns that are not ordinarily possessed. Instead they take an appositive construction with the noun 'thing':

(S88) *toɬ-sakká:ka* *ca-nâ:ki*
eye-sitting.in.it 1S.PAT-thing
'my glasses' (Martin 2011:138)

The same construction can be used with optionally possessed nouns as well.

S3.2.8.2. Iroquoian

In Cherokee (Virginia, Georgia), which comprises the Southern Iroquoian branch, human root nouns (i.e. chiefly kin terms; root nouns are underived basic nouns) can take possessive prefixes, but nonhuman root nouns cannot take any inflection and instead use an appositive construction with an empty or generic root and a possessive prefix indexing the person of the possessor:

(S89) *soógwíli* *agi-ajeéli*
horse 1B-POSS
'my horse' (Montgomery-Anderson 2015:134 citing Feeling 1975:17)

Montgomery-Anderson's term for this generic root is *possessive pronoun*. Kin terms are two-argument predicates that index the possessor with a Series B prefix (patient or S/O) and the kin referent with Series A (S/A).

(S90) *agi-ji* *ja-ji* *uu-ji*
1B-mother 2B-mother 3B-mother
my mother your mother her mother (145)

These can also mean 'She is my mother', 'She is your mother', 'She is her mother', functioning as predicates.

---

19 We thank Jack Martin and Aaron Broadwell for consultation on Muskogean and Cherokee (cher1273).

Though we have not seen appositive possession discussed in grammars of other Iroquoian languages, in fact all of the languages have non-inflectable words including some animal names, and these when owned can take an appositive construction using a possessible noun (in Northern Iroquoian typically *-tshenv* or *-itshenv* 'domestic animal') (Marianne Mithun, pers. comm.). The Iroquoian system is distinctive in that it involves general non-inflectability of nouns and not just specifically non-possessibility.

S3.2.8.3. Siouan

Appositive constructions are found in two languages of the Southeastern Siouan branch, both of which went extinct without receiving full descriptions. Biloxi (bilo1248, Einaudi 1976) has three possessive classes: obligatorily possessed (kin terms, body parts), optionally possessed (a few nouns denoting intimate possessions such as 'house' and items of clothing, which are treated as inalienables when owned), and all other nouns, which cannot be inflected for person. How ownership of these non-possessibles was expressed is unknown.

In Tutelo (tute1247) of the same branch (Oliverio 1996) inalienables are obligatorily possessed (kin terms, body parts) and are often but not necessarily used with possessive prefixes. Alienables are optionally possessed and take a longer possessive marker consisting of the person prefix plus a PSD prefix *-ta:-*. A handful of nouns also have an alternative construction with an appositive word consisting of the verb 'belong to' plus an indefinite suffix that marks it as a noun:

(S91) *wi - ta: - ku:čka:-i*
1sgP-PSD-child-INDEF
'my child' (140) (prefixal)

(S92) *wakučka* *wį - kítǫ - wi*
child 1sgDAT-belong-INDEF
'my child' (140) (appositive)

The word 'dog', and no other noun, takes a verbal appositive word 'own', which we interpret as a (finite) relative (relative clauses are not described for Tutelo). If the use with only 'dog' is not an accident of under-documentation, this is our only example of a noun lexically governing a possessive appositive word.

(S93) *čhǫk* *o - wa - hkí*^{n}*phi*
dog LOC-1sgA-own
'my dog' (lit. 'the dog I own')

(S94) *čhǫk* *o - ya - hkí*^{n}*phi*
dog LOC-2A-own
'your dog' ('the dog you own')

Non-possessible nouns are also found in the Dakotan subgroup of the Mississippi Valley Siouan branch of the northern Great Plains. Dakota ordinarily uses external rather than adnominal possession, marking the possessor on the verb with a person-number object index plus a dedicated prefix marking its possessive function (Boas and Deloria 1941:86–92, 128–130; Mithun 2001). Some nouns are non-possessible: "According to Dakota concepts certain objects, particularly natural objects and food, cannot be personal property" (Boas and Deloria 1941:90), and for those nouns the verb cannot take the possessive form and is reflexive instead. (Adnominal possessive

constructions do exist in Dakota, but we have not found a discussion of adnominal treatment of owned non-possessibles.) The Dakota material is important, as it shows that non-possessibility is independent of adnominal possessive morphology and is a more general lexical property of nouns themselves. We also surveyed Crow (Graczyk 2007), for which there is no mention of non-possessibles.

S3.3. Central America

S3.3.1. Seri (seri1257, isolate, northern Mexico; Marlett 1981)
Seri inalienables (kin terms, body parts) take possessive prefixes that index person. Alienables do not take possessive prefixes but use a verbal appositive, a relative clause with the verb 'own' in a nominalized form:

(S95)	*simalón*	*kiʔ*	*tro:ki*	*ya:*	*kiʔ*
	Cimalon	the	car	3.NMZ.own	the

'Cimalon's car' (69; extracted from clause)

(S96)	*kanóatax*	*ʔi-o-ya:t*	*koi*
	boats	1-NMZ-own	the

'our boats' (70; extracted from clause)

S3.3.2. Mayan
The Mayan family consists of some 40 languages mostly spoken in Guatemala. Mora-Marín (2021) surveys possession across the entire family. All Mayan languages use head-marked possession using person-number prefixes, and most have a variety of different types based on obligatory possession, possessibility with restrictions, non-possessibility, animate vs. inanimate possessor, intimacy of possession, and other distinctions, some of these marked by PSD and NPN suffixes of various types. One of these is what Dayley (1985:145) describing Tz'utujil (tzut1248) calls *abnormal possession:* a noun such as a body part or meronym takes unmediated possessive inflection in its alienable sense but requires a special suffix in the inalienable sense:

(S97) *kik'* 'blood': *nuu-kiik'* 'my blood' (e.g. to make sausage with)
n-kik'-eel 'my blood' (in my body)

Mora Marín shows that these suffixed forms can be analyzed as derivation, specifically abstractivization, and not as inflection.

Only a few Mayan languages have an appositive construction for non-possessibles, e.g. some natural phenomena (Mora Marín 29 and pers. comm.):

(S98)	K'ichee'	*qa-naan jab*	'our mother rain'	(1PL-mother rain)
		qa-naan uleew	'our mother earth'	(1PL-mother earth)
		qa-taat q'iij	'our father sun'	(1PL-father sun)
(S99)	Mam	*q-txuu jb'aal*	'our mother rain'	(1PL-mother rain)
		q-man q'iij	'our father sun'	(1PL-father sun)
		q-txu xjaaw	'our mother moon'	(1PL-mother moon)

or animals and food:

(S100a)	Yucatec	*inw-aalak'*	*pèek'*
		1sg-CL:animal	dog
		'my (pet) dog'	

(S100b)	*inw-ó'och*	*b'u'ul*
	1sg-CL:food	beans
	'my beans (ready to eat)'	

The Yucatec pattern is like the one found in several Uto-Aztecan and nearby languages, with appositive classifiers for animals and plant food. The examples from K'ichee' and Mam are different from any we have found elsewhere, classifying (if that is the right term) nouns not by their function or the way they were acquired or prepared but by what is probably a cultural or mythic connection or metaphor. Here too, an inalienable noun serves as classifier.

S3.3.3. Otomanguean

S3.3.1. Chichimeco Jonaz (chic1272, Otopamean branch of Otomanguean; central Mexico; Lastra de Suarez 1984) has a large number of inalienable possessive classes defined by stem changes in the noun when they take possessive prefixes. These nouns are mostly kin terms, body parts, and items of clothing. Alienables do not take possessive prefixes or the internal possessive marking, but occur with one of four appositive classifiers, which are inalienable nouns and do take stem changes. Examples with singular possessors (25):

		1sg	2sg	3sg
(S101)	CL:food	*nantʔé'*	*útʔe*	*utʔé*
	CL:clothing	*nuntʔú:*	*nírʔu:*	*nintʔú:*
	CL:animal	*námbæʔæ*	*ungwǽʔæ*	*úngwæʔæ*
	CL:thing	*námbihi*	*úngwihi*	*úm̲į̲hį* [20]

The noun *kúroho* 'rock' with singular possessors (25):

(S102)	1sg	*námbihi kúroho*
	2sg	*úngwihi kúroho*
	3sg	*úm̲į̲hį kúroho*

S3.3.2. Copala Triqui (copa1247, Mixtecan branch, southern Mexico; Broadwell 2011, 2016) has obligatorily possessed inalienables, possessible alienables, and non-possessible alienables. Inalienables have a juxtaposed possessor (S103a-b), which for some nouns can optionally be a clitic (c). (The noun in (S103a-b-c) has suppletive alienable and inalienable forms.)

(S103a)	*rej*	*xnii*
	father	child:ALIENABLE
	'the child's father'	

(S103b)	*ta'nii*	*so'*
	child:INALIENABLE	he
	'his child'	

[20] The original has a small underscore centered under the "m", indicating a lenis nasal (highly nasalized approximant).

(S103c) *tani-j*
child:INALIENABLE-1sg
'my child'

A small number of alienables are non-possessible and require an appositive classifier. There are two classifiers, one for animals and one for objects made of paper:

(S104a) *daán=j* *chuvee*
CL:animal=1sg dog
my dog

(S104b) *danj* *xnii* *libroó*
CL:paper child book
'the child's book'

S3.4. South America

S3.4.1. Cariban[21]

The Cariban family is fairly widespread in northern South America. The Glottolog classification lists 42 languages in 8 branches.

Cariban languages typically distinguish obligatorily possessed (typically kin terms, body parts, and a few others), optionally possessed, and non-possessible nouns, of which the latter always include domestic animals, growing plants, and some natural phenomena. (Tavares 2005:147–149 describes the semantics of Wayana non-possessibles as pertaining to the wild or non-cultural world, while possessibles belong to human culture, and their membership can be expanded as items of national and European culture become familiar in Wayana society.) Non-possessibles whose referents are owned take an appositive possessive construction with a classifier that behaves synchronically like an obligatorily possessed noun.

The classifiers are a fairly large class in languages of different branches, and often open in the sense that any relatively generic noun can be used in the same way. Classifiers can be used as appositives, e.g. Panare (enap1235) (using classifier 'liquid' as appositive):

(S105) *kape-ya* *y-úku-nh*
coffee-in 1-CL:liquid-PSD
'in my coffee' (Payne and Payne 2013:81)

or as independent nominals:

(S106) *Asa'* *t-ïkë* *mémpa-sejpa* *këj* *Toman*
two 3UNSP-CL:animal DI.steal-FUT AN.PROX Tom
'Tom is going to steal someone's two domestic animals' (86)

Several descriptions note that the classifier constructions can be discontinuous, with the classifier detached from the possessed noun, while in some languages they form a tighter phrase with strict order and no detachability (e.g. Meira 1999 for Tiriyó). Those describing the looser appositive

[21] For readability barred "i" of some of the Cariban sources is replaced with *ï*.

syntax generally describe the construction as something other than an NP and prefer the term "generic noun" to "classifier"; we call them all classifiers regardless of the closer vs. looser structure of the phrase.

Payne and Payne (2013:81–82) list 20 such classifiers for Panare (Pemong-Panare subbranch of Venezuelan Cariban); Koehn (1994) lists 9 for Apalai (apal1257, isolate branch); Tavares 2005:144 lists 13 for Wayana (waya1269, Wayanic subbranch of Guianan Cariban); Meira lists 7 for Tiriyó (trio1238, Taranoan subbranch of Guianan); Bruno (2003:69) gives only two, 'pet' and 'food', for Waimiri (waim1253, Yawaperi branch) (without, however, claiming that this is an exhaustive list). Classifier inventories for some of these are:

(S107) Apalaí (cited with possessive prefixes)

j-o-ty	1sg-meat.type.food-PSD
y-napy-ry	INCLdu-veg/fruit.type.food-PSD
ỹ-kyry-ry	1sg-field.produce-PSD
y-kyry-ry	1sg-thing-PSD
eky	3sg-pet/domestic.animal-PSD[22]
j-uhme	1sg-nut/corn/seed
i-karimo-ry	3sg-killed.game-PSD
apwahto-ry	2sg-firewood-PSD
z-u-ru	3sg-manioc.cake/bread-PSD

(S108) Wayana

ot(ï)	animal-based food
kaimo	game [animal]
akïï	farm animal; parasite; breed
anon(u)	body painting
(w)okï	beverage
ekï	pet
kïlïï	thing
muhunu	bait
pataa	place, village
kanpë	smoked animal-based food
nepïï	soft vegetable food
neme	juicy fruit/food
ka-top	thing

(S109) Waimiri

ieky	pet
wyty	food

For Macushi (macu1259, Pemong-Panare subgroup of Venezuelan branch; Carson 1982) only one classifier is found in more recent work:

(S110) *-ekïng* pet

but Carlson and Payne (1989:103) note that an older source (Williams 1932) also has a classifier

[22] Glossing *sic.* The grammar does not identify 3sg or PSD morphemes in this word.

for food.

We have found little discussion of classifier origins in Cariban. Synchronically, all are syntactic nouns and mostly inalienables. In Panare (Payne and Payne 2013:85) and Wayana (Tavares 2005:145) a few of them are verbal in origin (in Wayana those are nominalized verbs). The only source mentioned for new classifiers in open systems is nouns (usually with fairly generic semantics).

S3.4.2. Arawak

Arawak is an old and widespread family that once covered much of northern Amazonia and several Caribbean islands. The languages typically have head-marked possessive morphology, and nouns are usually divided into possessive classes based on obligatoriness of possession and the presence of PSD suffixes when possessed and/or NPN suffixes when unpossessed. Non-possessibles are generally not mentioned in grammars and where discussed at all they are few and there is no mention of classifiers or appositives. Most Arawak languages have a system of classifiers that are not possessive but have more or less gender-like functions, appearing on various modifiers in agreement with the head noun.

Examples from Yanesha' (yahe1238) showing the various classes (Duff-Tripp 1997:30–34):

(S111) Obligatorily possessed: Person-number prefix plus noun, no suffix:

po-ʔse (3sg-brother) 'her brother'

(S112) Usually possessed; NPN when not possessed.

oñ-ets (head-NPN) 'a head' *p-oñ* (3sg-head) 'his/her head'

Optionally possessed. Animates (also some relational terms and terms for edibles), usually have PSD when possessed. Inanimates are without PSD.

(S113)	*ochec*	'dog'	
	p-ochc-ar	(3sg-dog-PSD)	'his/her dog'
	cac	'fish'	
	po-cac-ar	(3sg-fish-PSD)	'his fish; his catch of fish'
	noñt´[23]	'canoe'	
	poʔ-noñt´	(3sg-canoe)	'his/her canoe'

Non-possessibles are chiefly *chesha'* 'child' and *huocchanesha'* 'orphan'.

When an inalienably possessed noun is additionally owned, the noun takes two possessive prefixes:

(S114)	*pa-ʔmeʔ*	*no-pa-ʔme-r*
	3sg-egg	1sg-3sg-egg-PSD
	'its egg' (i.e. the chicken's)	'my egg' (chicken's egg that I own)

[23] Duff-Tripp uses a tilde over all palatalized consonants. We write *t´*, etc. for letters we cannot place a tilde over.

Achagua (acha1250, Wilson 1992:65) has two possessive classifiers, animate and inanimate, used with alienable nouns. The classifier takes a possessive prefix if the possessor is a pronoun.

(S115) *hi-íhiža* *éema* *Manuél'i* *íhiža* *éema*
2sg-POSS horse Manuel POSS horse
'your horse' 'Manuel's horse'

(S116) *l'i-šínaa* *páiniya* *wašiál'ikuá-eži* *šínaa* *páiniya*
3sgM-POSS machete man-SG POSS machete
'his machete' 'the man's machete'

Achagua also has a system of 12 numeral classifiers. The appositive classifiers above are not part of that system (Wilson's term for them is *posposición de pertenencia*.)

Piapoco (piap1246, Reinoso 1999:92–98) also has an appositive possessive classifier. Inalienables (kin terms, body parts, plant parts) are obligatorily possessed. Alienables, if owned, take a PSD suffix together with the possessive prefix. Non-possessibles cannot take possessive prefixes or the PSD suffix. When owned they can optionally take the appositive possessive noun:[24]

(S117) *tzema* 'tobacco'
nu-azu tzema (1sg-POSS tobacco) 'my tobacco'

In Nanti (nant1250, Kampa branch; Michael 2008:297–301) the usual Arawak system is undergoing change. Possessive prefixes are obligatory with inalienables (which are further distinguished by being able to be incorporated into verbs, with the possessor marked by verbal person markers). Alienables take the same possessive prefixes and, optionally, the PSD suffix. The pan-Arawak NPN suffix that derives alienables from inalienables has been lost; instead, Nanti speakers use the inclusive possessive marker: *a-gito* (INCL-head) 'our.INCL head; a head'.

S3.4.3. Tupian

Tupian is an old family widespread in Amazonia and nearby. We have surveyed only the major branch, Tupi-Guarani.

Nearly all Tupi-Guarani languages distinguish inalienables (obligatorily possessed), which include kin terms, body and plant parts, and some intimate possessions such as clothing; alienables (optionally possessed); and non-possessibles, and grammars generally mention these three types and cover inalienables and alienables. The possessive marking for these is generally identical (except for the obligatory vs. optional nature), but the treatment of nouns without possessors or with unspecified possessors differs: inalienables have dedicated unspecified marking, while alienables instead use the first person inclusive for the same meaning (e.g. Guarani (para1311): Gustafson 2014:328). Just how unspecified possession of inalienables is marked varies. In Kamaiurá (kama1373, Seki 2000) and Tapiete (tapi1253, Gonzalez 2005:111) there is a dedicated UNSP possessor prefix *t-* in the paradigm of person-number prefixes; in Wayampi (waya1270) the marking is initial consonant alternations or vowel loss in the noun, e.g. *-poã* 'medicine': *e-poã* 'my medicine', *moã* 'medicine' (Jensen 1998:500).

---

[24] Wilson illustrates only with non-possessibles that when owned can be treated as either alienable (with classifier) or inalienable (with possessive prefix and no PSD), without discussion of the semantic difference. When 'tobacco' is treated inalienably the possessed form is *nu-tzema* (1sg-tobacco) 'my tobacco'.

While grammars generally identify a non-possessible class of nouns, they are less likely to discuss the marking of owned non-possessibles. Kukama-Kukamiria (coca1259, Vallejos 2016) has no person agreement but simply preposes a possessor to the head, creating an identifiable type of NP but not alienable vs. inalienable classes of nouns. Kin terms that vary for ego's gender (such as 'son' and 'daughter') have a "strong tendency" (114) to have a possessor; those without gender reference are optionally possessed. Non-possessibles would not be grammatically identifiable in such a system. Tapiete (tapi1253, Gonzalez 2005) has affixal possessive marking, which is obligatory for inalienables and optional for alienables, but perhaps no firmly grammaticalized class of non-possessibles; possessive morphology is pragmatically odd but elicitable for nouns denoting wild animals, air, earth, etc. For neither of these languages do there appear to be appositive possessive nouns.

For Paraguayan Guarani, Velasquez-Castillo (1996:9–85) shows that possessive predication is usually non-verbal for inalienables but verbal for alienables:

(S118) *(Che)* *che-memby-ta*
1sg 1sg.INACTIVE-offspring-FUT
I will have a child. (Velasquez-Castillo 66)

(S119) *A-reko* *Maria* *mesa* *che-roga-pe*
1sg.ACTIVE-have Maria table 1sg.INACTIVE-house-in
I have Maria's table at home. (76)

and equational predication of a possessed noun generally involves alienables:

(S120) *Ko-mesa* *che-mba'e*
this-table 1sg.INACTIVE-thing
This table is mine. (alienable noun) (84)

(S121) * Ko-memby che-mba'e
this-child 1sg.INACTIVE-thing
This child is mine. (inalienable noun) (84)

Her explanation relies on iconicity: less conceptual distance means less formal distance, so the inherent relationality of inalienables favors lack of an overt verbal form. See the discussion of appositives as predicative in §4.8 of the main text: we suggest that that there is an implicational hierarchy for null manifestation of a copula, and a first step in the hierarchy is predicative possession with an inalienable noun.

Some languages do have appositives, typically one for animals or two for domestic vs. wild animals. Emerillon (emer1243, Rose 2003) has only one, for domestic animals:

(S122) *de-le-iba* *zawal*
2sg-LINK-animal dog
'your dog'

Aikhenvald (2012:291) says that most Tupi-Guarani languages distinguish classifiers for domestic vs. wild animals. Only in Kamaiura, carefully described in Seki (2000), have we found a large system of appositive classifier nouns (302–303):

(S123) *-emijat* prey, game (lit. 'lo que é apanhado' = 'what's caught')
-emi'u food ('what's ingested')
-eymap domestic animal, pet
-yet canoe; vehicle, means of transport
-ywyrapat bow, weapon
-yru covering, wrap, clothing
-pyt body, house
-kyap sleeping place
-ata fire
-apo artifact, accessory

Note that these include prototypical non-possessibles such as prey and domesticated animals, and meanings that are commonly found among inalienables such as clothing, weapons, and houses. Not all of these are explicitly identified as non-possessibles, which (p. 54) are listed as including animals, plants, natural phenomena, and persons, and artifacts and tools are explicitly identified as alienable. The appositive construction, then, is broader than the non-possessible class.

Examples of the appositive construction are all translated as predicative (302):

(S124) *paku-a* *je = r - emijat*
paca-N 1sg-LINK-prey
'The paca is my prey (game animal)'

(S125) *ini -a* *je=kyap*
net-N 1sg-sleeping.place
'A net is my sleeping place'

(S126) *wararwijaw-a* *je =r -eymap*
dog -N 1sg-LINK-pet
'The dog is my pet'

Seki paraphrases *-emijat* 'game animal' and *-emi'u* 'food' as with verbal locutions: 'what is caught/hunted' and 'what is ingested' (302). We note that *-emijat* 'game animal' and *-eymap* 'domestic animal' have cognates elsewhere in the family (e.g. Jensen 1998:503) and these are the ones that Aikhenvald reports as most common in the family.

We conclude that the typical and probably reconstructable Tupi-Guarani system had appositive classifiers for game animals and domestic animals. The system has shrunk or disappeared in a few languages, and has grown considerably in Kamaiurá, where a more widely used appositive construction for equative predicative possession ('X is my Y') is used and perhaps conventionalized for owned non-possessibles.

S3.4.4. Matacoan

Matacoan is a small family found mainly in the Gran Chaco and east Andean region of Argentina.

Wichi Lhomtes (wich1261, Argentina; Nercesian 2014:167) uses direct person-number prefixation with inalienables, but with alienables it attaches the person-number index to a classifier: *ka-* for inanimates, especially instruments and tools but also others, and *lo=* for domesticated animals. *ka-* is only a prefix, but *lo=* can also be used as an independent noun 'animal'.

(S127a) *n' -ka-husan*
1sg-CL-axe
'mi hacha' (168)

(S127b) *n' -lo=mitsi*
1sg-CL=cat
'mi gato' (168)

(S127c) *la-lo=y*
3-CL=PL
'los animales' (169; extracted from clause example)

S3.4.5. Guaycuruan
Guaycuruan is a small family spoken in the Gran Chaco area of northern Argentina. Kadiweu (qomm1235, Sandalo 1995) has obligatorily possessed inalienables, which directly add a person-number prefix. [25]

(S128a) *l-Geladi*
3-house
'his house'

(S128b) *Gad:-akilo*
2-head
'your head' (56)

Alienables ordinarily have no prefix; they can be possessed, but then must have a classifier or what we gloss PSD between the person prefix and the noun root. There are three classifiers: female domestic animal, non-female domestic animal, and generic (all others).

(S129a) *l-wiGadi* *apolikGanGa*
3-CL:animal horse
'his horse'

(S129b) *l-wiqate* *apolikGanGa*
3-CL:female.animal horse
'his female horse'

(S129c) *Gad:-neb:i* *aqi:di*
2-CL:generic river
'your river'

The PSD prefix *n-* may be a reduced form of the generic classifier (Sandalo 57).

(S130) Gad:-n-aqi:di
2-PSD-river
'your river'

[25] Sandalo gives examples in both phonemic and more abstract transcription. We use only the more abstract one.

Two classifiers can co-occur (for emphasis: 57)

(S131) *i-wiGadi* *i-neb:i* *apolikGanGa*
1-CL:animal 1-CL:general horse
'this horse of mine' (57)

There are some nouns that cannot be possessed (even with classifiers). "If they do occur with possessive markers, the meaning changes" (58). The only example given is *epenay* 'moon' : *i-n-epenay* (1-PSD-moon) 'my month' (the month I was born in).

S3.4.6. South American isolates

S3.4.6.1. Itonama (iton1250, Bolivia; Crevels 2006, 2009) uses relativizing morphology on alienably possessed nouns, but with no verb root involved in the possession. Inalienables take a possessive prefix on the noun. Kin terms are verbs, and additionally take a subordinating prefix and verbal number marking.

(S132) Inalienable:

us-nu	'my nose'	
u'-nu	'your.M nose'	
ku'-nu	'your.F nose'	(2009:250)

(S133) Kin term:

ah - may - maye'ne	*ah - may - maye'ne-'cha'ke*	
3-SUBORD-father	3-SUBORD-father-MULTIPLE:ARG.	
'his father'	'their fathers'	(2006:163)

Alienables take the regular person-number prefixes followed by the relativizer/nominalizer *mi- / ni-,* which we gloss POSS. Forms of *uku* 'house' (2009:250):

(S134)

as - mi - ku	*dih - ni - ku*	*sih - ni - ku*
1sgPOSS-house	INCL-POSS-house	1pl-POSS-house
‘my house’	‘our-INCL house’	‘our-EXCL house’

S3.4.6.2. Urarina (northern Peru lowlands; Olawsky 2006)
Modern Urarina (urar1246), under Spanish influence, has lost aspects of its former alienability opposition, but the original system is still evident in traditional language. Inalienables (kin terms, body parts, and a few others) were obligatorily possessed, with person prefixes. 3sg *n-* is now frozen in the citation form of some of these nouns (350–351):

(S135)

alaarihja	'chest'	1sg	*k=alaarihja*	3sg	*n=alaarihja*
(n)anaae	'leg'		*k=anaae*		*n=anaae*
nïlïnï	'neck'		*ka=lïnï*		*raj nïlïnï*

Alienables were non-possessible and, when owned, used an appositive construction using a nominalized form *raj* of the verb 'receive' preceded by a juxtaposed noun or independent pronoun

(S136) *akaïrï* *raj* *batia*
3pl POSS pot
'their pots' (336)

(S137) *kača raj lureri*
man POSS house
'the man's house' (335)

The appositive word is now optional, or can take the possessive clitic of inalienables:

(S138) *kanï raj teru* ~ *kanï teru*
1sg POSS axe 1sg axe
'my axe' (353)

(S139) *ka=raj kanaanaj-ïrï=ra*
1sg=POSS child=PL=EMF (EMF = clause-final emphatic particle)
'my children' (338)

S3.4.6.3. Yurakare (yura1255, central Bolivia; van Gijn 2006) uses a person prefix for inalienables:

(S140) *ti-sibë*
1sg-house
my house

(S141) *shunñe a-sibë*
man 3sg-house
'the man's house' (116)

Most animal nouns cannot take the possessive prefixes but use a lexical classifier:

(S142a) *ti-talipa
1sg-chicken
'my chicken'

(S142b) *ti-tiba talipa*
1sg-pet chicken
'my chicken' (74)

Human nouns can only take possessive prefixes if they are kin terms or proper names (116–117; the grammar does not discuss whether non-kin human nouns can take appositives).

S3.4.6.4. Kwaza (kwaz1243, northwestern Brazil; van der Voort 2004)
Kwaza has a very large and probably open set of classifiers, nearly 150 (van der Voort 2004:177) which have a wide range of functions, one of which is possession. There is no alienability distinction. The possessor is in the nominative case, followed by either a nominalizer or a classifier. Van der Voort analyzes the nominalizer as a neutral classifier. In the following examples *-hỹ* is the nominalizer/neutral classifier and *-xy* is the classifier for such things as houses.

(S143) *'si-dy-hỹ a'xy*
1sg-GEN-NMZ house
'my house'

(S144) *Tawi'wi-dy-hỹ a'xy*
T.-GEN-NMZ house
'Tavivi's house'

(S145) *(a'xy) si-dy-xy*
house 1sg-GEN-CL:house
'my house'

(S146) *Tawi'wi-dy-xy*
T.-GEN-CL:house
'Tavivi's house'

These examples are from pp. 130–131; for other nouns with other classifiers see 181–186.

Since there is no alienability distinction, and since the possessor is nominalized, Kwaza can be described as a language with exclusively appositive possession using classifiers.

S3.5. The Pacific

S3.5.1. Oceanic

Most languages of the Oceanic branch of Austronesian contrast what the Austronesianist tradition calls *direct* and *indirect* possession, largely corresponding to our *inalienable* and *alienable* respectively. Lichtenberk (2018) gives a detailed survey of possessive constructions and their evolution. Lynch et al. (2002) is an earlier survey of just Oceanic. Proto-Oceanic had a small closed set: POc *ka- 'food', *m(w)a- 'drink', *na- general. These have no recoverable lexical source but may possibly be related to demonstratives (Lynch et al. 77). Whatever their sources, in modern languages they apparently are inalienable nouns in synchronic morphology, taking possessive affixes, showing that they are also to be found in the closest sister group to Oceanic, the South Halmahera-West New Guinea branch. Outside of Oceanic, Proto-South Halmahera-West New Guinea had a two-classifier system, *na- 'food' and *ri- general; the *na- classifiers of the two branches may be cognate (Lichtenberk 2018:186–190). Donohue and Schapper (2008) find other, more distantly related Austronesian languages with classifiers

With direct possession, the head noun takes a person-number possessive affix. This pattern is used on kin terms, body parts, other parts, and other prototypical inalienables. With indirect possession, the possessed noun cannot take possessive person affixes; instead, a classifier carries the person-number affixes and is syntactically in apposition to the possessed noun. Classifiers do not classify nouns rigidly; there is often freedom to choose a classifier based on the semantics. See main text §1 for Fijian examples and §2.2 for Paamese examples.

Larger inventories of possessive classifiers are found in three separate subgroups of Oceanic: Mussau (St. Matthias branch; the branch comprises Mussau (muss1246) and one very similar but nearly undescribed language); the Nuclear Micronesian branch (14 languages: Kosraean (kosr1238), Kiribati (gilb1244), Marshallese (mars1254), and 11 languages of the Pohnpeian-Chuukic subfamily); and Iaai (iaai1238, of the three-language Loyalties subgroup of New Caledonian). Since these three instances are widely separated phylogenetically and geographically, Lynch et al. reconstruct the abstract set of classifiers to Proto-Oceanic, but since it was an open class with changing membership and probably optional usage no individual classifiers can be reconstructed (79).

This larger set of classifiers again pattern as inalienable nouns, and many of them are derived from lexical nouns or verbs. E.g. Iaai (Lynch et al. 782–783; list selected from Ozanne-Rivierre 1976):

(S147) From nouns:

umwa-	houses, shelters	<	*uma* 'house'
nuu-	plants		*nu* 'coconut'
huu-	boats		*hu* 'boat'
tange-	baskets		*tang* 'basket'
mëni-	power		*mën* 'power'
waii-	reefs		*wai* 'reef'

(S148) From verbs:

hlogu-	fires	*hlök* 'warm oneself'
hicö-	things to be chewed	*hic* 'chew'
bicö-	headgear	*bicâ* 'wear on the head'
haalee-	animals	*haatr* 'look after'
döö-	spears	*dââ* 'pierce'

(S149) No known lexical source:

a-	food
bele-	drink
hnââ-	passive [undergoer of nominalized verb]
hne-	agent [of nominalized verb]
hanii	catch (of fish, game)
hwa-	noise, voice
iie-	piece for chewing
ii-	land
dee-	road, path
anyi-	general

Examples:

(S150) *wââ* *a-n* *thaan*
fish POSS:FOOD-3sg chief
'the chief's fish (to eat)' (783)

(S151) *köiö* *bele-n* *moomo*
water POSS:DRINK-3sg woman
'the woman's water (to drink)' (783)

Note also that, orthogonal to possessive classification, many Oceanic languages have a separate system of classifiers used as numeral classifiers and/or attached to the nouns themselves. These are different in function and also in semantics: "the numeral classifiers are generally based on physical form, while the possessive classifiers are generally based on function" (Lynch et al. 42).

Harrison (1988) shows that Kiribati/Gilbertese, the only Micronesian language lacking possessive classifiers, uses appositive possessed nouns and nominalized verbs in a looser construction for alienables.

(S152) *nima-u* *te* *ran*
drink-1sg ART:PSD water
'water for me to drink' (70)

The verb is an object nominalization with the semantics of a headless relative ('what I drink') (1988:74). The article is a dedicated possessive article.

This construction is optional, used only when further specification (here, the intended use of the water) is wanted, while in the other Micronesian languages a possessive classifier is obligatory with non-possessibles. Harrison argues that the Gilbertese construction is archaic and reflects the ancestral construction which evolved into the classifier construction of the sister languages. Appositive words that tend to figure in the Gilbertese construction are mostly cognate to classifiers of the other Micronesian languages.

Are the Oceanic possessive classifiers nominal or verbal in origin? In individual languages some of them are described as nominal or verbal in origin (as for Iaai above), but this is synchronic derivation or synchronically transparent etymological relatedness. Harrison reconstructs a nominalized verb form as ancestral to the construction in Micronesian and notes that many of the classifiers are cognate within Micronesian. Lynch et al. do not mention whether they can be traced back within the other two branches, stating only that no Proto-Oceanic set of classifier words is reconstructable. In any case, given the considerable part-of-speech flexibility of major class lexemes in Oceanic and more generally Austronesian languages (Foley in prep., 2017, 2008; Himmelmann 2008, and much earlier discussion; Foley reconstructs flexibility of the great majority of lexemes as the Proto-Austronesian state), etymological work tracing parts of speech of classifiers may prove inconclusive and the question itself may not be particularly meaningful. It may be telling, though, that the classifier construction must have evolved independently in Micronesian and must have been verbal in origin there.

S3.5.2. Non-Oceanic Austronesian

All are geographically distant from each other. South Halmahera-Western New Guinea is the closest sister to Oceanic. The others are from entirely different branches of Austronesian.

S3.5.2.1. South Halmahera-Western New Guinea: Donohue and Schapper (2008); s.a. Lichtenberk (2018). In Ambai (amba1265), most nouns use an appositive noun, but body parts and some kin terms take a possessive suffix directly. (There are further complications; see Donohue and Schapper 2008:320.)

(S153) Ambai (Donohue and Schapper 2008:320, citing Anceaux 1961, Silzer 1983, Price et al. n.d.)

ne-hu	*wafo*	*ne-mu*	*wiwing*
POSS-1sg	canoe	POSS-2sg	woman
'my canoe'		'your wife'	

awe-hu	*tama-mu(ai)*
leg-1sg	father-2sg
'my leg'	'your father'

In Selaru (sela1259) and Sawai (sawa1247), body parts are inalienable. Other nouns take one of two classifiers: food and general.

(S154) Selaru (Donohue and Schapper 2008:320, citing Coward 1990)

(a) *iblu-mw-ke*
skin-2sg-ART
'your skin'

(b) *wasi-mw* *hahy-desy-ke*
CL:GENERAL-2sg pig-that-ART
'your pig there', 'that pig of yours'

(c) *hina-mw* *hahy-desy-ke*
CL:EDIBLE-2sg pig-that-ART
'your pork there', 'that pork of yours'

S3.5.5.2. Others. Examples also occur in other branches: Banggai in the Celebic branch and Tugun and Kaitetu in different subbranches of Central-Eastern Malayo-Polynesian (Mark Donohue, p.c.).

S3.6. Non-Austronesian (Papuan) Pacific

Six non-Austronesian languages, phylogenetically and geographically distant from each other, have appositive classifiers. All are isolates or from small families.

S3.6.1. Abun (abun1252, isolate) (Berry and Berry 1999:77)

Inalienables (body parts and other parts of wholes) form possessive constructions with juxtaposition:

(S155) *ndar* *sye* *ne* *gwes*
dog big DET leg
'the big dog's leg' (77; extracted from clause)

(S156) *ji* *syim*
1sg arm
'my arm' (77; extracted from clause)

Alienables use an appositive word (called a linker in the grammar, following Croft (1990:32), and glossed POSS in the grammar)

(S157) *gap* *sye* *ne* *bi* *gan* *ge* *we*
rat big DET POSS young.one CL two
'the big rat's two young offspring' (78; extracted from clause)

(S158) *ji* *bi* *nggwe*
1sg POSS garden
'my garden' (78; extracted from clause)

Kin terms are alienable (78, 82):

(S159) *Rahel* *bi* *ai* *bi* *nyom*
Rachel POSS father POSS machete
'Rachel's father's machete' (82)

(S160) *An bi nji bi nggon bi nu*
3sg POSS brother POSS wife POSS house
'his brother's wife's house'

The Indonesian of Abun speakers calques the Abun possessive phrase, using the verb 'have' as possessive :

(S161) *saya punya rumah*
1sg have house
'my house' (81)

The fact that a verb is used suggests that the possessive morpheme *bi* of Abun is a verb (its source is not discussed in the grammar).

S3.6.2. Mpur (mpur1239, isolate) (Odé 2002)

Alienables use an appositive verb *tar* or *bi* 'possess' (61):

(S162) *n-tar jan*
1sg-POSS house
'my house' (62; extracted from clause)

(S163) *Lambert-a*[26] *a-bi ba(r)-in*
L.-3sgM 3sgM-POSS thing-grow
'Lambert's garden' (62; extracted from clause)

Inalienables (body parts, kin terms, and a few others) use a person-number prefix.

(S164) *a-wom*
3sgM-hand
'his hand'

(S165) *e-yen*
1pl-mother
'our mother'

(S166) *an-muk*
2sg-name
'your name'

S3.6.3. Bilua (buky1245, isolate) (Obata 2003:98–105)

In Bilua, inalienable nouns can take either direct (prefixal) or indirect (appositive) possession; alienables can take only appositive possession (34–35).

(S167) *ke=mama*
3pl father
'their father' (99; extracted from clause)

[26] The *-a* 3sgM on *Lambert* is autogender. Personal names are suffixed with *-n* (female) or *-a* (male), as are some other human nouns: 'mother', 'father', 'girl, woman', 'boy, man'; 'girlfriend', 'boyfriend' (63).

(S168) *o=baerebaere poso*
3sgM=friend PL.M
'his friends' (99; extracted from clause)

Inalienable nouns include kin terms, body parts, 'match', 'self', and "common nouns which have a special significance in the life and culture of Vella La Vella island people" (34–35): 'house', 'village/home', 'garden', 'island', 'friend', 'tribe'.

Indirect (alienable or inalienable; alienables can take only indirect possession) (35):

(S169) *Ilai=ko matu=ma pade*
I.=POSS.3sgF big=3sgF house (101)
'Ilai's big house'

(S170) *vo = ko ngi*
3sgM POSS.3sgF name
'his name' (75; extracted from clause)

ko (POSS.3sgF), the possessive word in these examples, is a 3sg distal demonstrative, *=vo* for singulative number and *=ko* for unspecified number (75; 100).

S3.6.4. Sulka (sulk1246, isolate) (Tharp 1996:80–81; Schneider 1962:259–261; Reesink 2005:179–183)

It appears that in Sulka part-whole terms and probably body parts and kin terms take direct head-marked prefixal indexation of the possessor. There are four different sets of possessor indices, for non-kin, kin, and two emphatic possessors.

(S171) a *kua-rik*
1sg-house
'my house' (80)

b *ko-nan*
1sg-mother
'my mother' (80)

c *kota-kom*
1sg.EMPH-knife
'my own knife' (81) (EMPH non-kin)

d *kot nan*
1sg.EMPH mother
'my own mother' (81)

Examples with overt possessors:

(S172) a *a-kom ka-rain*
sg-knife 3sg-handle
'the knife's handle' (118)

b *en* *ka-rai*
3sg 3sg-handle
'its handle'

Kin terms and parts of wholes, which appear in these examples, are not obligatorily possessed (118–119).

Other nouns use an uninflected form of the verb 'belong to' without argument indexation. It is not clear whether this is a prepositional or appositive construction. If appositive, it is atypical in that the putative appositive word does not bear person inflection. (It is not a relative construction, as those have relative pronouns and verbs inflected for person and TAM in the relative clause: 147–148. Schneider 1962: 259–261 considers the form a preposition. Reesink 2005:181 considers it possibly either a verb or a preposition.)

(S173) *a-kom* *to* *mkor* *e-Pruo*
sg-knife DEM.sg POSS:belong.to PN-Pruo
'Pruo's knife' (117)

(S174) *a-kom* *to* *mkor* *dok*
sg-knife DEM.sg POSS:belong.to 1sg
'my knife' (117) (PN = proper noun prefix)

S3.6.5. Bunaq (buna1278, Timor-Alor-Pantar; Donohue and Schapper 2008:322, citing Steinhauer 1977, 1991) uses prefixal possessive marking for inalienables and a single appositive word for alienables:

(S175) *n-up* *ni-e* *zap*
1-tongue 1-POSS dog
'my tongue' (inalienable) 'my dog' (inalienable)

Other languages in the family with this pattern include Blagar and Klon.

S3.6.6. Motuna (siwa1245, South Bougainville family, Bougainville Island, Papua New Guinea; Onishi 2012). Motuna has obligatorily possessed kin terms (*po-oro* [her-daughter] (89). Alienables cannot take possessive prefixes but have the option of either dependent-marked inflection or an appositive construction using one of the 51 classifiers of the language.

(S176) a *hoo* *howo* *peeko-na-pa*
ART.M house 3nonsg.POSS-LINK-CL:shelter
'their house' (in contrast to other houses) (244) (appositive)

b *peeko-ng* *howo*
3nonsgPOSS-M house
'their house' (245) (dependent-marked simple NP)

(The classifiers are used with both numerals and demonstratives, articles, etc.) There is a semantic or pragmatic difference: the appositive construction implies contrastive focus on the possessive NP or the possessive relationship. The classifiers are not independent nouns in Motuna, but they take the same possessive prefixation as the kin terms, so they are structurally like inalienable nouns.

This is the only example we have found where an appositive construction is just one option for alienables, and the choice has semantic consequences.

S3.6.7. Koiari (gras1249; Koiarian family, southern New Guinea; Dutton 1993, 1996). Animal names are non-possessible (no owned non-possessibles are reported). Dutton classifies animal names as proper names, none of which can occur in possessive constructions.

References

Aikhenvald, Alexandra Y. 2012. *The languages of the Amazon*. Oxford: Oxford University Press.

Anceaux J. C. 1961. *The linguistic situation in the islands of Yapen, Kurudu, Nau, and Miosnum, New Guinea*. The Hague: Martinus Nijhoff.

Bascom, Burton. 1982. Northern Tepehuan. In Ronald W. Langacker, (ed.), *Studies in Uto-Aztecan grammar, 3,* 271–393. Dallas-Arlington: SIL and University of Texas, Arlington.

Berry, Keith and Christine Berry. 1999. *A description of Abun: A West Papuan language of Irian Jaya*. Canberra: Australian National University.

Bloomfield, Leonard. 1962. *The Menomini language*. New Haven: Yale University Press.

Boas, Frans. 1927. *Keresan texts.* American Ethnological Society Publications 8:2 and 8:1. Leiden: Brill.

Boas, Franz & Deloria, Ella. 1941. *Dakota grammar* (23:2). Washington, DC: Government Printing Office.

Brambila, David. 1953. *Gramática rarámuri*. México: Editorial Buena Prensa.

Broadwell, George Aaron. 2011. Surrogate possession in Copala Triqui. Presented at HPSG Conference. MS, University at Albany.

Broadwell, George Aaron. 2016. La sintaxis de posesión par sustituto en trique de Copala. Presented at Coloquio sobre Lenguas Otomangues y Vecinos (COLOV). April 2016, Oaxaca, Mexico. http://www.academia.edu/24371419/La_sintaxis_de_posesion_por_sustituto_en_triqui

Bruno, Ana Carla. 2003. *Waimiri Atraoari grammar: Some phonological, morphological, and syntactic aspects.* PhD, University of Arizona.

Bugaeva, Anna. 2004. *Grammar and folklore texts of the Chitose dialect of Ainu (Idiolect of Ito Oda).* ELPR Publication Series A-045. Suita: Osaka Gakuin University.

Caballero, Gabriela. In preparation. A grammar of Choguitá Rarámuri. MS, University of California, San Diego.

Campbell, Lyle. 1985. *The Pipil Language of El Salvador*. Berlin: Mouton de Gruyter.

Carlson, Robert & Doris L. Payne. 1989. Genitive classifiers. In *Proceedings of the Fourth Meeting of the Pacific Linguistics Conference*, (ed.) Robert Carlson, Scott DeLancey, Spike Gildea, Doris L. Payne, & Anju Saxena, 87–119. Eugene: University of Oregon.

Carson, Neusa. 1982. *Phonology and morphosyntax of Macuxi (Carib).* PhD dissertation, University of Kansas.

Casad, Eugene H. 1984. Cora. In Ronald W. Langacker (eds) *Studies in Uto-Aztecan grammar, 4*, 153–459. Dallas: SIL.

Chiri, Mashiho. 1942(1973). Ainu gohoo kenkyū – karafuto hōgen o chūshin to shite [Studies in Ainu grammar – with an emphasis on the Sakhalin dialect]. In *Chiri Mashiho chosakushū* 3, 455–586. Tokyo: Heibonsha.

Chiri, Mashiho & Kyōsuke Kindaichi. 1936(1974). Ainu gohō gaisetsu [An outline of Ainu grammar]. In *Chiri Mashiho chosakushū* 4, 3–197. Tokyo: Heibonsha.

Coward, David Forrest 1990. An introduction to the grammar of Selaru. Phd dissertation, University of Texas At Arlington.

Cowell, Andrew & Alonzo Moss Sr. 2008. *The Arapaho language*. Boulder: University Press of Colorado.

Crawford, James M. 1966. *The Cocopa language.* PhD dissertation, University of California, Berkeley.

Crevels, Mily. 2006. Verbal number in Itonama. In Grażyna Rowicka & Eithne B. Carlin, eds., *What's in a verb? Studies in the verbal morphology of the languages of the Americas,* 160–170. (LOT Occasional Series, 5.) Utrecht: LOT.

Crevels, Mily. 2009. Itonama. In *Lenguas de Bolivia, II: Amazonía*, ed. Mily Crevels and Pieter C. Muysken, 233–294. La Paz: Plural.

Croft, William. 1990. *Typology and universals*. Cambridge: Cambridge University Press.

Davis, Irvine. 1964 *The language of Santa Ana Pueblo* (BAE Bulleti 191.53–190). Washington, DC: Government Printing Office.

Dayley, Jon P. 1985. *Tzutujil Grammar*. Berkeley-Los Angeles: University of California Press.

Dayley, Jon P. 1989. *Tümpisa (Panamint) Shoshone Dictionary*. Berkeley-Los Angeles: University of California Press.

Donohue, Mark & Annette Schapper. 2008. Whence the Austronesian possessive construction? *Oceanic Linguistics* 47(2). 316–327.

Duff-Tripp, Martha. 1997. *Gramática del idioma Yanesha' (amuesha)*. Peru: Ministerio de Educación and SIL.

Dutton, Thomas E. 1993. Possession in Koiari. *Language and Linguistics in Melanesia* 24(1). 39–58.

Dutton, Thomas E. 1996. *Koiari*. Munich: Lincom.

Einaudi, Paula Ferris. 1976. A grammar of Biloxi. New York: Garland.

Elliott, Eric Bryant. 1999. *Dictionary of Rincon Luiseño.* PhD, University of California, San Diego.

Feeling, Durbin. 1975. *Cherokee-English dictionary.* Tahlequah: Cherokee Nation of Oklahoma.

Foley, William A. 2008. The place of Philippine languages in a typology of voice systems. In Peter Austin & Simon Musgrave, eds., *Voice and Grammatical Relations in Austronesian Languages,* 22–44. Stanford: CSLI.

Foley, William A. 2017. Structural and semantic dependencies in word class membership. In Nick Enfield (ed.) *Dependencies in language: On the causal ontology of linguistic systems*, 179–195. Berlin: Language Science Press.

Foley, William A. In preparation. *The epidemiology of language: The evolution of word class categorialization in the Austronesian languages*.

González, Hebe Alicia. 2005. *A grammar of Tapiete (Tupi-Guarani).* PhD, University of Pittsburgh.

Gordon, Lynn. 1986. *Maricopa morphology and syntax*. University of California Press: Berkeley-Los Angeles.

Graczyk, Randolph. 2007 *A Grammar of Crow*. Lincoln: University of Nebraska Press.

Gustafson, Bert. 2014. Guaraní. In Pieter Muysken & Mily Crevels, eds., *Lenguas de Bolivia,* 307–368. La Paz: Plural Editores.

Harrison, S. P. 1988. A plausible history for Micronesian possessive classifiers. *Oceanic Linguistics* 27(1/2). 63–78.

Hill, Jane H. 2014. *A grammar of Cupeño*. Berkeley-Los Angeles: University of California Press.

Himmelmann, Nikolaus P. 2008. Lexical categories and voice in Tagalog. In Peter K. Austin & Simon Musgrave, eds., *Voice and grammatical relations in Austronesian languages,* 247–293. Stanford: CSLI.

Hopi Dictionary Project. 1998. *Hopi Dictionary*. Tucson: University of Arizona Press.

Jacobsen, William H., Jr. 1964. *A Grammar of the Washo Language.* Ph.D., University of California, Berkeley.

Jensen, Cheryl. 1998. Comparative Tupi-Guarani syntax. In Desmond C. Derbyshire & Geoffrey K. Pullum, eds., *Handbook of Amazonian Languages,* 489–616. Berlin: Mouton de Gruyter.

Koehn, Sally Sharp. 1994. The use of generic terms in Apalaí genitive constructions. *Revista latinoamericana de estudios ethnolingüísticos* 8.

Kroeber, A. L. & George William Grace. 1960. *The Sparkman grammar of Luiseño*. Berkeley-Los

Angeles: University of California Press.
Kubodera, Itsuhiko. 1977. *Ainu jojishi shin'yō seiden-no kenkyū* [The study of Ainu heroic epics and songs of gods]. Tokyo: Iwanami Shoten.
Lachler, J. 2006. *A grammar of Laguna Keres.* PhD dissertation, University of New Mexico.
Langdon, Margaret H. 1970. *A grammar of Diegueño: The Mesa Grande dialect*. Berkeley-Los Angeles: University of California Press.
Langacker, Ronald W., ed. *Studies in Uto-Aztecan grammar, vol. 1: An overview of Uto-Aztecan grammar.* Dallas and Arlington: SIL and University of Texas, Arlington, 1977.
Lastra de Suarez, Yolanda. 1984. Chichimeco Jonaz. In Munro S. Edmonson & Patricia A. Andrews, eds., *Supplement to the handbook of Middle American Indians: Linguistics,* Austin: University of Texas Press.
Lichtenberk, Frantisek. 2018. The diachrony of Oceanic possessive classifiers. In William B. McGregor & Søren Wichmann, eds., *The diachrony of classification systems,* 165–200. Amsterdam: Benjamins.
Liljeblad, Sven, Catherine S. Fowler and Glenda Powell. 2012. *The Northern Paiute-Bannock dictionary*. Salt Lake City: University of Utah Press.
Lynch, John, Malcolm D. Ross and Terry Crowley. 2002. *The Oceanic languages*. Richmond, Surrey: Curzon.
Marlett, Stephen A. 1981. *The structure of Seri.* PhD dissertation, University of California, San Diego.
Martin, Jack B. 2011. *A grammar of Creek (Muskogee)*. Lincoln: University of Nebraska Press.
Meira, Sergio. 1999. *A grammar of Tiriyó.* PhD dissertation, Rice University.
Michael, Lev. 2008. *Nanti evidential practice: Language, knowledge, and social practice in an Amazonian society.* PhD dissertation, University of Texas.
Miller, Wick R. 1965. *Acoma grammar and texts*. (UCPL 40.) Berkeley-Los Angeles: University of California Press.
Miller, Amy. 2001. *A grammar of Jamul Tiipay*. Berlin: Mouton de Gruyter.
Mithun, Marianne. 2001. The difference a category makes in the expression of possession and inalienability. In *Dimensions of possession*, ed. Irène Baron, Michael Herslund, and Finn Sørenson, 285–310. Amsterdam-Philadelphia: Benjamins.
Mixco, Mauricio. 1971. *Kiliwa grammar.* PhD dissertation, University of California, Berkeley.
Montgomery-Anderson, Brad. 2015. *Cherokee reference grammar*. Norman: University of Oklahoma Press.
Mora-Marín, David. 2021. Reconstructing possessive morphology in Mayan languages. To appear in *IJAL* 87.
Nakagawa, Hiroshi. 1995. *Ainugo Chitose hōgen jiten* [A dictionary of the Chitose dialect of Ainu]. Tokyo: Sōfūkan.
Nakayama, Toshihide. 2001. *Nuuchahnulth (Nootka) Morphosyntax*. (UCPL 134.) Berkeley-Los Angeles: University of California Press.
Nercesian, Verónica. 2014. *Wichi lhomtes: Estudio de la gramática y la ineracción fonología-morfología-sintaxis-semántica*. München: Lincom Europa.
Obata, Kazuko. 2003. *A grammar of Bilua*. Canberra: Research School of Pacific and Asian Studies, Australian National University.
Odé, Cecilia. 2002. A sketch of Mpur. In Ger P. Reesink (ed.) *Languages of the eastern Bird's Head*. 45–107. Canberra: Pacific Linguistics.
Olawsky, Knut J. 2006. *A grammar of Urarina*. Berlin: Mouton de Gruyter.
Oliverio, Giulia R. M. 1996. A grammar and dictionary of Tutelo. PhD dissertation, University of Kansas.
Onishi, Masayuki. 2012. *A grammar of Motuna*. München: Lincom Europa.
Ozanne-Riviere, Françoise. 1976. *Le Iaai: Langue mélanésienne d'Ouvéa (Nouvelle-Calédonie)*. Paris: SELAF.
Payne, Doris L. & Thomas E. Payne. 2013. *A typological grammar of Panare*. Berlin: De Gruyter

Mouton.
Press, Margaret L. 1980. *Chemehuevi: Grammar and lexicon*. (UCPL 92.) Berkeley-Los Angeles: University of California Press.
Price, David S., Tamara R. Price, Peter J. Silzer, Cheryl Silzer, & Nataniel Merasi. *Ambai-Indonesian dictionary.* n.d. MS, SIL, Papua Branch.
Ravinski, Christine. 2005. *Grammatical possession in Nuu-Chah-Nulth.* M.A. thesis, University of British Columbia.
Reesink, Ger P. 2005. Sulka of East New Britain: A mixture of Oceanic and Papuan traits. *Oceanic Linguistics* 44(1). 145–193.
Reinoso Galindo, Andrés Eduardo. 1999. *Elementos para una gramática de la lengua piapoco*. Bogotá: Ministerio de Cultura.
Rood, David S. 1976. *Wichita grammar*. New York/London: Garland.
Rose, Françoise. 2003. *Morphosyntaxe de l'émérillon: Langue tupi-guarani de Guyane française.* PhD, Université Lumière Lyon 2.
Rosenthal, Jane M. 1981. How Uto-Aztecan is the Nahuatl possessive? In Frances Karttunen, (ed.), *Nahuatl studies,* 182–214.
Sandalo, Filomena. 1995. *A grammar of Kadiweu*. Ph.D. dissertation, University of Pittsburgh.
Sapir, Edward. 1930. *Southern Paiute, a Shoshonean language*. Boston: American Academy of Arts and Sciences.
Satō, Tomomi. 2003. Sakata shiritsu mitsuoka bunko shozō Ezo-ki no Ainugo ni tsuite [Notes on an old Ainu vocabulary in the Sakata City Kōkyū Library]. *Hokkaidō Daigaku Bungaku Kenkyūka Kiyō* 111. 95-118.
Saxton, Dean. 1982. Papago. In Ronald W. Langacker, (ed.), *Uto-Aztecan grammatical sketches, v. 3,* Dallas and Arlington: SIL and University of Texas, Arlington.
Schneider, Joseph. *Grammatik der SulkaSsprache (Neubritannien)*. (Micro-Biblioteca Anthropos, 36.) Posieux: Anthropos Institut, 1962.
Seiler, Hansjakob. 1977. *Cahuilla Grammar*. Banning, CA: Malki Museum Press.
Seiler, Hansjakob. 1995. Possession and classifiers in Cahuilla (Uto-Aztecan). Egon Renner, Michael Dürr & Wolfgang Oleschinski, eds., *Language and Culture in Native North America: Studies in honor of Heinz-Jürgen Pinnow*, 211-225. Munich: Lincom.
Seiler, Walter. 1985. *Imonda, a Papuan language*. Canberra: Research School of Pacific Studies Australian National University.
Seki, Lucy. 2000. Gramática do kamaiurá: Língua tupi-guarani do Alto Xingu. Campinas - São Paulo: Editora da Unicamp & Imprensa Oficial SP.
Shaul, David Leedom. 2012. *Eastern Shoshone working dictionary*. MS.
Silzer, Peter James. 1983. *Ambai: An Austronesian Language of Irian Jaya, Indonesia.* Phd Dissertation, Australian National University.
Steinhauer, Hein. 1977. "Going" and "coming" in the Blagar of Dolap (Pura, Alor, Indonesia). *NUSA: Miscellaneous Studies in Indonesian and Languages of Indonesia* 4. 39–49.
Steinhauer, Hein. 1991. Demonstratives in the Blagar language of Dolap (Pura, Alor, Indonesia). In *Papers in Papuan linguistics*, ed. Tom Dutton, 177–221. Canberra: Pacific Linguistics.
Tavares, Petronila da Silva. 2005. *A grammar of Wayana.* PhD dissertation, Rice University.
Tharp, Doug. 1996. Sulka grammar essentials. In John M. Clifton (ed.) *Two non-Austronesian grammars from the islands*. 77–179. Ukarumpa: SIL.
Vallejos, Rosa. 2016. *A grammar of Kukama-Kukamiria: A language from the Amazon*. Leiden: Brill.
van der Voort, Hein. 2004. *A Grammar of Kwaza*. Berlin: Mouton de Gruyter.
van Gijn, Rik. 2006. A grammar of Yurakaré. PhD dissertation, Radboud University.
Velasquez-Castillo, Maura. 1996. *The Grammar of Possession: Inalienability, incorporation, and possessor ascension in Guaraní*. Amsterdam: Benjamins.
Voegelin, C. F. 1935. *Tübatulabal grammar.* (UCPAAE 34:2.) Berkeley: University of California Press.
Voegelin, Charles F. 1958. Working dictionary of Tübatulabal. *IJAL* 24.221-228.

Watahomigie, Lucille J., Jorigine Bender & Akira Y. Yamamoto. 1982. *Hualapai reference grammar*. Los Angeles: American Indian Studies Center, UCLA.
Willett, Thomas L. 1991. *A reference grammar of Southeastern Tepehuan*. Dallas: SIL and University of Texas at Arlington.
Williams, James. 1932. *Grammar, notes, and vocabulary of the Makuchi Indians of Guiana*. St. Gabriel-Mödlung.
Wilson, Peter J. 1992. *Una description preliminar de la gramatica del achagua (arawak)*. Bogotá: ILV [i.e. SIL].
